# Propargylated Purine Deoxynucleosides: New Tools for Fluorescence Imaging Strategies

**DOI:** 10.3390/molecules24030468

**Published:** 2019-01-28

**Authors:** Akkaladevi Venkatesham, Sambasiva Rao Pillalamarri, Flore De Wit, Eveline Lescrinier, Zeger Debyser, Arthur Van Aerschot

**Affiliations:** 1 Medicinal Chemistry, Rega Institute for Medical Research, Department of Pharmaceutical and Pharmacological Sciences, KU Leuven, Herestraat 49, 3000 Leuven, Belgium; venkat.pallavi2013@gmail.com (A.V.); sambasivarao.iict@gmail.com (S.R.P.); eveline.lescrinier@kuleuven.be (E.L.); 2 Laboratory for Molecular Virology and Gene Therapy, Department of Pharmaceutical and Pharmacological Sciences, KU Leuven, Kapucijnenvoer 33, 3000 Leuven, Belgium; flore.dewit@kuleuven.be (F.D.W.); zeger.debyser@kuleuven.be (Z.D.)

**Keywords:** click reaction, propargylated nucleosides, imaging, DNA visualization, fluorophores, ethynyl-2′-deoxyuridine (EdU), HIV toxicity, Mitsunobu reaction

## Abstract

In vivo imaging of biological processes is an important asset of modern cell biology. Selectively reacting fluorophores herein are an important tool and click chemistry reactions take a large share in these events. 5-Ethynyl-2′-deoxyuridine (EdU) is well known for visualizing DNA replication, but does not show any selectivity for incorporation into DNA. Striving for specific visualization of virus replication, in particular HIV replication, a series of propargylated purine deoxynucleosides were prepared aiming for selective incorporation by HIV reverse transcriptase (RT). We here report on the synthesis and preliminary biological effects (cellular toxicity, HIV inhibitory effects, and feasibility of the click reaction) of these nucleoside analogues.

## 1. Introduction

Visualization of biological processes via labeling of DNA or proteins using specific fluorescent markers has become a cornerstone of many cell biology studies. The standard methodologies herein use monoclonal antibodies either recognizing incorporated 5-bromo-2′-deoxyuridine (BrdU) for DNA studies or modified amino acids for proteins, where the antibodies are functionalized to provide a fluorescent signal. Many alternative strategies have been used, among which the use of thiol reactive probes for direct protein visualization and many other alternatives. Two excellent handbooks on molecular probes and fluorescence imaging provide a complete overview of all fluorescent labeling techniques [1,2].

A very different approach makes use of green fluorescent protein and its many variants, which can be used to genetically engineer specific proteins or organisms. For instance, this way retroviral replication was visualized for the first time in living cells [3]. This nice methodology, however, does not provide the means to selectively visualize replication of wild type viruses.

An alternative to fluorescence imaging to allow for visual understanding of biological processes within a living organism, is the use of bioluminescent imaging (BLI). BLI uses light emitted from luciferase-expressing bioreporter cells and its main use is the in vivo tracking of cell fate or the study of regulation of gene expression via reporter gene expression, which, however, is not the topic of our work [4].

As described above, analysis of DNA synthesis in the past was mainly based on incorporation of BrdU, which is detected based on recognition by specific antibodies, but requiring prior treatment of the samples to allow reaction of the incorporated BrdU with the antibodies [5,6]. In contrast, detection of replicational activity in vivo was accomplished for the first time in 2008, making use of 5-ethynyl-2′-deoxyuridine (EdU, **1**) [7]. The triple bond containing nucleoside incorporated well into growing DNA chains and allowed visualization of the latter, introducing fluorescent markers via click chemistry. Several small fluorescent azides hereto proved able to penetrate preparations of tissue and organ explants and to provide the well-known Cu(I)-catalyzed cycloaddition reaction. The method proved very popular, and a large number of studies in the past have focused on measuring the rate of mitochondrial DNA (mtDNA) replication, with S. Lentz et al. being the first to use EdU to visualize mtDNA biogenesis [8]. Its close analogue, 5-ethynyl-2′-deoxycytidine (EdC, **2**) likewise was developed as marker of cellular replication activity proving less cytotoxic [9]. However, Ligasova et al. more recently demonstrated that EdC is metabolized and incorporated into DNA as EdU, with toxicity being related to the extent of its total incorporation [10].

EdU (and EdC) being valuable compounds for in vivo analyses of DNA replication, are recognized and incorporated into DNA by various polymerases, and therefore lack the required selectivity for in vivo visualization of viral replication. Indeed, EdU is well incorporated by the host mitochondrial DNA polymerase provoking high off-target labeling of the cytoplasm and secondly, it is impossible to discriminate viral DNA in the nucleus due to the incorporation of the functionalized nucleoside by the host DNA polymerase. To develop virus-specific chromophores, the clickable nucleosides should not be recognized by the cellular DNA polymerases, but selectively incorporated by the viral polymerase. We therefore opted for a series of propargylated nucleosides, whilst aiming in the first place for the selective incorporation into HIV viral DNA and lowering the off-target labeling of cellular DNA. The choice for propargyl moieties was dictated as a compromise in the following trade-off: having a small distance between the triple bond and the heterocycle should allow the click reaction to run smoothly, while having too long extensions on the nucleosides potentially could hamper their functionalization into the required triphosphates to allow incorporation into DNA. In addition, the propargyl alcohol and amine are cheaply available materials. The ultimate goal of this project was the visualization of replicating single particles. Indeed, double-stranded copy DNA (cDNA) is produced by the viral reverse transcriptase (RT). This cDNA is subsequently integrated into the host chromosome by the viral integrase (IN). Using an engineered virus containing a GFP-modified integrase (IN-eGFP), we at present can identify single viral complexes in infected cells [11], but we are unable to identify whether they contain reverse transcribed DNA, nor can this technique be used for visualization of wild type viruses.

**Figure 1:**
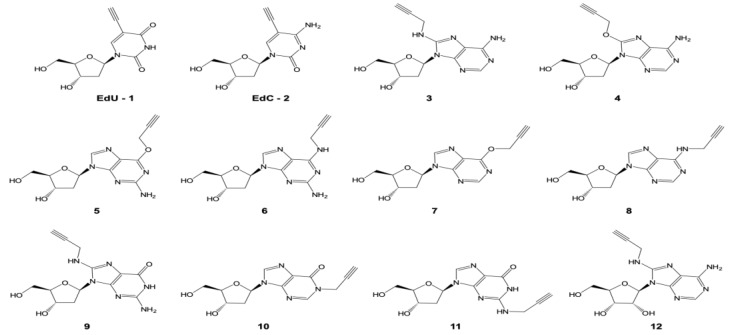
Structures of the envisaged propargyl containing purine 2′-deoxynucleosides for visualization studies (**3**–**10**), along with well-known 5-Ethynyl-2′-deoxyuridine (EdU) (**1**) and 5-ethynyl-2′-deoxycytidine (EdC) (**2**) and two literature described propargyl modified purine derivatives (**11**–**12**).

A series of seven propargylated deoxynucleosides (Figure 1, **3**–**10**) were conceived, synthesized, and evaluated for their cell cytotoxic and HIV antiviral effects in a preliminary effort to obtain a clickable nucleoside analogue, which would be selectively incorporated into HIV-DNA. Only a few propargyl-containing purine nucleosides have been described before. Introducing the propargyl moiety on the 2-amine position of dG providing *N*^2^-(2-propyn-1-yl)-2′-deoxyguanosine (**11**) does not look very appealing as it most probably will disturb base pairing [12]. However, also the purine analogues **5** and **8** were described by the same authors albeit to provide clickable nucleosides for incorporation of boron clusters into DNA using pre-functionalized phosphoramidites. In addition, we unintentionally obtained **10** almost exclusively upon alkylation of 2′-deoxyinosine (dI) under Mitsunobu conditions. Finally, the 8-propargylamino substituted adenosine **12**, being the ribose analogue of **3**, was already prepared in the past to introduce a clickable Rhodamine dye [13].

## 2. Results

### 2.1. Chemistry

**Scheme 1:**
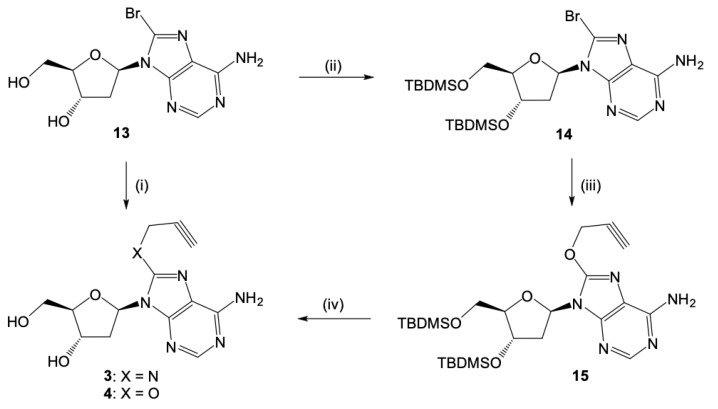
(i) Propargylamine, CaCO_3_, EtOH, 70–80 °C, 14 h (72% of **3**); (ii) TBDMSCl, imidazole, dry DMF, rt, 24 h (81%); (iii) propargylalcohol, *n*BuLi, THF, −40 °C to rt, 24 h (91%); (iv) TBAF, THF, rt, 3 h (85% of **4**).

The synthesis of 8-propynylamino-(**3**) and 8-propynyloxy-2′-deoxyadenosine (**4**) involves the formation of a carbon-hetero bond at the C8 position of the nucleobase. The latter is commonly achieved using nucleophilic substitution on the 8-brominated derivative (Scheme 1, **13**). Synthesis of **4** required prior protection of both hydroxyl moieties as tert-butyldimethylsilyl (TBDMS) ethers using TBDMSCl and imidazole in dry dimethylformamide (DMF) at rt for 24 h, affording **14** in 81% yield. Nucleophilic substitution of the 8-bromine with propargyl alcohol was accomplished using nBuLi in tetrahydrofuran (THF) at −40 °C to rt for 24 h affording **15** in 91% yield [14]. Deprotection of both silyl groups using tetrabutylammonium fluoride (TBAF) in THF at rt for 3 h furnished **4** in 85% yield. Direct substitution with propargyl amine in presence of CaCO_3_ in EtOH at 70–80 °C for 14 h gave **3** in 72% yield [13].

**Scheme 2:**
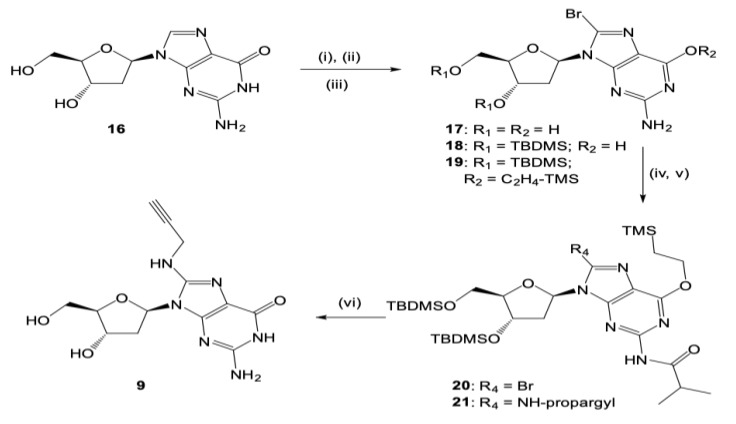
(i) N-bromosuccinimide (NBS), CH_3_CN: H_2_O, rt, 45 min (91% of **17**); (ii) TBDMSCl, imidazole, dry DMF, rt, 14 h (87% of **18**); (iii) 2-TMS-ethanol, TPP, diisopropyl azodicarboxylate (DIAD), 1,4 dioxane, 40 °C, 24 h (65%); (iv) isobutyryl chloride, anh. pyridine 0 °C to rt, 3 h (85%); (v) propargyl amine, Pd_2_(dba)_3_, *rac*BINAP, Cs_2_CO_3_, 100 °C, 14 h (61%); (vi) TBAF, THF, rt, 14 h; 7 *N* NH_3_ in MeOH, 60 °C, 14 h (65% of **9** overall for two steps).

C8-modification of 2′-deoxyguanosine to provide **9** was less straightforward, as complexation of the palladium catalyst by interaction through *O*^6^ and *N*^7^ positions of the nucleobase was suggested to cause the poor reactivity of guanosine towards palladium catalyzed cross-coupling reactions [15]. To inhibit the complexation of metals by guanosine derivatives, the *O*^6^ position was protected as a TMS-ether. This fully protected dG-derivative was synthesized starting with bromination of **16** with N-bromosuccinimide (NBS) [16] in a (1:4) H_2_O:CH_3_CN mixture in good yield (Scheme 2). The 5′- and 3′-hydroxyl groups of **17** were protected as TBDMS ethers to give **18** in 87% yield, while the TMS ether group was introduced at *O*^6^-position by Mitsunobu reaction involving 2-TMS-ethanol, diisopropyl azodicarboxylate (DIAD) and triphenyl phosphine (TPP) in dioxane at 40 °C for 24 h to afford **19** in 65% yield. The exocyclic amine was protected by treatment with isobutyryl chloride in pyridine at rt for 3 h to give the key intermediate **20** in 85% yield. Buchwald–Hartwig amination [17] of **20** using propargyl amine in presence of Pd_2_(dba)_3_, 2,2′-bis(diphenylphosphino)-1,1′-binaphthyl (BINAP) and Cs_2_CO_3_ in dioxane at 100 °C for 14 h furnished **21** in 61%yield. Finally, all three silyl moieties can be removed in a single deprotection step using TBAF in THF, followed by deprotection of the isobutyryl group using 7*N* NH_3_ in MeOH at 60 °C for 14 h to obtain the final compound **9** in 65% yield. Use of *N*^2^-Boc protection instead proved inappropriate, leading to depurination during the final deprotection step with 90% aq. TFA at rt for 14 h. Likewise, introduction of an 8-propynyloxy moiety on compound **20** proved unsuccessful under Buchwald–Hartwig [18] conditions. Attempts using either propargyl alcohol, Pd_2_(dba)_3_, t-BuDavePhos, Cs_2_CO_3_, dioxane at 100 °C or Pd_2_(dba)_3_, t-BuDavePhos, K_3_PO_4_, toluene at 100 °C led to degradation of the starting material.

**Scheme 3:**
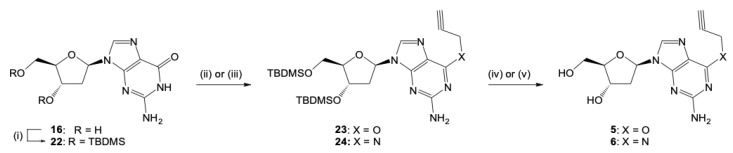
(i) TBDMSCl, imidazole, DMF, rt, 24 h (84%); (ii) propargyl alcohol, TPP, DIAD, dioxane, rt, 14 h (49% of **23**); (iii) TIBSCl, Et_3_N, cat DMAP, DCM, 48 h; propargylamine, EtOH, glass sealed tube, 100 °C, 14 h (55% overall for both steps to **24**); (iv) TBAF, THF, rt, 2 h (85% of **5**); (v) NH_4_F, MeOH, 70–80 °C, 24 h, (81% of **6**).

Synthesis of the deoxyguanosine analogues **5** and **6** requires introduction of the carbon-hetero bond at the C6 position of the nucleobase. This is commonly achieved using either Mitsunobu [19] or nucleophilic substitution [20] reactions as shown in Scheme 3. Hereto, **16** was reacted with TBDMSCl, imidazole in DMF at rt for 24 h to give **22** in 84% yield. The propynyl moiety was then introduced at *O*^6^ by Mitsunobu reaction involving propargyl alcohol, DIAD and TPP in dioxane at rt for 14 h to give **23** in 49% yield, while a propynylamino moiety was introduced at C6 following activation of **22** at 6-OH position with 2,4,6- triisopropylbenzenesulfonyl chloride (TIBSCl), DMAP and NEt_3_ in DCM at rt for 48 h. Subsequent nucleophilic substitution with propargyl amine in EtOH at 70–80 °C for 24 h afforded the 2,6-diaminopurine derivative **124** in 55% (combined yield). Deprotection of both silyl moieties using TBAF in THF at rt for 2 h for **23** afforded **5** in 85% yield, while **24** was deprotected using NH_4_F in MeOH at 70–80 °C for 24 h providing the diaminopurine **6** in 81% yield.

**Scheme 4:**
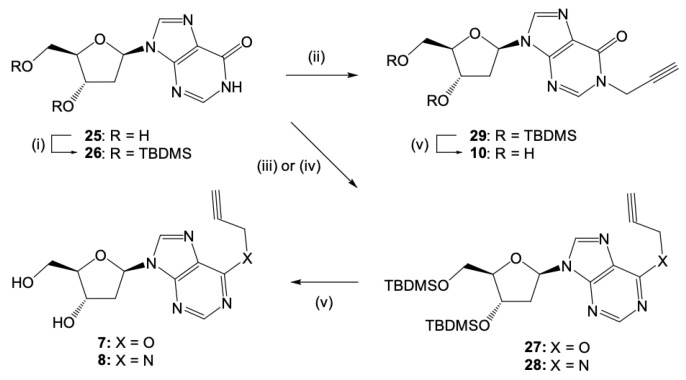
(i) TBDMSCl, imidazole, DMF, rt, 24 h (92%); (ii) propargyl alcohol, TPP, DIAD, THF, rt, 14 h (78% of **29**); (iii) BOP, Cs_2_CO_3_, propargyl alcohol, THF, rt, 5 h (91% of **27**); (iv) TIBSCl, Et_3_N, cat DMAP, DCM, 48 h; Propargyl amine, EtOH, glass sealed tube, 100 °C, 14 h (57% overall for both steps to **28**); (v) NH_4_F, MeOH, 70–80 °C, 24 h (71%, 75% and 80%, respectively for **7**, **8** and **10**).

Synthesis of the C6-modified 2′-deoxypurine analogues **7** and **8** was attempted analogously to the 2′-dG congeners via either Mitsunobu reaction or nucleophilic substitution reaction at C6 on dI (**25**) as shown in Scheme 4. Protection of **25** with TBDMSCl, imidazole in DMF at rt for 24 h afforded **26** in 78% yield. Alkylation under Mitsunobu conditions afforded almost exclusively the *N*^1^-propargylated dI analogue **29**, which was deprotected with ammonium fluoride affording **10**. Only using 2D NMR, the undesired structure was unequivocally determined (see below). The propargyl moiety was selectively introduced at the O6-position [21] via reaction with BOP and Cs_2_CO_3_ followed by treatment of the BOP adduct with propargyl alcohol and additional Cs_2_CO_3_ affording **27** in 91%. The *O*^6^-propargylated (**7**) and N1-propargyl deoxyinosine (**10**) derivatives were distinguished based on 2D NMR (HMBC) via propargyl CH_2_ correlation with the C2 and C6 carbons. The CH_2_ protons of **10** proved strongly correlated with both C2 and C6 indicating propargylation at *N*^1^, whereas CH_2_ protons of **7** are strongly correlated only with C6 indicative for propargylation at *O*^6^-position. The ^1^H-NMR signal for NCH_2_ appeared at higher field than anticipated at 4.89 ppm, whereas signal for OCH_2_ appeared in the range low filed at 5.28 ppm. Finally, the propynylamino moiety was introduced at C6 following activation of **23** with TIBSCl, DMAP, and NEt_3_ in DCM at rt for 48 h, and nucleophilic aromatic substitution with propargyl amine, EtOH at 70–80 °C for 24 h to furnish **28** in 57%. Deprotection of **27**–**29** using NH_4_F in MeOH at 70–80 °C for 24 h afforded **7**, **8**, and **10** in 71%, 75% and 80%, respectively.

### 2.2. Biological Properties

Having obtained the desired propargylated 2′-deoxynucleosides, their cytotoxicity and potential antiviral effect was determined based on reduction of the yellow colored 3-(4,5-dimethylthiazol-2-yl)-2,5-diphenyltetrazolium bromide (MTT) by mitochondrial dehydrogenase of metabolically active MT-4 cells to a blue formazan derivative [22]. In contrast to EdU, which shows quite cytotoxic, none of the various congeners displayed any remarkable toxic effect nor HIV inhibition (Table 1), paving the way for further evaluation of their selectivity for visualization of viral replication through click chemistry. The use of the MTT assay is appropriate as it is a standard assay to test for cell toxicity and HIV-1 inhibition after multiple rounds of replication (day 5). We also tested the effect on HIV-1 infection in single round (HIV-1 fLuc readout). Also here only EdU showed a major reduction of HIV-1 infection (not shown). The selectivity was examined in vitro with a primer extension assay comparing incorporation of the analogues by human DNA polymerase α, human mitochondrial DNA polymerase γ and HIV-1 reverse transcriptase and will be communicated elsewhere (De Wit et al., unpublished data).

**Figure 2:**
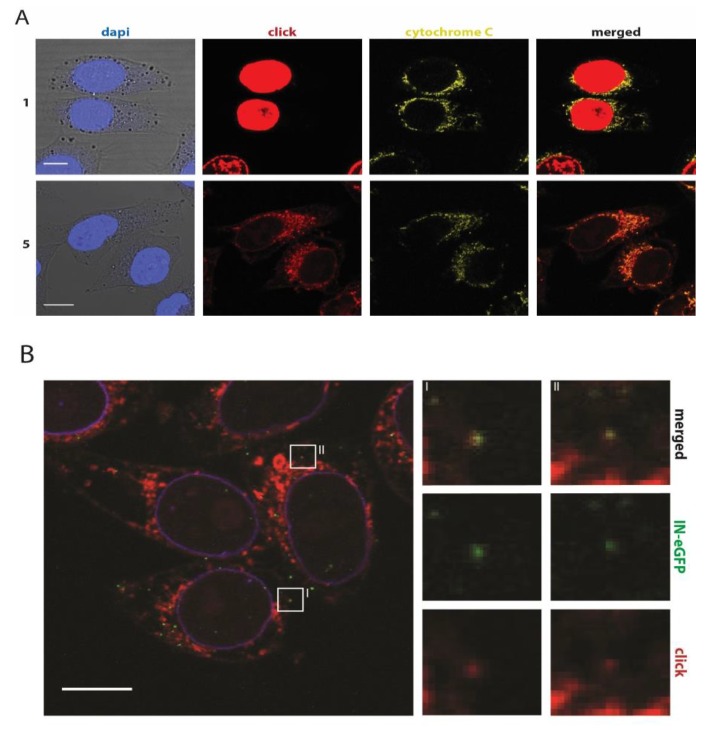
Localization of the click signal for compound **5**, showing predominantly incorporation into mitochondrial DNA (panel **A**) and co-localization with the HIV-integrase protein (panel **B**).

For preliminary visualization experiments, cells were treated with the newly synthesized propargylated analogues or EdU (**1**) as a control, followed by staining for cytochrome C (mitochondrial staining) together with the click reaction using the Click-iT EdU Alexa Fluor 647 imaging kit (Thermo Fisher Scientific Inc., Waltham, MA, USA). Visualization was done using confocal microscopy (Figure 2A at top for EdU, at bottom for **5**). In parallel, DAPI (4′,6-diamidino-2-phenylindole, binding to AT-rich regions) was used for nuclear staining. Remarkably, analogue **5** resulted in a clear, almost selective mitochondrial staining, as shown by co-localization with cytochrome C (Figure 2A, bottom, panel D). No nuclear staining is seen (absence of fluorescence in the DAPI stained region) in contrast with EdU.

In another experiment, using IN-eGFP [11], co-localization of both fluorescence signals resulting from the click reaction and the HIV-integrase protein, respectively, will indicate the selectivity of the various compounds for cDNA labeling. As shown in Figure 2B, the same propargylated analogue **5** afforded co-localization signals with the IN-eGFP virus, but analysis was hampered as of the ubiquitous mitochondrial staining. Full biological evaluation of all analogues will be reported elsewhere.

## 3. Materials and Methods

All ^1^H and ^13^C-NMR spectra and MS analytical spectra are available online, see Supplementary Materials.

### 3.1. Chemistry

#### 3.1.1. 8-Bromo-3′,5′-*O*-bis(tert-butyldimethylsilyl)-2′deoxyadenosine (**14**)

To a stirred solution of 8-bromo-2′-deoxyadenosine **13** (0.300 g, 0.909 mmol) in dry DMF (15 mL) was added imidazole (0.371 g, 5.45 mmol), dimethylaminopyridine (30 mg, 0.25 mmol) and TBDMSCl (0.411 g, 2.72 mmol) at rt under N_2_ atm. After stirring for 24 h, water (30 mL) and EtOAc (30 mL) were added. The aqueous phase was extracted with EtOAc (3 × 30 mL), and the combined organic phases were dried (Na_2_SO_4_), filtered, and the solvent was removed in vacuo. The residue was purified by column chromatography on silica gel (hexane:EtOAc = 6:4) and afforded **14** (0.412 g, 81%) as a white solid. ^1^H-NMR (300 MHz, CDCl_3_) δ 8.27 (s, 1H, H2), 6.37 (t, *J* = 6.7 Hz, 1H, H1′), 5.69 (brs, 2H, NH_2_), 4.89 (dd, *J* = 9.4, 3.8 Hz, 1H, H3′), 4.03–3.87 (m, 2H, H4′, H5′a), 3.75–3.62 (m, 2H, H5′b, H2′a), 2.25 (ddd, *J* = 13.1, 7.0, 4.3 Hz, 1H, H2′b), 0.96 (s, 9H, SiC(CH_3_)_3_), 0.85 (s, 9H, SiC(CH_3_)_3_), 0.16 (s, 6H, Si(CH_3_)_2_), 0.02 (s, 3H, SiCH_3_), −0.02 (s, 3H, SiCH_3_); ^13^C-NMR (75 MHz, CDCl_3_) δ 153.9, 152.2, 150.6, 127.9, 120.2, 87.4, 86.0, 72.0, 62.3, 36.4, 25.5, 18.0, 17.7, −5.0, −5.0, −5.8, −5.8; HR-ESI MS (*m*/*z*): [M + H]^+^ calculated for C_22_H_41_BrN_5_O_3_Si_2_, 558.1926; found 558.1929 [23].

#### 3.1.2. 3′,5′-*O*-Bis(tert-butyldimethylsilyl)-8-propynyloxy-2′deoxyadenosine (**15**)

Compound **14** (0.300 g, 0.537 mmol) was dissolved in anhydrous THF (15 mL) in a round bottom flask under Ar atm. In a separate reaction flask fitted with balloon, propargyl alcohol (0.53 mL, 9.14 mmol) was added to freshly distilled THF (10 mL) and the solution was kept under N_2_ atm and cooled down to −40 °C. Then, *n*-BuLi (2.5 mL of a 2.5 M hexane solution, 6.44 mmol) was gradually added to the latter reaction mixture; the by-product butane was collected in the balloon. The reaction was completed instantly. This in situ generated lithium propargyloxide was transferred to the flask containing **14** through an oven-dried syringe. Progress of the reaction at rt was monitored by TLC. After 24 h, the solution was neutralized by dilute acetic acid. Excess solvent was removed under reduced pressure and the solid residue was then partitioned between water (50 mL) and EtOAc (50 mL). The layers were separated and the aqueous layer was extracted with EtOAc (3 × 50 mL). The combined organic layers were washed with brine (50 mL), dried (Na_2_SO_4_) and concentrated in vacuo and purified by flash column chromatography (hexane:EtOAc = 10:1) affording **15** (0.260 g, 91%) as a brown semisolid. ^1^H-NMR (300 MHz, CDCl_3_) δ 8.21 (s, 1H, H2), 6.35 (t, *J* = 6.9 Hz, 1H, H1′), 5.76 (d, *J* = 4.4 Hz, 2H, NH_2_), 5.11 (d, *J* = 2.5 Hz, 2H, OCH_2_), 4.73 (dt, *J* = 6.4, 3.5 Hz, 1H, H3′), 3.93 (ddd, *J* = 6.9, 4.9, 3.2 Hz, 1H, H4′), 3.84 (dd, *J* = 10.6, 6.9 Hz, 1H, H5′a), 3.69 (dd, *J* = 10.6, 5.0 Hz, 1H, H5′b), 3.31 (dt, *J* = 13.1, 6.5 Hz, 1H, H2′a), 2.61 (t, *J* = 2.4 Hz, 1H, CH), 2.18 (ddd, *J* = 13.1, 6.9, 3.7 Hz, 1H, H2′b), 0.93 (s, 9H, SiC(CH_3_)_3_), 0.86 (s, 9H, SiC(CH_3_)_3_), 0.13 (s, 6H, Si(CH_3_)_2_), 0.01 (d, *J* = 6.2 Hz, 6H, Si(CH_3_)_2_); ^13^C-NMR (75 MHz, CDCl_3_) δ 153.4, 153.1, 150.8, 149.6, 115.3, 87.1, 82.2, 76.7, 76.1, 72.3, 62.8, 57.2, 36.4, 25.6, 25.5, 18.0, 17.7, −5.0, −5.1, −5.7, −5.8; HR-ESI MS (*m*/*z*): [M + H]^+^ calculated for C_25_H_44_N_5_O_4_Si_2_, 534.2926; found 534.2931.

#### 3.1.3. 8-Propynyloxy-2′-deoxyadenosine (**4**)

To a stirred solution of **15** (0.250 g, 0.47 mmol) in anh.THF (10 mL) was added TBAF (1M in THF) (1.87 mL, 1.87 mmol) at rt under N_2_ atm. After reaction for 3 h, the solvent was removed in vacuo, and the residue was purified by flash column chromatography on silica gel (MeOH:DCM = 1:9) to give the title compound **4** (0.121 g, 85%) as a white solid. ^1^H-NMR (300 MHz, MeOD) δ 8.06 (s, 1H, H-2), 6.36 (dd, *J* = 8.5, 6.3 Hz, 1H, H1′), 5.19 (d, *J* = 2.4 Hz, 2H, OCH_2_), 4.63–4.58 (m, 1H, H3′), 4.06 (dd, *J* = 5.1, 3.0 Hz, 1H, H4′), 3.91–3.70 (m, 2H, H5′), 3.17 (t, *J* = 2.4 Hz, 1H, CH), 2.98 (ddd, *J* = 14.3, 8.5, 5.8 Hz, 1H, H2′a), 2.27 (ddd, *J* = 13.4, 6.3, 2.0 Hz, 1H, H2′b); ^13^C-NMR (75 MHz, MeOD) δ 153.7, 152.7, 149.9, 148.0, 114.9, 88.1, 83.6, 76.5, 76.3, 71.8, 62.5, 57.3, 37.9; UV λ_max_ (nm): 260; HR-ESI MS (*m*/*z*): [M + H]^+^ calculated for C_13_H_16_N_5_O_4_, 306.1196; found 306.1196.

#### 3.1.4. 8-Propynylamino-2′-deoxyadenosine (**3**)

To a stirred solution of 8-bromoadenosine **13** (0.250 g, 0.76 mmol) in absolute ethanol (20 mL) was added calcium carbonate (0.151 g, 1.52 mmol) and propargylamine (0.970 mL, 15.15 mmol) at rt under N_2_ atm. The reaction mixture was slowly heated at 70–80 °C for 14 h, then the reaction mixture was filtered to remove the calcium salts, and the filtrate was concentrated by rotary evaporation. The residue was purified by column chromatography on silica gel (MeOH:DCM = 1:9) affording **3** (0.170 g, 72.6%) as a white solid. ^1^H-NMR (300 MHz, MeOD) δ 8.01 (s, 1H, H2), 6.46 (dd, *J* = 9.3, 5.8 Hz, 1H, H1′), 4.59 (dt, *J* = 6.1, 1.9 Hz, 1H, H3′), 4.17 (dd, *J* = 2.5, 1.5 Hz, 2H, NHCH_2_), 4.07 (q, *J* = 2.1 Hz, 1H, H4′), 3.95–3.78 (m, 2H, H5′), 2.74 (ddd, *J* = 13.4, 9.3, 6.1 Hz, 1H, H2′a), 2.63 (t, *J* = 2.4 Hz, 1H, CH), 2.19 (ddd, *J* = 13.4, 5.9, 1.7 Hz, 1H, H2′b); ^13^C-NMR (75 MHz, MeOD) δ 151.9, 150.8, 148.8, 148.5, 116.1, 87.5, 83.6, 79.5, 71.6, 70.6, 61.4, 37.9, 30.9; UV λ_max_ (nm): 276; HR-ESI MS (*m*/*z*): [M + H]^+^ calculated for C_13_H_17_N_6_O_3_, 305.1356; found 305.1361.

#### 3.1.5. 8-Bromo-2′-deoxyguanosine (**17**)

To a stirred solution of **16** (3.0 g, 11.23 mmol) in MeCN (120 mL) and H_2_O (30 mL) was added *N*-bromosuccinimide (NBS; 3.0 g, 16.85 mmol) in three portions. The suspension was stirred for 45 min at rt, and subsequently evaporated to dryness. The residual solid was filtered. The solid was washed with cold acetone (100 mL) and dried in vacuo to give the title compound **17** (3.53 g, 91%) as an orange solid. ^1^H-NMR (300 MHz, DMSO-*d*_6_) δ 10.80 (s, 1H, NH), 6.16 (t, *J* = 7.3 Hz, 1H, H1′), 4.40 (dt, *J* = 6.1, 3.0 Hz, 1H, H3′), 3.81 (td, *J* = 5.5, 2.9 Hz, 1H, H4′), 3.63 (dd, *J* = 11.7, 5.4 Hz, 1H, H5′a), 3.50 (dd, *J* = 11.6, 5.9 Hz, 1H, H5′b), 3.17 (dt, *J* = 13.8, 7.1 Hz, 1H, H2′a), 2.11 (ddd, *J* = 13.7, 7.0, 2.8 Hz, 1H, H2′b); ^13^C-NMR (75 MHz, DMSO) δ 155.6, 153.5, 152.1, 120.7, 117.6, 88.0, 85.2, 71.2, 62.2, 36.6; HR-ESI MS (*m*/*z*): [M + H]^+^ calculated for C_10_H_13_BrN_5_O_4_, 346.0145; found, 346.0145 [24].

#### 3.1.6. 8-Bromo-3′,5′-*O*-bis(tert-butyldimethylsilyl)-2′-deoxyguanosine (**18**)

To a stirred solution of **17** (3 g, 8.67 mmol) in dry DMF (60 mL) was added imidazole (3.54 g, 52.0 mmol) and tert butyl(chloro)dimethylsilane (3.92 g, 26.0 mmol) at rt under N_2_ atm. After 15 h, solvents were removed in vacuo. The reaction mixture was diluted with EtOAc (60 mL), quenched with sat aq. NaHCO_3_ solution (30 mL), and diluted with H_2_O (30 mL). The aqueous phase was extracted with EtOAc (3 × 60 mL), and the combined organic phases were dried (Na_2_SO_4_), filtered, and the solvent was removed in vacuo. The residue was purified by column chromatography on silica gel (MeOH:DCM = 6:94) to give the title compound **18** (4.3 g, 87%) as a pale yellow solid; ^1^H-NMR (300 MHz, CDCl_3_) δ 11.90 (brs, 1H, NH), 6.48 (brs, 2H, NH_2_), 6.24 (t, *J* = 6.9 Hz, 1H, H1′), 4.75 (dt, *J* = 6.5, 3.6 Hz, 1H, H3′), 3.90 (m, 2H, H4′, H5′a), 3.73 (dd, *J* = 10.2, 5.1 Hz, 1H, H5′b), 3.50 (dt, *J* = 12.8, 6.4 Hz, 1H, H2′a), 2.18 (ddd, *J* = 12.8, 6.7, 3.9 Hz, 1H, H2′b). 0.96 (s, 9H, Si(CH_3_)_3_), 0.88 (s, 9H, Si(CH_3_)_3_), 0.16 (s, 6H, Si(CH_3_)_2_), 0.05 (s, 3H, Si(CH_3_)), 0.03 (s, 3H, Si(CH_3_); ^13^C-NMR (75 MHz, CDCl_3_) δ 157.6, 152.8, 152.3, 122.0, 117.6, 87.3, 85.5, 72.1, 62.5, 36.3, 25.6, 25.5, 18.0, 17.7, −4.9, −4.9, −5.7, −5.7; HR-ESI MS (*m*/*z*): [M + H]^+^ calculated for C_22_H_41_BrN_5_O_4_Si_2_, 574.1875; found 574.1888 [16].

#### 3.1.7. 8-Bromo-3′,5′-*O*-bis(tert-butyldimethylsilyl)-6-*O*-(trimethylsilylethyl)-2′-deoxyguanosine (**19**)

Compound **18** (4.00 g, 6.96 mmol), triphenylphosphine (2.74 g, 10.45 mmol) and 2-(trimethylsilyl)ethanol (1.51 mL, 10.45 mmol) were mixed in anhydrous dioxane (80 mL). The mixture was stirred at rt for 10 min, and then cooled down to 0 °C. Diisopropyl azodicarboxylate (DIAD) (2.05 mL, 10.45 mmol) was added and the mixture was heated at 40 °C for 24 h. The resulting mixture was concentrated in vacuo, and the residue was diluted with EtOAc (100 mL) and water (100 mL). The layers were separated and the aqueous layer was extracted with EtOAc (3 × 100 mL). The combined organic layers were washed with brine (50 mL), dried (Na_2_SO_4_) and the solvent was removed in vacuo. The residue was purified by column chromatography on silica gel (EtOAc:hexane = 1:9) to give the title compound **19** (3.05 g, 65%) as colorless oil; ^1^H-NMR (300 MHz, CDCl_3_) δ 6.29 (t, *J* = 6.8 Hz, 1H, H1′), 4.83–4.77 (m, 1H, H3′), 4.75 (brs, 2H, NH_2_), 4.63–4.44 (m, 2H, OCH_2_), 3.98–3.83 (m, 2H, H4′, H5′a), 3.71 (dd, *J* = 10.2, 4.3 Hz, 1H, H5′b), 3.56 (dt, *J* = 13.0, 6.4 Hz, 1H, H2′a), 2.17 (ddd, *J* = 13.0, 6.8, 4.0 Hz, 1H, H2′b), 1.26–1.18 (m, 2H, CH_2_Si), 0.95 (s, 9H, SiC(CH_3_)_3_), 0.86 (s, 9H, SiC(CH_3_)_3_), 0.15 (s, 6H, Si(CH_3_)_2_), 0.09 (s, 9H, SiC(CH_3_)_3_), 0.03 (s, 3H, SiCH_3_), −0.01 (s, 3H, SiCH_3_); ^13^C-NMR (75 MHz, CDCl_3_) δ 159.9, 158.3, 153.9, 125.3, 116.3, 87.1, 85.4, 72.1, 64.7, 62.4, 36.1, 25.6, 25.5, 18.0, 17.7, 17.2, −1.8, −4.9, −5.0, −5.7, −5.8; HR-ESI MS (*m*/*z*): [M + H]^+^ calculated for C_27_H_53_BrN_5_O_4_Si_3_, 674.2583; found 674.2590 [16].

#### 3.1.8. 8-Bromo-3′,5′-*O*-bis(tert-butyldimethylsilyl)-2-*N*-isobutyryl-6-*O*-(trimethylsilylethyl)-2′-deoxyguanosine (**20**)

To a stirred solution of **19** (0.900 g, 1.2 mmol) in dry pyridine (10 mL) was added isobutyrylchloride (0.25 mL, 2.4 mmol) at 0 °C under Ar gas and the mixture was stirred for 3 h at rt. The reaction mixture was quenched with saturated aqueous NaHCO_3_ (25 mL) and extracted with DCM (3 × 50 mL). The combined organic layers were washed with brine (50 mL), dried (Na_2_SO_4_), filtered, and the solvent was removed in vacuo. The residue was purified by flash column chromatography on silica gel (EtOAc:hexane = 1:9) to give the title compound **20** (0.845 g, 85%) as colorless oil. ^1^H-NMR (300 MHz, CDCl_3_) δ 7.71 (s, 1H, NH), 6.36 (t, *J* = 7.0 Hz, 1H, H1′), 4.85 (dt, *J* = 6.1, 3.2 Hz, 1H, H3′), 4.66–4.50 (m, 2H, OCH_2_), 4.02–3.84 (m, 2H, H4′, H5′a), 3.74 (dd, *J* = 10.6, 5.2 Hz, 1H, H5′b), 3.59 (dt, *J* = 13.3, 6.5 Hz, 1H, H2′a), 3.10 (brs, 1H, COCH), 2.21 (ddd, *J* = 13.1, 6.6, 3.5 Hz, 1H, H2′b), 1.34–1.20 (m, 8H, C(CH_3_)2, CH_2_Si), 0.94 (s, 9H, SiC(CH_3_)_3_), 0.86 (s, 9H, SiC(CH_3_)_3_), 0.17 (s, 3H, SiCH_3_), 0.15 (s, 3H, SiCH_3_), 0.11 (s, 9H, SiC(CH_3_)_3_), 0.02 (s, 3H, SiCH_3_), 0.01 (s, 3H, SiCH_3_); ^13^C-NMR (75 MHz, CDCl_3_) δ 175.1, 159.6, 153.0, 151.0, 128.2, 118.7, 87.8, 85.8, 72.2, 65.6, 62.9, 36.5, 35.2, 25.6, 25.5, 19.0, 18.9, 18.0, 17.6, 17.1, −1.8, −5.0, −5.7. HR-ESI MS (*m*/*z*): [M + H]^+^ calculated for C_31_H_59_Br_1_N_5_O_5_Si_3_, 744.3002; found 744.3018.

#### 3.1.9. 3′,5′-*O*-Bis(tert-butyldimethylsilyl)-2-*N*-isobutyryl-8-propynylamino-6-*O*-(trimethyl-silylethyl)-2′-deoxyguanosine (**21**)

To a stirred suspension of **20** (0.400 g, 0.54 mmol) in anhydrous, degassed 1.4 dioxane (15 mL) was added propargylamine (0.051 mL, 0.536 mmol), Pd_2_(dba)_3_ (50 mg, 0.536 mmol), BINAP (100 mg, 0.536 mmol) and cesium carbonate (0.262 g, 0.804 mmol) at rt under N_2_ atm. The reaction was slowly heated at 100 °C for 48 h, then cooled to rt and quenched with saturated aq. NaHCO_3_ (15 mL). The aqueous phase was extracted with EtOAc (3 × 30 mL), and the combined organic phases were dried (Na_2_SO_4_), filtered, and the solvent was removed in vacuo. The residue was purified by column chromatography on silica gel (EtOAc:hexane = 15:85) to give the title compound **21** (0.235 g, 61%) as a colorless oil. ^1^H-NMR (300 MHz, CDCl_3_) δ 7.70 (s, 1H, NH), 6.35 (dd, *J* = 8.3, 5.9 Hz, 1H, H1′), 5.82 (t, *J* = 5.9 Hz, 1H, NH), 4.77–4.44 (m, 3H, H3′, OCH_2_), 4.42–4.19 (m, 2H, NHCH_2_), 4.12–3.73 (m, 3H, H4′, H5′), 3.51 (brs, 1H, COCH), 2.64 (dt, *J* = 14.1, 7.2 Hz, 1H, H2′a), 2.36–2.06 (m, 2H, CH, H2′b), 1.56–1.15 (m, 8H, HC(CH_3_)_2_, OCH_2_Si), 0.93 (s, 18H, 2 × SiC(CH_3_)_3_), 0.41–0.00 (m, 21H, SiC(CH_3_)_3_, 2 × Si(CH_3_)_2_); ^13^C-NMR (75 MHz, CDCl_3_) δ 176.9, 157.2, 152.7, 151.5, 148.8, 115.2, 86.9, 83.4, 80.3, 71.4, 71.0, 64.8, 62.4, 38.9, 32.2, 25.7, 25.4, 18.9, 18.3, 17.6, 17.5, −1.8, −4.9, −5.1, −5.5, −5.7; HR-ESI MS (*m*/*z*): [M + H]^+^ calculated for C_34_H_63_N_6_O_5_Si_3_, 719.4162 ; found 719.4179.

#### 3.1.10. 8-Propynylamino-2′-deoxyguanosine (**9**)

To a stirred solution of **21** (0.230 g, 0.32 mmol) in THF (10 mL) was added TBAF (1 M in THF) (1.6 mL, 1.6 mmol) at rt under N_2_ atm. After 4 h the solvent was removed under reduced pressure and the crude mixture was purified by flash chromatography on silica, eluting with 20% MeOH in DCM to afford the intermediate. Without further purification, the crude mixture was dissolved in a glass sealed tube was added 7*N* NH_3_ in MeOH (15 mL) at rt. The reaction was slowly heated to 60 °C for 14 h, then cooled to rt, concentrated in vacuo and purified by flash column chromatography to give compound **9** along with TBAF impurities. Then to a stirred solution of the crude mixture in water (10 mL) was added NH_4_PF_6_ (150 mg) at rt for 30 min. The mixture was washed with DCM (3 × 10 mL) and water was removed in vacuo. The residue was purified by column chromatography on silica gel (MeOH:DCM = 1:3 containing 1% aq.NH_3_) and afforded the alkyn **9** (66 mg, 65% overall for both steps) as a colorless oil. ^1^H-NMR (300 MHz, MeOD) δ 6.34 (dd, *J* = 9.1, 6.0 Hz, 1H, H1′), 4.57 (dt, *J* = 6.5, 2.2 Hz, 1H, H3′), 4.18–4.04 (m, 2H, NHCH_2_), 4.00 (q, *J* = 2.5 Hz, 1H, H4′), 3.95–3.69 (m, 2H, H5′a), 2.73 (ddd, *J* = 13.1, 9.2, 6.4 Hz, 1H, H2′a), 2.55 (t, *J* = 2.5 Hz, 1H, CH), 2.12 (ddd, *J* = 13.4, 5.9, 1.9 Hz, 1H, H2′b).^13^C-NMR (75 MHz, MeOD) δ 156.4, 152.2, 150.7, 148.8, 111.9, 87.1, 83.1, 79.9, 71.4, 70.3, 61.1, 37.6, 31.1; UV λ_max_ (nm): 260 (br); HR-ESI MS (*m*/*z*): [M + H]^+^ calculated for C_13_H_17_N_6_O_4_, 321.1305; found 321.1303.

#### 3.1.11. 3′,5′-*O*-Bis(tert-butyldimethylsilyl)-2′-deoxyguanosine (**22**)

To a stirred solution of 2′-deoxyguanosine **16** (1.2 g, 4.49 mmol) in dry DMF (60 mL) was added imidazole (1.83 g, 26.96 mmol) and TBDMSCl (2.032 g, 13.48 mmol) at rt under N_2_ atm. After 24 h, DMF was evaporated in vacuo and the residue was partitioned between water (60 mL) and EtOAc (60 mL) were added (60 mL). The aqueous phase was extracted with EtOAc (3 × 60 mL), and the combined organic phases were dried (Na_2_SO_4_), filtered, and the solvent was removed in vacuo. The residue was purified by column chromatography on silica gel (MeOH:DCM = 5:95) affording **22** (1.86 g, 84%) as a white solid. ^1^H-NMR (300 MHz, DMSO-*d*_6_) δ 10.62 (s, 1H, NH), 7.89 (s, 1H, H8), 6.48 (s, 2H, NH_2_), 6.11 (dd, *J* = 7.7, 6.0 Hz, 1H, H1′), 4.52–4.44 (m, 1H, H3′), 3.81 (dd, *J* = 5.5, 2.8 Hz, 1H, H4′), 3.75–3.61 (m, 2H, H5′), 2.72–2.58 (m, 1H, H2′a), 2.24 (ddd, *J* = 13.2, 6.0, 3.2 Hz, 1H, H2′b), 0.89 (d, *J* = 5.1 Hz, 18 H, 2 × SiC(CH_3_)_3_), 0.11 (s, 6H, SiC(CH_3_)_2_), 0.05 (d, *J* = 1.5 Hz, 6H, SiC(CH_3_)_2_); ^13^C-NMR (75 MHz, DMSO) δ 156.8, 153.9, 151.1, 135.0, 116.8, 87.1, 82.3, 72.3, 62.9, 25.9, 25.8, 18.1, 17.8, −4.6, −4.8, −5.3, −5.4; HR-ESI MS (*m*/*z*): [M + H]^+^ calculated for C_22_H_42_N_5_O_4_Si_2_, 496.2766; found 496.2769 [25].

#### 3.1.12. 3′,5′-*O*-Bis(tert-butyldimethylsilyl)-6-*O*-propynyl-2′-deoxyguanosine (**23**)

To a stirred solution of **22** (0.500 g, 1.01 mmol) in dry 1,4 dioxane (40 mL) was added triphenyl phosphine (0.317 g, 1.21 mmol) at rt under N_2_ atm. The reaction mixture was slowly heated at 60 °C for 1 h, propargylalcohol (0.087 mL, 1.51 mmol) was added and after stirring for 30 min, DIAD (0.300 mL, 1.51 mmol) was added and the mixture was further stirred overnight at 60 °C. The crude reaction mixture was concentrated in vacuo, and the mixture was purified by silica gel column chromatography (hexane:EtOAc = 6:4) to give **23** (0.266 mg, 50%) as a white solid: ^1^H-NMR (300 MHz, CDCl_3_) δ 7.93 (s, 1H, H8), 6.31 (t, *J* = 6.5 Hz, 1H, H1′), 5.10 (dd, *J* = 2.4, 0.8 Hz, 2H, OCH_2_), 5.07 (brs, 2H, NH_2_), 4.58 (dt, *J* = 5.6, 3.5 Hz, 1H, H3′), 3.97 (q, *J* = 3.5 Hz, 1H, H4′), 3.78 (qd, *J* = 11.2, 3.5 Hz, 2H, H5′), 2.61–2.49 (m, 1H, H2′a), 2.46 (t, *J* = 2.4 Hz, 1H, CH), 2.40–2.32 (m, 1H, H2′b), 0.90 (s, 18H, 2x SiC(CH_3_)_3_), 0.09 (s, 6H, SiC(CH_3_)_2_), 0.07 (s, 6H, SiC(CH_3_)_2_); ^13^C-NMR (75 MHz, CDCl_3_) δ 159.5, 158.7, 153.5, 137.5, 115.4, 87.3, 83.3, 78.06, 74.5, 71.5, 62.4, 53.4, 40.6, 25.6, 25.4, 18.1, 17.7, −5.0, −5.1, −5.7, −5.8; HR-ESI MS (*m*/*z*): [M + H]^+^ calculated for C_25_H_44_N_5_O_4_Si_2_, 534.2926; found 534.2930.

#### 3.1.13. 3′,5′-*O*-Bis(tert-butyldimethylsilyl)-6-*N*-propynyl-2,6-diaminopurine-2′-deoxyriboside (**24**)

To a stirred solution of **22** (0.300 g, 0.606 mmol) in dry DCM (50 mL) was added Et_3_N (0.25 mL, 1.82 mmol), TIBSCl (0.55 g, 1.82 mmol) and DMAP (9 mg, 0.08 mmol) at rt. After stirring for 48 h, the reaction mixture was concentrated in vacuo and the residue was partitioned between EtOAc (30 mL) and water (30 mL). The aq. layer was again extracted with EtOAc (3 × 30 mL). The combined organic layer was washed with water (50 mL) and brine (50 mL), dried over Na_2_SO_4_, filtered and evaporated under vacuum to get crude compound. The crude mixture was dissolved in a glass sealed tube 30 mL of EtOH and propargylamine (0.776 mL, 12.12 mmol) and DIPEA (0.526 mL, 3.03 mmol) were added at rt. The reaction mixture was slowly heated to 100 °C for 14 h. The reaction was quenched by saturated aqueous NaHCO_3_, and EtOH was removed under reduced pressure. The aqueous layer was extracted with EtOAc (3 × 50 mL). The organic layer was washed with water (50 mL) and brine (50 mL), dried over Na_2_SO_4_, and evaporated under vacuum. The residue was purified by flash silica gel chromatography (hexane:EtOAc = 6:4) to give compound **24** (0.178 g, 55%) as a white solid. ^1^H-NMR (300 MHz, CDCl_3_) δ 7.80 (s, 1H, H8), 6.31 (t, *J* = 6.5 Hz, 1H, H1′), 5.83 (t, *J* = 5.1 Hz, 1H, NH), 4.77 (s, 2H, NH_2_), 4.60 (dt, *J* = 6.0, 3.2 Hz, 1H, H3′), 4.40 (d, *J* = 2.7 Hz, 2H, NHCH_2_), 3.98 (dd, *J* = 6.6, 3.3 Hz, 1H, H4′), 3.80 (qd, *J* = 11.2, 3.9 Hz, 2H, H5′), 2.60 (dt, *J* = 12.9, 6.4 Hz, 1H, H2′a), 2.42–2.30 (m, 1H, H2′b), 2.25 (t, *J* = 2.4 Hz, 1H, CH), 0.93 (s, 18H, 2x SiC(CH_3_)_3_), 0.11 (s, 6H, SiC(CH_3_)_2_), 0.10 (s, 6H, SiC(CH_3_)_2_); ^13^C-NMR (75 MHz, CDCl_3_) δ 159.4, 154.0, 150.9, 135.6, 114.7, 87.3, 83.2, 80.0, 71.7, 71.0, 62.5, 40.4, 29.9, 25.6, 25.4, 18.1, 17.7, −4.9, −5.1, −5.7, −5.8; HR-ESI MS (*m*/*z*): [M + H]^+^ calculated for C_25_H_45_N_6_O_3_Si_2_, 533.3086; found 533.3088.

#### 3.1.14. 6-*O*-Propynyl-2′-deoxyguanosine (**5**)

To a stirred solution of compound **23** (0.240 g, 0.45 mmol) in THF (15 mL) was added TBAF (1 M in THF) (1.80 mL, 1.80 mmol) at rt. After 2 h, the solvent was removed in vacuo, and the residue was purified by flash column chromatography on silica gel (MeOH:DCM = 1:9) to give the title compound **5** (116 mg, 85%) as a white solid. ^1^H-NMR (300 MHz, MeOD) δ 8.06 (s, 1H, H8), 6.32 (dd, *J* = 7.8, 6.1 Hz, 1H, H1′), 5.13 (d, *J* = 2.4 Hz, 2H, OCH_2_), 4.58 (dd, *J* = 5.5, 2.7 Hz, 1H, H3′), 4.06 (dd, *J* = 5.6, 2.9 Hz, 1H, H4′), 3.91–3.70 (m, 2H, H5′), 2.96 (t, *J* = 2.4 Hz, 1H, CH), 2.79 (ddd, *J* = 13.7, 7.8, 6.1 Hz, 1H, H2′a), 2.37 (ddd, *J* = 13.4, 6.1, 2.7 Hz, 1H, H2′b); ^13^C-NMR (75 MHz, MeOD) δ 159.5, 159.4, 152.8, 138.6, 114.1, 87.9, 84.9, 77.6, 74.9, 71.4, 61.9, 53.0, 39.4; UV λ_max_ (nm): 210, 245 (br), 280 (br); HR-ESI MS (*m*/*z*): [M + H]^+^ calculated for C_13_H_16_N_5_O_4_, 306.1196; found 306.1197 [12].

#### 3.1.15. 6-*N*-Propynyl-2,6-diaminopurine-2′-deoxyriboside (**6**)

To a stirred solution of **24** (0.160 g, 0.300 mmol) in MeOH (20 mL) was added NH_4_F (0.110 g, 3 mmol) at rt. The reaction mixture was slowly heated to 60–70 °C for 24 h. The solvent was removed in vacuo, and the residue was purified by flash column chromatography on silica gel (MeOH:DCM = 1:9) to give compound **6** (74 mg, 81%) as a white solid. ^1^H-NMR (300 MHz, MeOD) δ 7.90 (s, 1H, H8), 6.28 (dd, *J* = 8.4, 5.9 Hz, 1H, H1′), 4.57 (dt, *J* = 4.1, 1.8 Hz, 1H, H3′), 4.34 (s, 2H, NHCH_2_), 4.07 (dd, *J* = 4.8, 2.6 Hz, 1H, H4′), 3.81 (ddd, *J* = 35.9, 12.3, 2.8 Hz, 2H, H5′), 2.81 (ddd, *J* = 14.0, 8.5, 5.9 Hz, 1H, H2′a), 2.61 (t, *J* = 2.5 Hz, 1H, CH), 2.33 (ddd, *J* = 13.4, 5.9, 2.2 Hz, 1H, H2′b); ^13^C-NMR (75 MHz, MeOD) δ 159.7, 154.2, 149.7, 136.6, 113.8, 88.1, 85.4, 79.7, 71.7, 70.4, 62.2, 39.4, 28.9; UV λ_max_ (nm): 259, 280 (br); HR-ESI MS (*m*/*z*): [M + H]^+^ calculated for C_13_H_17_N_6_O_3_, 305.1356; found 305.1356.

#### 3.1.16. 3′,5′-*O*-Bis(tert-butyldimethylsilyl)-2′-deoxyinosine (**26**)

To a stirred solution of 2′-deoxyinosine **25** (2.0 g, 7.93 mmol) in dry DMF was added imidazole (3.77 g, 55.5 mmol) and tert-butylchlorodimethysilane (4.76 g, 31.7 mmol) at rt under N_2_ atm. The resulting yellow solution was stirred at rt for 24 h. Ethanol (20 mL) was added and the solution was stirred for an additional 15 min at rt. After the solvent was evaporated, the residue was dissolved in DCM (100 mL), washed consecutively with aq. HCl (1 M), sat. aq. Na_2_CO_3_ and brine and the organic phase was dried with anh. Na_2_SO_4_. The solvent was evaporated in vacuo to get yield **26** (3.51 g, 92%) as a white solid. ^1^H-NMR (300 MHz, CDCl_3_) δ 8.22 (s, 1H, H8), 8.18 (s, 1H, H2), 6.43 (t, *J* = 6.3 Hz, 1H, H1′), 4.63 (dt, *J* = 5.6, 3.8 Hz, 1H, H3′), 4.04 (dd, *J* = 6.9, 3.4 Hz, 1H, H4′), 3.83 (qd, *J* = 11.2, 3.6 Hz, 2H, H5′), 2.66–2.37 (m, 2H, H2′a), 0.93 (s, 18H, 2x SiC(CH_3_)_3_), 0.12 (s, 6H, SiC(CH_3_)_2_), 0.11 (s, 6H, SiC(CH_3_)_2_); ^13^C-NMR (75 MHz, CDCl_3_) δ 158.9, 148.2, 144.6, 138.2, 124.7, 87.7, 84.2, 77.1, 76.7, 76.3, 71.4, 62.3, 41.3, 25.6, 25.4, 18.1, 17.7, −5.0, −5.1, −5.7, −5.8; HR-ESI MS (*m*/*z*): [M + H]^+^ calculated for C_22_H_41_N_4_O_4_Si_2_, 481.2660; found 481.2665 [26].

#### 3.1.17. 6-*O*-Propynyl-3′,5′-*O*-bis(tert-butyldimethylsilyl)-2′-deoxyinosine (**27**)

To a solution of 3′,5′-*O*-bis(tert-butyldimethylsilyl)-2′-deoxyinosine (**26**) (700 mg, 1.46 mmol) in dry THF (10 mL) were added 2 molar equiv each of BOP and Cs_2_CO_3_ under a nitrogen atmosphere. The mixture was stirred at room temperature for 1 h until the complete formation of BOP adduct. The resulting mixture was evaporated under reduced pressure; again 2 molar equiv of Cs_2_CO_3_ and 30 molar equiv of propargyl alcohol were added and the reaction was stirred at room temperature for 5 h. Following completion, the reaction mixture was diluted with water (50 mL) and extracted with EtOAc (3 × 20 mL). The organic layer was dried over MgSO_4_ and concentrated under reduced pressure. The crude product was purified by column chromatography on silica gel using ethyl acetate and hexane to obtained the desired product (0.681 mg, 78%) as a white solid. ^1^H-NMR (300 MHz, CDCl_3_) δ 8.55 (s, 1H, H8), 8.31 (s, 1H, H2), 6.55 (t, *J* = 6.4 Hz, 1H, H1′), 5.25 (t, *J* = 2.8 Hz, 2H, OCH_2_), 4.65 (m, 1H, H3′), 4.03 (m, 1H, H4′), 3.89 (qd, *J* = 11.2, 3.6 Hz, 2H, H5′), 2.70–2.60 (m, 1H, H2′b), 2.40–2.51 (m, 2H, H2′a, CH), 0.93 (s, 18H, SiC(CH_3_)_3_), 0.12 (s, 12H, Si(CH_3_)_2_; ^13^C-NMR (75 MHz, CDCl_3_) δ 159.5, 152, 151.8, 141. 2, 122.1, 88.1, 84.7, 78.3, 75.2, 71.9, 62.8, 54.3, 41.7, 29.8, 26.1, 25.9, 18.5, 18.1, −4.6, −4.7, −5.3, −5.4; HR-ESI MS (*m*/*z*): [M + H]^+^ calculated for C_25_H_43_N_4_O_4_Si_2_, 519.2817; found 519.2831.

#### 3.1.18. 6-*O*-Propynyl-2′deoxyinosine (**7**)

To a stirred solution of the propynylated dI derivative **(27)** (0.600 g, 1.16 mmol) in MeOH (30 mL) was added NH_4_F (0.428 g, 11.6 mmol) at rt. The reaction mixture was slowly heated to 60–70 °C and stirred for 24 h. The solvent was removed in vacuo, and the residue was purified by flash column chromatography on silica gel (MeOH:DCM = 1:9) to give the title compound **7** (0.23 g, 71%) as a white solid. ^1^H-NMR (300 MHz, MeOD) δ 8.58 (s, 1H, H8), 8.53 (s, 1H, H2), 6.55 (t, *J* = 6.6 Hz, 1H, H1′), 5.28 (d, *J* = 2.4 Hz, 2H, OCH_2_), 4.62–4.55 (m, 1H, H3′), 4.08 (m, 1H, H4′), 3.8 (qd, *J* = 12.1, 3.8 Hz, 2H, H5′), 3.01 (t, *J* = 2.4 Hz, 1H, CH), 2.86 (m, 1H, H2′a), 2.49 (m, 1H, H2′b); ^13^C-NMR (75 MHz, MeOD) δ 160.8, 152.9, 143.9, 122.7, 89.9, 86.9, 78.9, 76.8, 72.8, 63.4, 55.4, 41.5; HR-ESI MS (*m*/*z*): [M + H]^+^ calculated for C_13_H_15_N_4_O_4_, 291.1087; found 291.1088.

#### 3.1.19. 6-*N*-Propynyl-3′,5′-*O*-bis(tert-butyldimethylsilyl)-2′-deoxyadenosine (**28**)

To a stirred solution of **26** (0.700 g, 1.46 mmol) in dry DCM (50 mL) was added Et_3_N (0.61 mL, 4.38 mmol), TIBSCl (1.325 g, 4.38 mmol) and DMAP (23 mg, 0.190 mmol) at rt under N_2_ atm. Following 48 h of reaction, the mixture was concentrated in vacuo and the residue was dissolved with EtOAc (50 mL) and water (50 mL). After separation of both layers, the aq. Layer was extracted with EtOAc (3 × 50 mL). The combined organic layer was washed with water (50 mL) and brine (50 mL), dried over Na_2_SO_4_, filtered and evaporated under vacuum to get crude compound. The crude mixture was dissolved in EtOH (50 mL) to which was added propargylamine (1.86 mL, 7.3 mmol) and DIPEA (1.267 mL, 29.16 mmol) in a glass sealed tube at rt. The reaction mixture was slowly heated to 100 °C for 14 h. After quenching with sat aq. NaHCO_3_, EtOH was removed under vacuum. The aqueous layer was extracted with EtOAc (3 × 50 mL). The organic layer was washed with water and brine, dried over Na_2_SO_4_, and evaporated under vacuum. The residue was purified by flash silica gel chromatography (hexane:EtOAc = 6:4) to give **28** (0.430 g, 57%) as a colorless oil. ^1^H-NMR (300 MHz, CDCl_3_) δ 8.45 (s, 1H, H8), 8.13 (s, 1H, H2), 6.46 (t, *J* = 6.4 Hz, 1H, H1′), 6.23 (t, *J* = 5.6 Hz, 1H, NH), 4.62 (dt, *J* = 5.6, 3.8 Hz, 1H, H3′), 4.49 (d, *J* = 2.5 Hz, 2H, NHCH_2_), 4.02 (dd, *J* = 7.1, 3.8 Hz, 1H, H4′), 3.83 (ddd, *J* = 14.4, 11.2, 3.7 Hz, 2H, H5′), 2.65 (dt, *J* = 12.8, 6.2 Hz, 1H, H2′a), 2.44 (ddd, *J* = 13.0, 6.1, 3.8 Hz, 1H, H2′b), 2.27 (t, *J* = 2.5 Hz, 1H, CH); ^13^C-NMR (75 MHz, CDCl_3_) δ 153.6, 152.6, 148.7, 138.4, 120.0, 87.5, 84.0, 79.7, 71.6, 71.2, 62.4, 40.9, 30.2, 25.6, 25.4, 18.1, 17.7, −5.0, −5.1, −5.7, −5.8; HR-ESI MS (*m*/*z*): [M + H]^+^ calculated for C_25_H_44_N_5_O_3_Si_2_, 518.2977; found 518.2969 [12].

#### 3.1.20. 6-*N*-Propynyl-2′deoxyadenosine (**8**)

To a stirred solution of **28** (0.200 g, 0.386 mmol) in MeOH (15 mL) was added NH_4_F (0.143 g, 3.86 mmol). The reaction mixture was slowly heated to 60–70 °C for 24 h. The solvent was removed in vacuo, and the residue was purified by flash column chromatography on silica gel (MeOH:DCM = 1:9) to give the title compound **8** (88 mg, 75%) as a white solid. ^1^H-NMR (300 MHz, MeOD) δ 8.31 (s, 1H, H8). 8.29 (s, 1H, H2), 6.45 (dd, *J* = 7.9, 6.0 Hz, 1H, H1′), 4.64–4.55 (m, 1H, H3′), 4.42 (s, 2H, NHCH_2_), 4.09 (dd, *J* = 5.6, 2.9 Hz, 1H, H4′), 3.94–3.69 (m, 2H, H5′), 2.83 (ddd, *J* = 13.6, 7.9, 6.0 Hz, 1H, H2′a), 2.62 (t, *J* = 2.5 Hz, 1H, CH), 2.42 (ddd, *J* = 13.4, 6.0, 2.5 Hz, 1H, H2′b); ^13^C-NMR (75 MHz, MeOD) δ 153.9, 151.6, 147.8, 139.7, 119.7, 88.2, 85.4, 79.4, 71.3, 70.5, 61.9, 39.8, 29.2; UV λ_max_ (nm): 265; HR-ESI MS (*m*/*z*): [M + H]^+^ calculated for C_13_H_16_N_5_O_3_, 290.1247; found 290.1252 [12].

#### 3.1.21. *N*^1^-Propynyl-3′,5′-*O*-bis(tert-butyldimethylsilyl)-2′-deoxyinosine (**29**)

To a stirred solution of **26** (0.800 g, 0.67 mmol) in dry THF (30 mL) was added triphenyl phosphine (0.209 g, 0.8 mmol) at rt under N_2_ atm. After 30 min at rt, DEAD (0.300 mL, 1.51 mmol) was added and the mixture was further stirred at rt for 6 h. The crude reaction mixture was concentrated in vacuo, and purified by silica gel column chromatography (EtOAc:hexane = 4:6) to give **29** (0.681 mg, 78%) as a white solid. ^1^H-NMR (300 MHz, CDCl_3_) δ 8.30 (s, 1H, H8), 8.11 (s, 1H, H2), 6.39 (t, *J* = 6.4 Hz, 1H, H1′), 4.89 (t, *J* = 2.8 Hz, 2H, OCH_2_), 4.61 (dt, *J* = 5.7, 3.6 Hz, 1H, H3′), 4.03 (dd, *J* = 6.9, 3.3 Hz, 1H, H4′), 3.81 (qd, *J* = 11.2, 3.6 Hz, 2H, H5′), 2.60–2.50 (m, 2H, H2′b, CH), 2.43 (ddd, *J* = 13.1, 6.1, 3.9 Hz, 1H, H2′a), 0.93 (s, 9H, SiC(CH_3_)_3_), 0.92 (s, 9H, SiC(CH_3_)_3_), 0.12 (s, 6H, SiC(CH_3_)_2_), 0.10 (s, 3H, SiCH_3_), 0.09 (s, 3H, SiCH_3_); ^13^C-NMR (75 MHz, CDCl_3_) δ 155.5, 146.7, 145.5, 138.0, 124.1, 87.7, 83.9, 76.3, 75.2, 71.5, 62.4, 41.3, 34.6, 25.6, 25.4, 18.1, 17.7, −5.0, −5.1, −5.7, −5.8; HR-ESI MS (*m*/*z*): [M + H]^+^ calculated for C_25_H_43_N_4_O_4_Si_2_, 519.2817; found 519.2844.

#### 3.1.22. *N*^1^-Propynyl-2′deoxyinosine (**10**)

To a stirred solution of **29** (0.620 g, 1.19 mmol) in MeOH (30 mL) was added NH_4_F (0.442 g, 11.2 mmol) at rt. The reaction mixture was slowly heated to 60–70 °C for 24 h. The solvent was removed in vacuo, and the residue was purified by flash column chromatography on silica gel (MeOH:DCM = 1:9) to give the title compound **10** (0.279 g, 80%) as a white solid. ^1^H-NMR (300 MHz, MeOD) δ 8.43 (s, 1H, H8), 8.33 (s, 1H, H2), 6.41 (t, *J* = 6.6 Hz, 1H, H1′), 4.91 (d, *J* = 2.4 Hz, 2H, OCH_2_), 4.65–4.50 (m, 1H, H3′), 4.05 (dd, *J* = 6.8, 3.4 Hz, 1H, H4′), 3.79 (qd, *J* = 12.1, 3.8 Hz, 2H, H5′), 2.94 (t, *J* = 2.4 Hz, 1H, CH), 2.74 (dt, *J* = 13.3, 6.6 Hz, 1H, H2′a), 2.49 (ddd, *J* = 13.5, 6.2, 3.5 Hz, 1H, H2′b); ^13^C-NMR (75 MHz, MeOD) δ 155.6, 147.0, 146.8, 139.4, 123.3, 87.9, 84.6, 76.7, 74.1, 70.8, 61.5, 40.0, 34.7; UV λ_max_ (nm): 250; HR-ESI MS (*m*/*z*): [M + H]^+^ calculated for C_13_H_14_N_4_O_4_Na, 313.0907; found 313.0912.

### 3.2. Cell Viability Assay

An amount of 3 × 10^4^ MT4 cells were seeded per well in a 96-well plate. In order to determine the concentration killing 50% of the MT-4 cells (the 50% cytotoxic concentration, CC_50_), the cells were incubated with a dilution series of the nucleoside analogues. In order to determine the concentration achieving 50% protection against HIV (the 50% effective concentration, EC_50_), cells were pre-incubated with a dilution series of the nucleoside analogues for one hour, before they were infected with a fivefold dilution of HIV-1 IIIB virus using an MOI of 0.01, together with the respective analogues. Five days after incubation and infection, the viability was examined with the MTT method. [22] Briefly, 20 µL of a freshly prepared MTT stock solution (7.5 mg/mL in PBS, Sigma-Aldrich, Overijse, Belgium) was added to each well to a final volume of 220 µL. After one hour of incubation at 37 °C, the medium was carefully removed and the purple formazan crystals were solubilized by the addition of 10% triton in acidified isopropanol (0.4% methanesulfonic acid, Sigma Aldrich). The OD was measured at 540 nm with an EnVision 2130 Multilabel Plate Reader (PerkinElmer, Zaventem, Belgium). Data were calculated using the median OD value of three wells. All experiments were performed in triplicate with the table displaying the average for EdU and the lowest CC50 values as found for the propargylated compounds, respectively.

## 4. Conclusions

A series of propargyl-modified purine 2′-deoxynucleosides was successfully synthesized in an effort to develop clickable nucleosides for selective incorporation into and visualization of HIV cDNA. All compounds were devoid of cellular toxicity, and did not inhibit HIV replication, prior requirements for a successful click nucleoside for in vivo visualization techniques. The validity of the idea and the potential for selective staining was briefly shown in this paper. The individual evaluation of the various new clickable analogues as potential selective reporter molecule will be communicated elsewhere. We also believe these clickable nucleosides will find use for various other applications.

## Figures and Tables

**Table 1:** Cell viability and HIV inhibition assay as determined in MT4-cells.

Analogue	CC_50_ (µM)	EC_50_ (µM)
**1** (EdU)	=2.2 ± 1.3	>2.2
**3**	>200	>200
**4**	>600	>600
**5**	>600	>600
**6**	>50	>50
**7**	>1000	>1000
**8**	>1000	>1000
**9**	>600	>600
**10**	>600	>600

## Supplementary Materials

The following are available online, ^1^H and ^13^C-NMR spectra and MS analytical spectra: SI-Spectra Propargyl Project.docx.

## References

[1] Johnson I., Spence M.T.Z. (2010). Molecular Probes Handbook: A Guide to Fluorescent Probes and Labeling Technologies.

[2] Sauer M., Hofkens J., Enderlein J. (2011). Handbook of Fluorescence Spectroscopy and Imaging: From Single Molecules to Ensembles.

[3] Sherer N.M., Lehmann M.J., Jimenez-Soto L.F., Ingmundson A., Horner S.M., Cicchetti G., Allen P.G., Pypaert M., Cunningham J.M., Mothes W. (2003). Visualization of retroviral replication in living cells reveals budding into multivesicular bodies. Traffic.

[4] Close D.M., Xu T.T., Sayler G.S., Ripp S. (2011). In vivo bioluminescent imaging (BLI): Noninvasive visualization and interrogation of biological processes in living animals. Sensors.

[5] Jackson D.A., Pombo A. (1998). Replicon clusters are stable units of chromosome structure: Evidence that nuclear organization contributes to the efficient activation and propagation of s phase in human cells. J. Cell Biol..

[6] Kennedy B.K., Barbie D.A., Classon M., Dyson N., Harlow E. (2000). Nuclear organization of DNA replication in primary mammalian cells. Genes Dev..

[7] Salic A., Mitchison T.J. (2008). A chemical method for fast and sensitive detection of DNA synthesis in vivo. Proc. Natl. Acad. Sci. USA.

[8] Lentz S.I., Edwards J.L., Backus C., McLean L.L., Haines K.M., Feldman E.L. (2010). Mitochondrial DNA (mtDNA) biogenesis: Visualization and duel incorporation of BrdU and EdU into newly synthesized mtDNA in vitro. J. Histochem. Cytochem..

[9] Qu D., Wang G., Wang Z., Zhou L., Chi W., Cong S., Ren X., Liang P., Zhang B. (2011). 5-ethynyl-2′-deoxycytidine as a new agent for DNA labeling: Detection of proliferating cells. Anal. Biochem..

[10] Ligasová A., Liboska R., Friedecký D., Mičová K., Adam T., Oždian T., Rosenberg I., Koberna K. (2016). Dr Jekyll and Mr Hyde: A strange case of 5-ethynyl-2′-deoxyuridine and 5-ethynyl-2′-deoxycytidine. Open Biol..

[11] Borrenberghs D., Dirix L., De Wit F., Rocha S., Blokken J., De Houwer S., Gijsbers R., Christ F., Hofkens J., Hendrix J. (2016). Dynamic oligomerization of integrase orchestrates HIV nuclear entry. Sci. Rep..

[12] Matuszewski M., Kiliszek A., Rypniewski W., Lesnikowski Z.J., Olejniczak A.B. (2015). Nucleoside bearing boron clusters and their phosphoramidites—building blocks for modified oligonucleotide synthesis. New J. Chem..

[13] Jiang H., Congleton J., Liu Q., Merchant P., Malavasi F., Lee H.C., Hao Q., Yen A., Lin H. (2009). Mechanism-based small molecule probes for labeling cd38 on live cells. J. Am. Chem. Soc..

[14] Ghanty U., Fostvedt E., Valenzuela R., Beal P.A., Burrows C.J. (2012). Promiscuous 8-alkoxyadenosines in the guide strand of an SiRNA: Modulation of silencing efficacy and off-pathway protein binding. J. Am. Chem. Soc..

[15] Western E.C., Shaughnessy K.H. (2005). Inhibitory effects of the guanine moiety on Suzuki couplings of unprotected halonucleosides in aqueous media. J. Org. Chem..

[16] Münzel M., Szeibert C., Glas A.F., Globisch D., Carell T. (2011). Discovery and synthesis of new UV-induced intrastrand c(4−8)g and g(8−4)c photolesions. J. Am. Chem. Soc..

[17] Böge N., Gräsl S., Meier C. (2006). Synthesis and properties of oligonucleotides containing c8-deoxyguanosine arylamine adducts of borderline carcinogens. J. Org. Chem..

[18] Torraca K.E., Huang X., Parrish C.A., Buchwald S.L. (2001). An efficient intermolecular palladium-catalyzed synthesis of aryl ethers. J. Am. Chem. Soc..

[19] Dumas A., Luedtke N.W. (2012). Site-specific control of N7–metal coordination in DNA by a fluorescent purine derivative. Chem. A Eur. J..

[20] Taniguchi Y., Fukabori K., Kikukawa Y., Koga Y., Sasaki S. (2014). 2,6-diaminopurine nucleoside derivative of 9-ethyloxy-2-oxo-1,3-diazaphenoxazine (2-amino-adap) for recognition of 8-oxo-dg in DNA. Biorg. Med. Chem..

[21] Kokatla H.P., Lakshman M.K. (2010). One-pot etherification of purine nucleosides and pyrimidines. Org. Lett..

[22] Szucs G., Melnick J.L., Hollinger F.B. (1988). A simple assay based on HIV infection preventing the reclustering of mt-4 cells. Bull. World Health Organ..

[23] Szombati Z., Baerns S., Marx A., Meier C. (2012). Synthesis of c8-arylamine-modified 2′-deoxyadenosine phosphoramidites and their site-specific incorporation into oligonucleotides. ChemBioChem.

[24] Holzberger B., Strohmeier J., Siegmund V., Diederichsen U., Marx A. (2012). Enzymatic synthesis of 8-vinyl- and 8-styryl-2′-deoxyguanosine modified DNA—Novel fluorescent molecular probes. Bioorg. Med. Chem. Lett..

[25] Onizuka K., Taniguchi Y., Sasaki S. (2009). A new odorless procedure for the synthesis of 2′-deoxy-6-thioguanosine and its incorporation into oligodeoxynucleotides. Nucleosides Nucleotides Nucleic Acids.

[26] Buff M.C., Schäfer F., Wulffen B., Müller J., Pötzsch B., Heckel A., Mayer G. (2010). Dependence of aptamer activity on opposed terminal extensions: Improvement of light-regulation efficiency. Nucleic Acids Res..

