# Supplementary material for: Propargylated Purine Deoxynucleosides: New Tools for Fluorescence Imaging Strategies
Source: Molecules. 2019 Jan 28;24(3):468. doi: 10.3390/molecules24030468 (PMC6384747; doi:10.3390/molecules24030468)
Supplement: Supplementary file 1 [file molecules-24-00468-s001.pdf]

Supplementary Information

Propargylated purine deoxynucleosides: new tools for fluorescence imaging strategies

Akkaladevi Venkatesham[1], Sambasiva Rao Pillalimarri[1], Flore De Wit[2], Eveline Lescrinier[1], Zeger Debyser[2], and Arthur Van Aerschot[1,*]

[1] Medicinal Chemistry, Rega Institute for Medical Research, Dept. of Pharmaceutical and Pharmacological Sciences, KU Leuven, Herestraat 49, 3000 Leuven, Belgium; venkat.pallavi2013@gmail.com, sambasivarao.pillalamarri@kuleuven.be, eveline.lescrinier@kuleuven.be, arthur.vanaerschot@kuleuven.be

[2] Laboratory for Molecular Virology and Gene Therapy, Dept. of Pharmaceutical and Pharmacological Sciences, KU Leuven, Kapucijnenvoer 33, 3000 Leuven, Belgium; flore.dewit@kuleuven.be, zeger.debyser@kuleuven.be

* Correspondence: arthur.vanaerschot@kuleuven.be; Tel.: +32-16-372624

Table of contents

1. NMR (1H and 13C) and HRMS (ES+ or ES-) spectra of intermediate and final compounds ……………………………………………………………………………………..2
2. 2D NMR HMBC for compounds 7 and 10 ……………………………………………35
3. Supplementary figure: cell viability dose-response curves ………………………………36

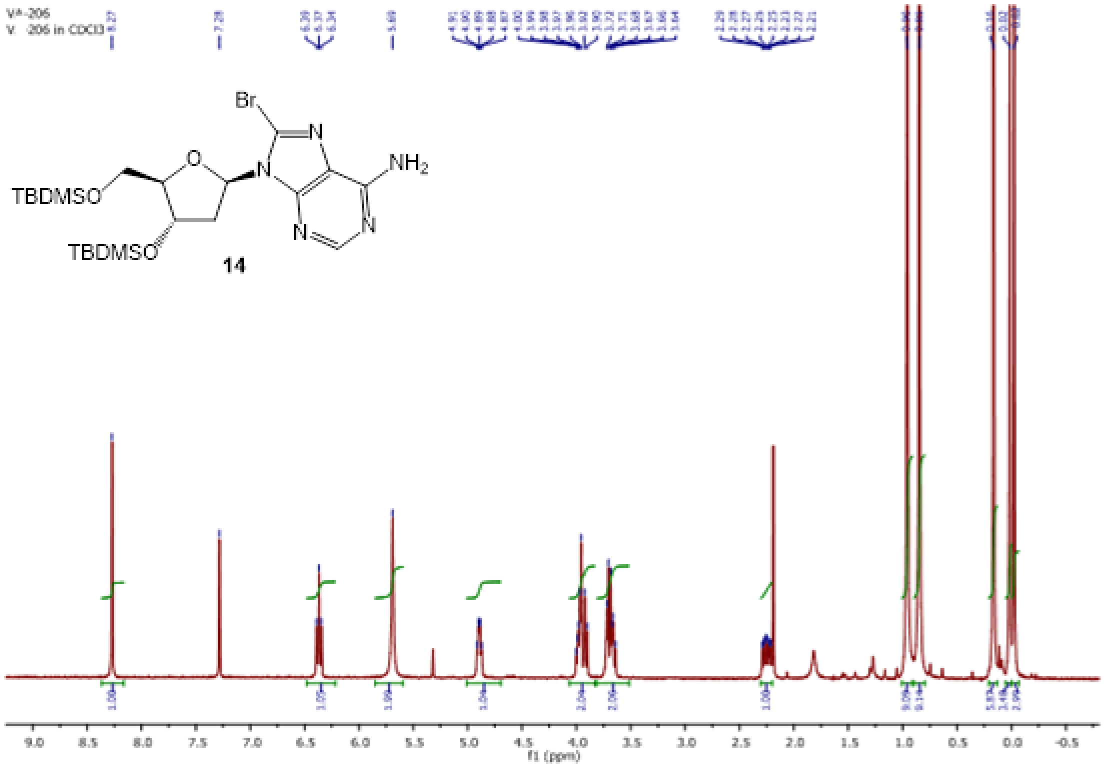
VA-206
V -206 in CDCl3
Br
N
O
NH2
TBDMSO
TBDMSO
14
f1 (ppm)

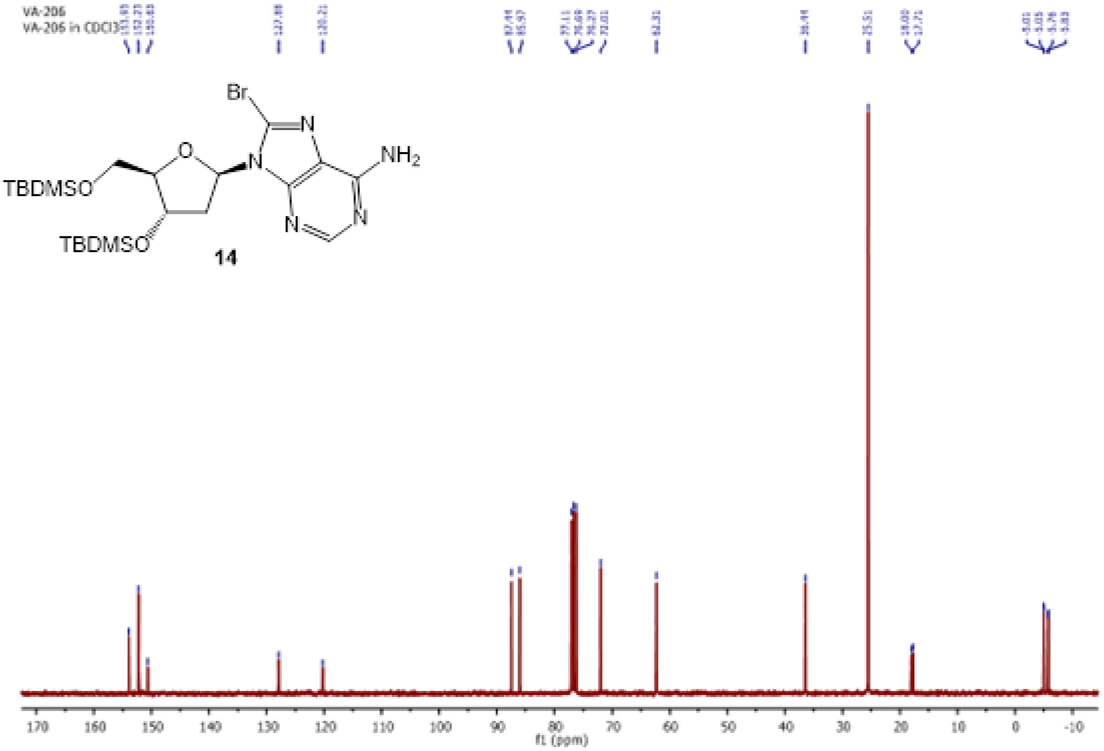
VA-206
VA-206 in CDCl3
Br
N
O
NH2
TBDMSO
TBDMSO
14
f1 (ppm)

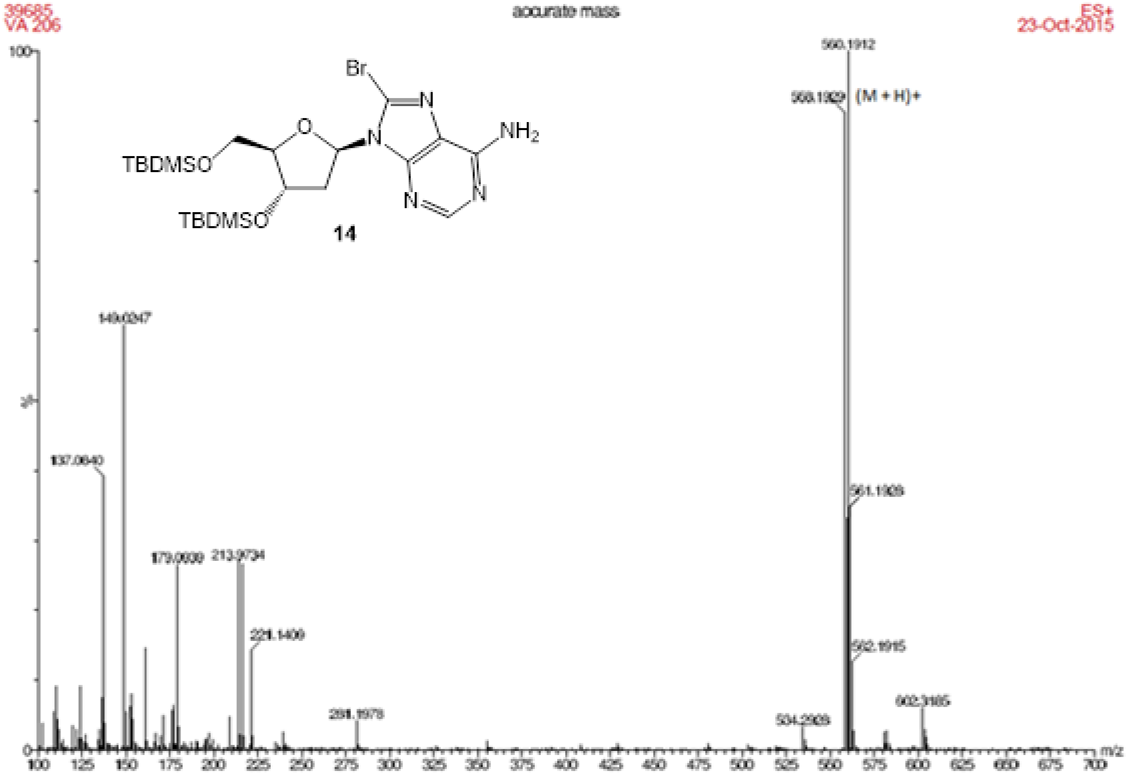

39685
VA 206
accurate mass
ES+
23-Oct-2015
560.1912
558.1929 (M + H)+
149.0247
137.0640
561.1929
179.0939
213.9734
221.1409
562.1915
602.3185
281.1978
534.2928
TBDMSO
TBDMSO
Br
N
NH2
N
N
O
14
m/z

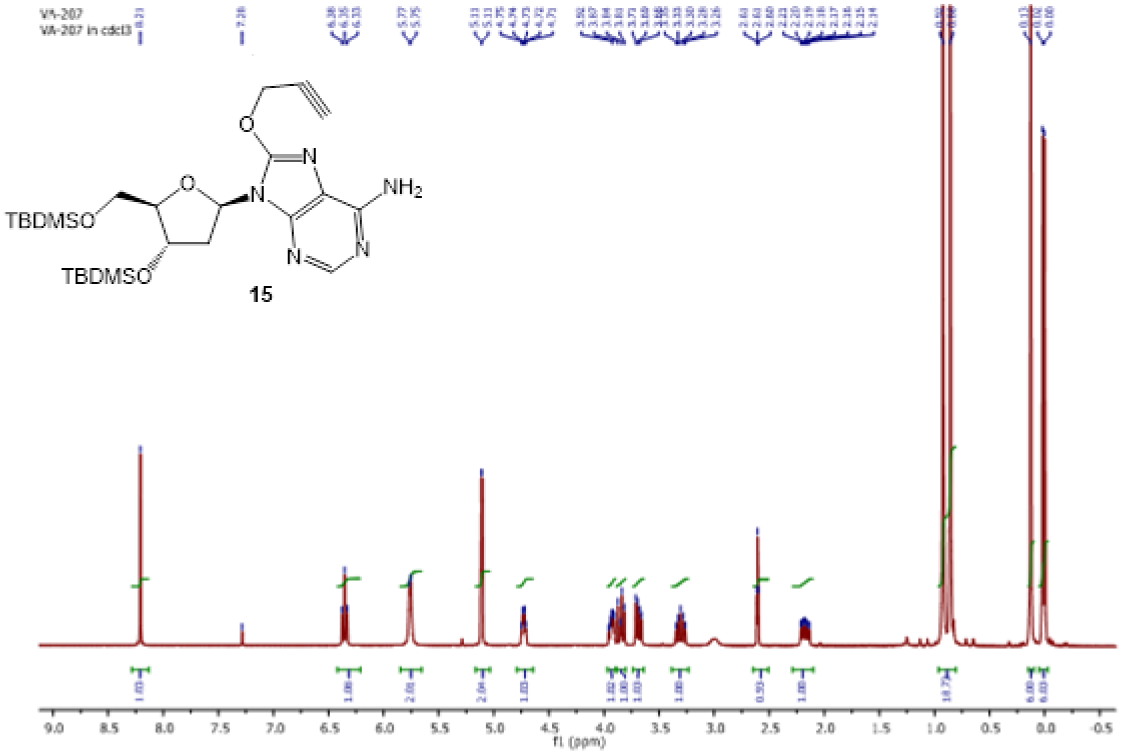

VA-207
VA-207 in cdcl3
TBDMSO
TBDMSO
NH2
15
f1 (ppm)

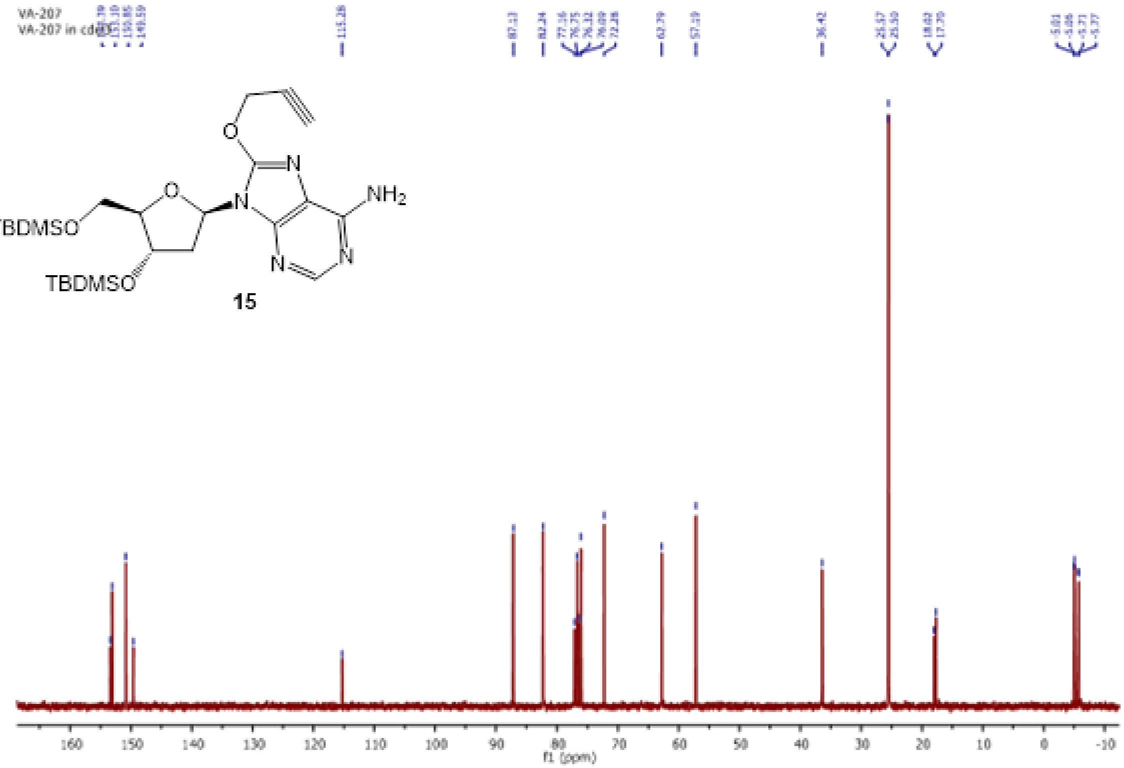

VA-207
VA-207 in cdcl3
TBDMSO
TBDMSO
NH2
15
f1 (ppm)

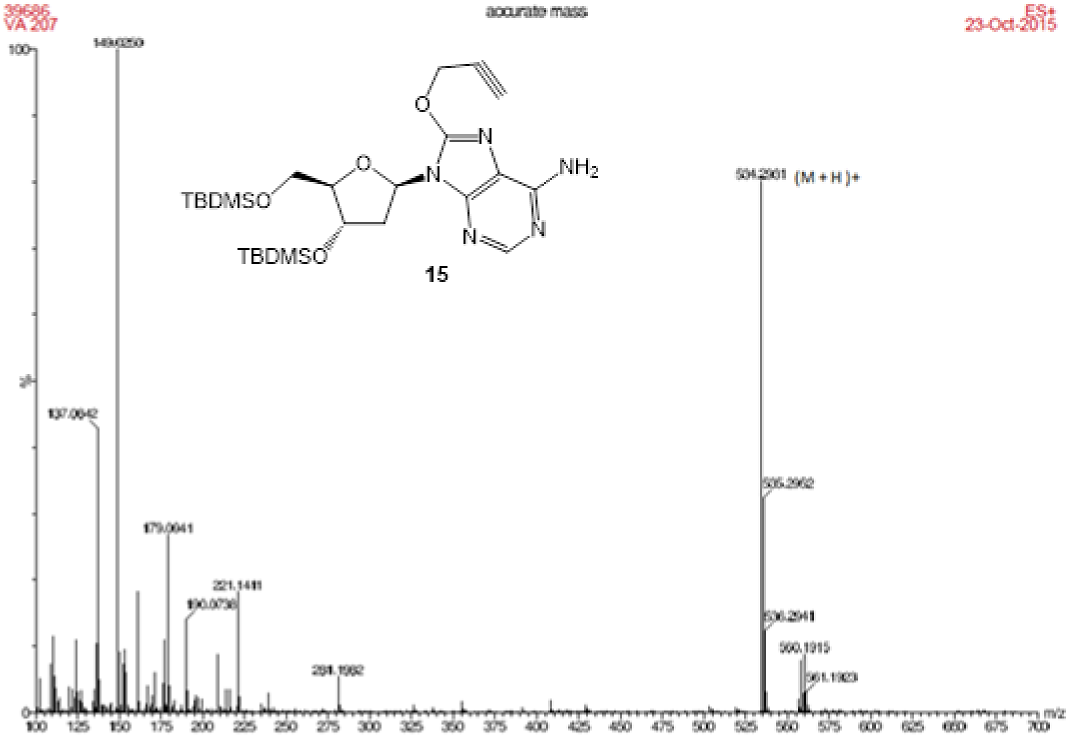

39686
VA 207
accurate mass
ES+
23-Oct-2015
TBDMSO
TBDMSO
NH2
15
149.0259
534.2901 (M + H)+
137.0642
535.2962
179.0941
221.1411
190.0738
536.2941
560.1915
561.1923
281.1582
m/z

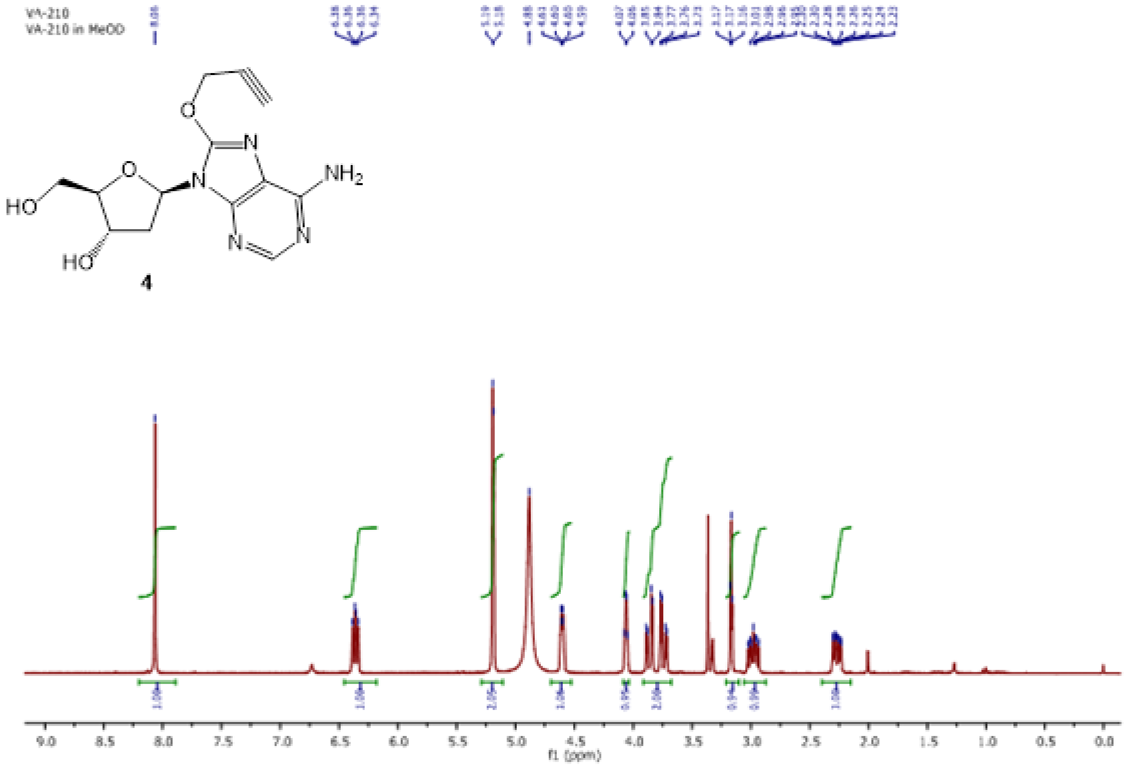
VA-210
VA-210 in MeOD
NH2
HO
HO
4
f1 (ppm)

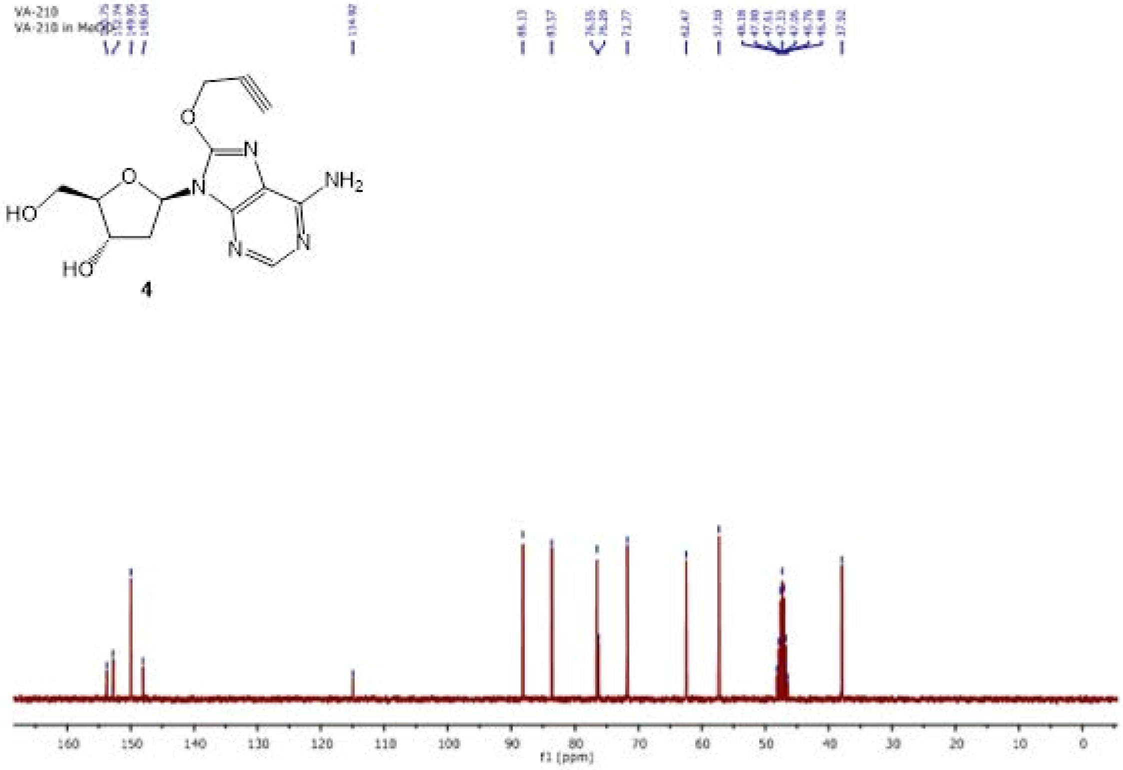
VA-210
VA-210 in MeOD
NH2
HO
HO
4
f1 (ppm)

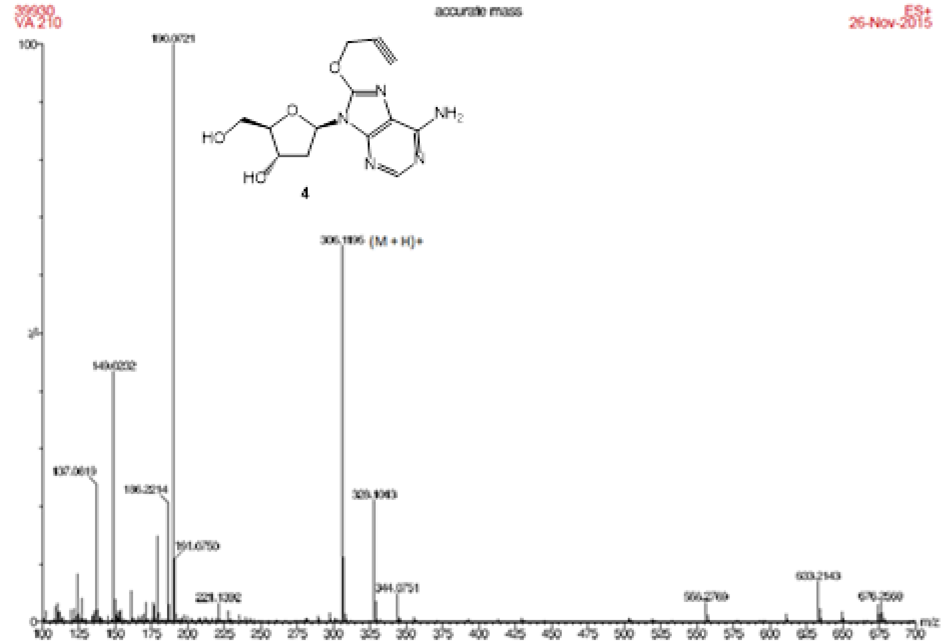
39930
VA 210
accurate mass
ES+
26-Nov-2015
190.0721
306.1196 (M + H)+
149.0232
137.0619
186.2214
329.1063
191.0750
221.1392
344.0731
556.2769
633.2143
676.2560
m/z
4

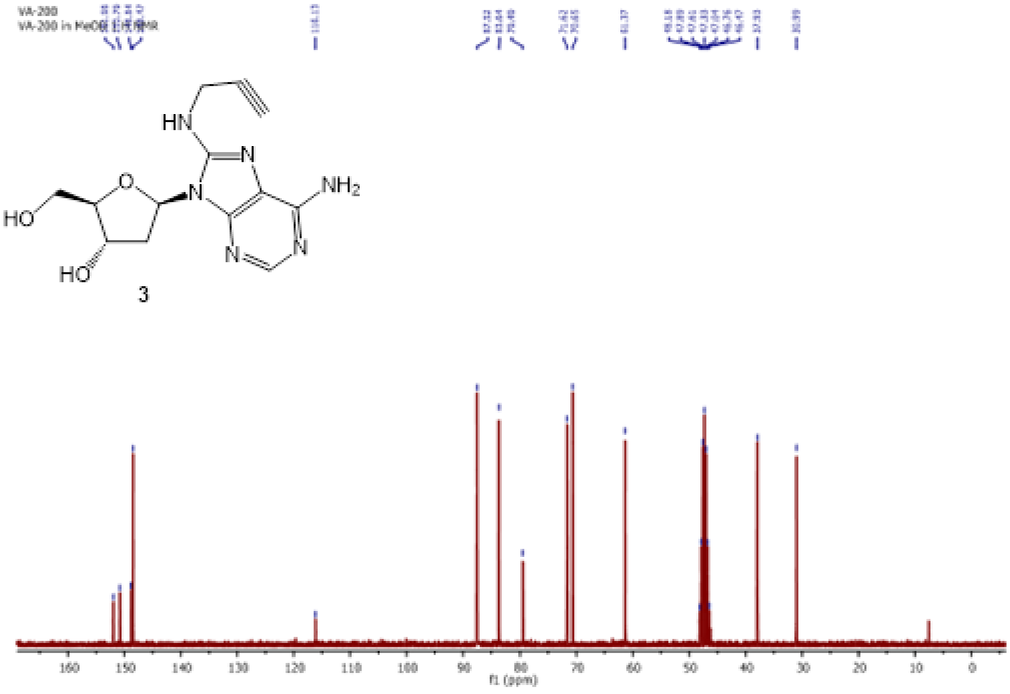
VA-200
HN
NH2
HO
HO
3
f1 (ppm)

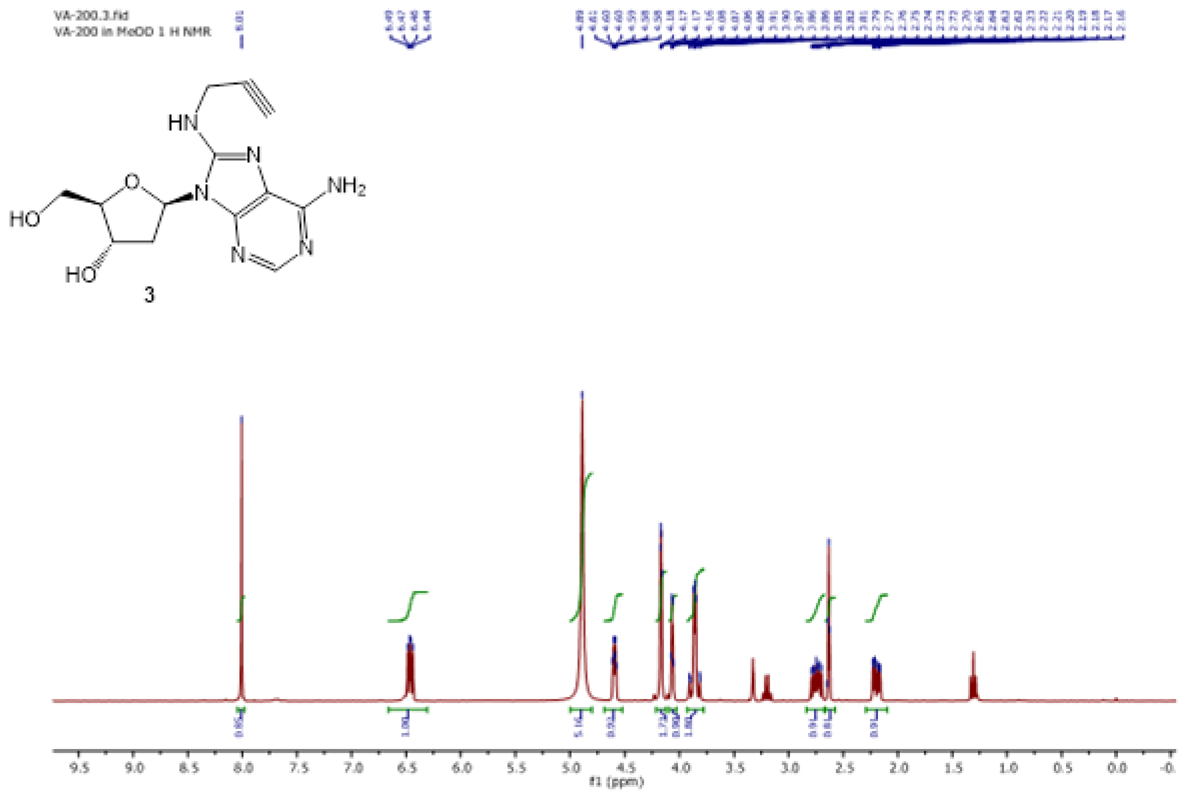
VA-200.3.fid
VA-200 in MeOD 1 H NMR
3
f1 (ppm)

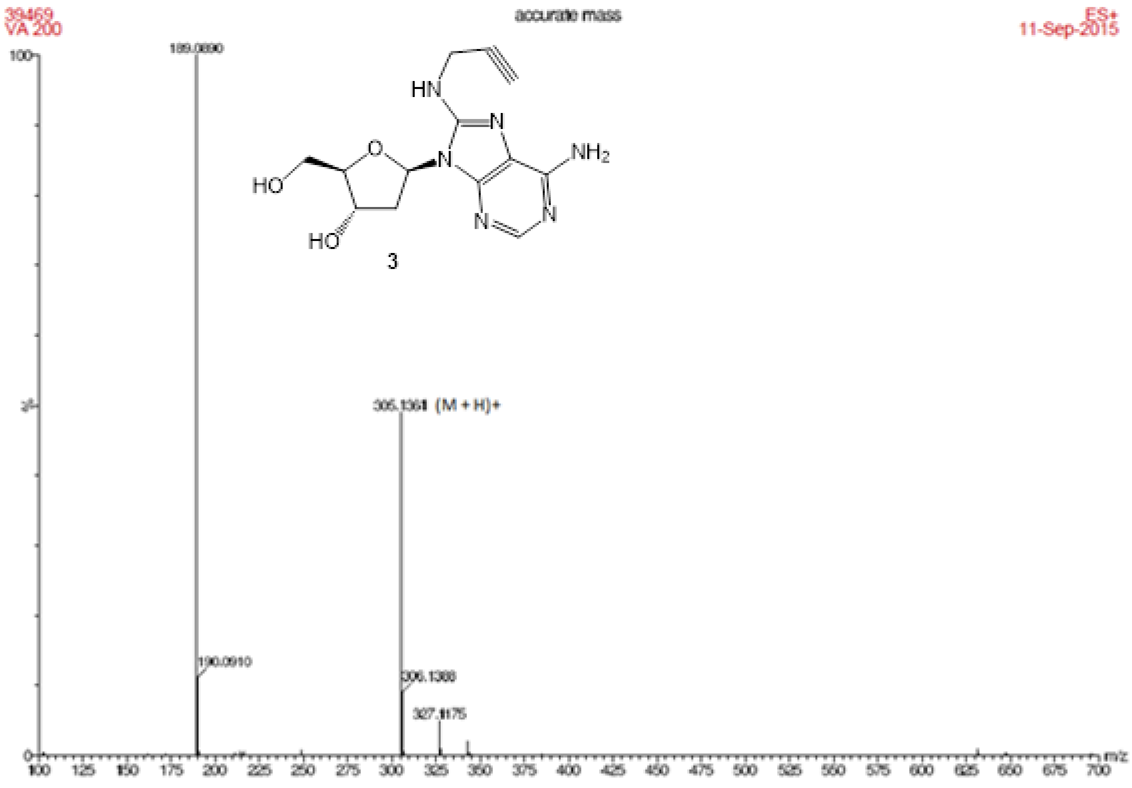
39469
VA 200
accurate mass
ES+
11-Sep-2015
189.0890
(M + H)+
190.0910
306.1388
3

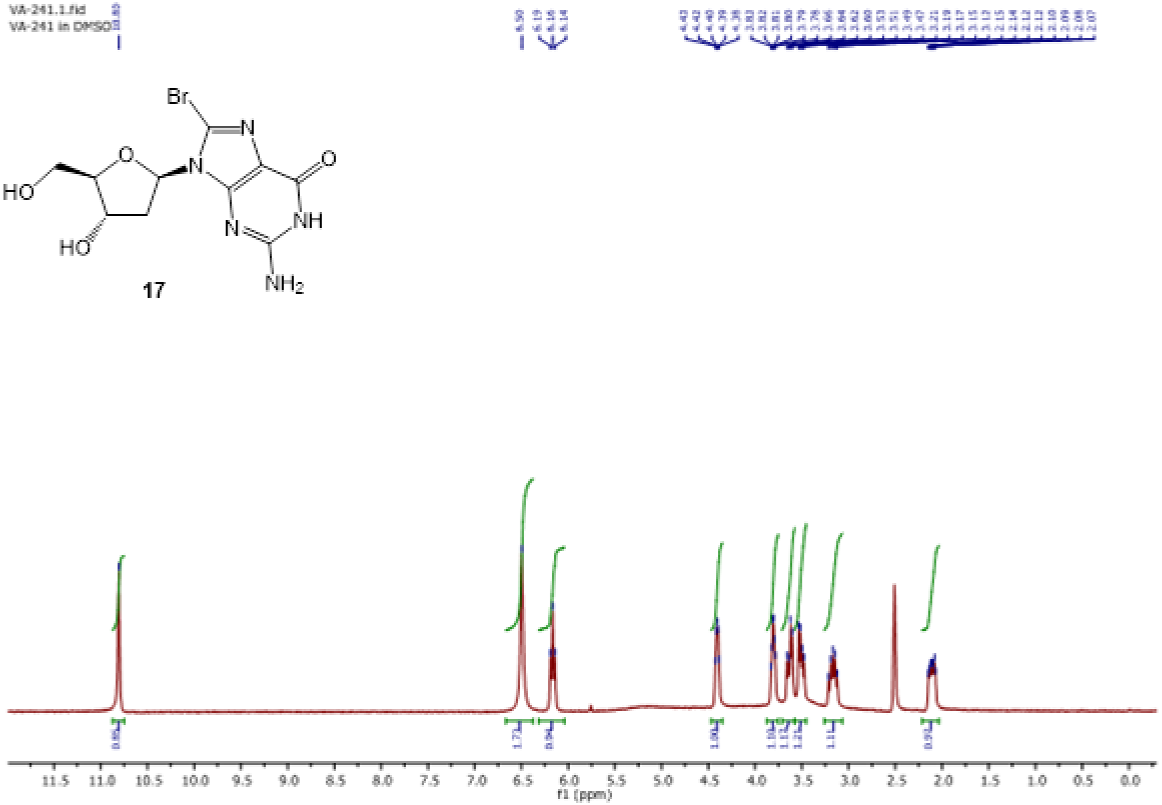

VA-241.1.fid
VA-241 in DMSO
10.83
6.50
6.19
6.16
6.14
Br
N
O
HO
NH
NH2
17
f1 (ppm)

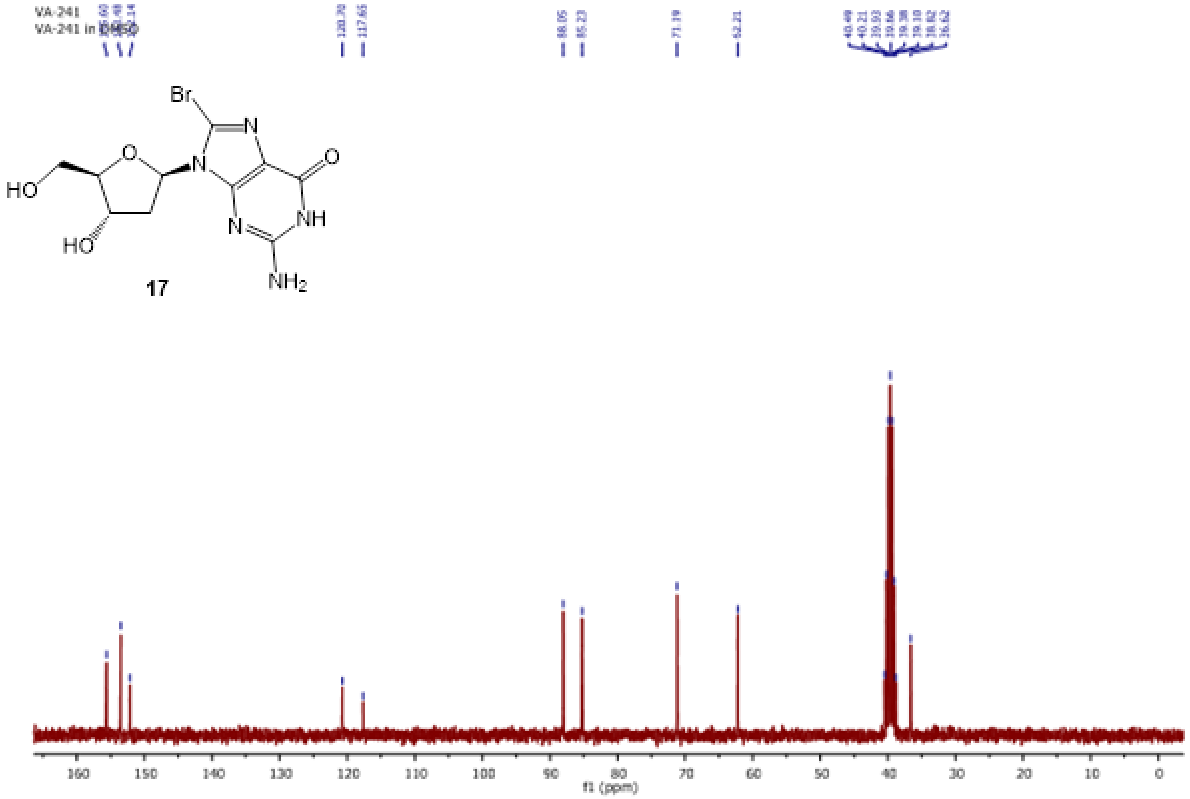

VA-241
VA-241 in DMSO
152.14
120.70
117.65
88.05
85.23
71.19
62.21
40.49
40.21
39.93
39.66
39.38
39.10
38.82
36.62
Br
N
O
HO
NH
NH2
17
f1 (ppm)

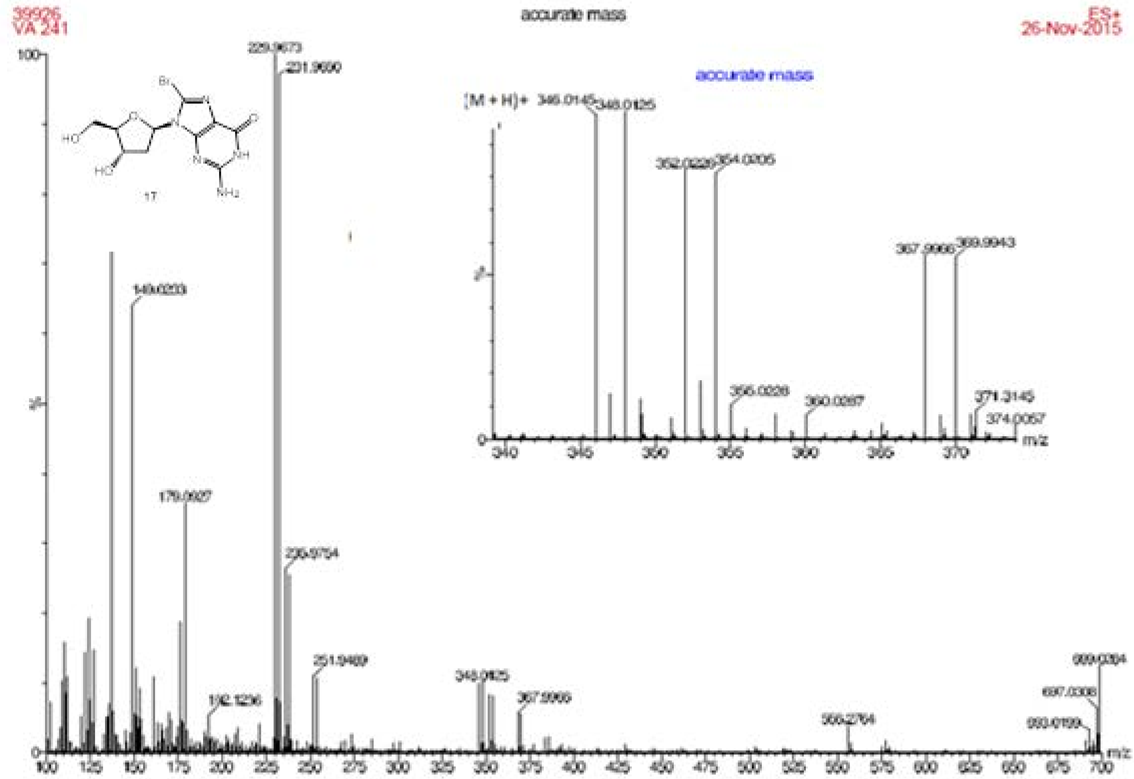
39926
VA 241
accurate mass
ES+
26-Nov-2015
accurate mass
[M + H]+ 346.0145
348.0125
352.0226
354.0205
367.9966
369.9943
229.9673
231.9690
149.0233
179.0927
235.9754
251.0469
17

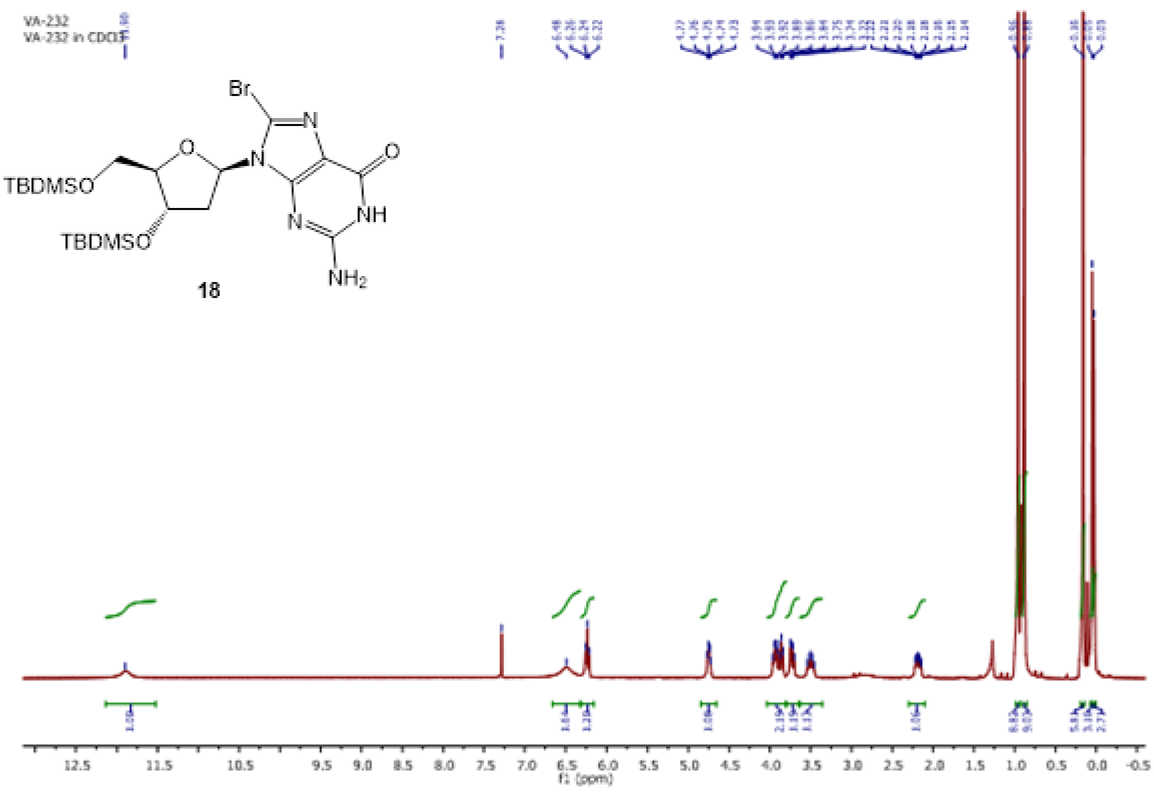
VA-232
VA-232 in CDCl3
TBDMSO
TBDMSO
Br
NH
NH2
18
f1 (ppm)

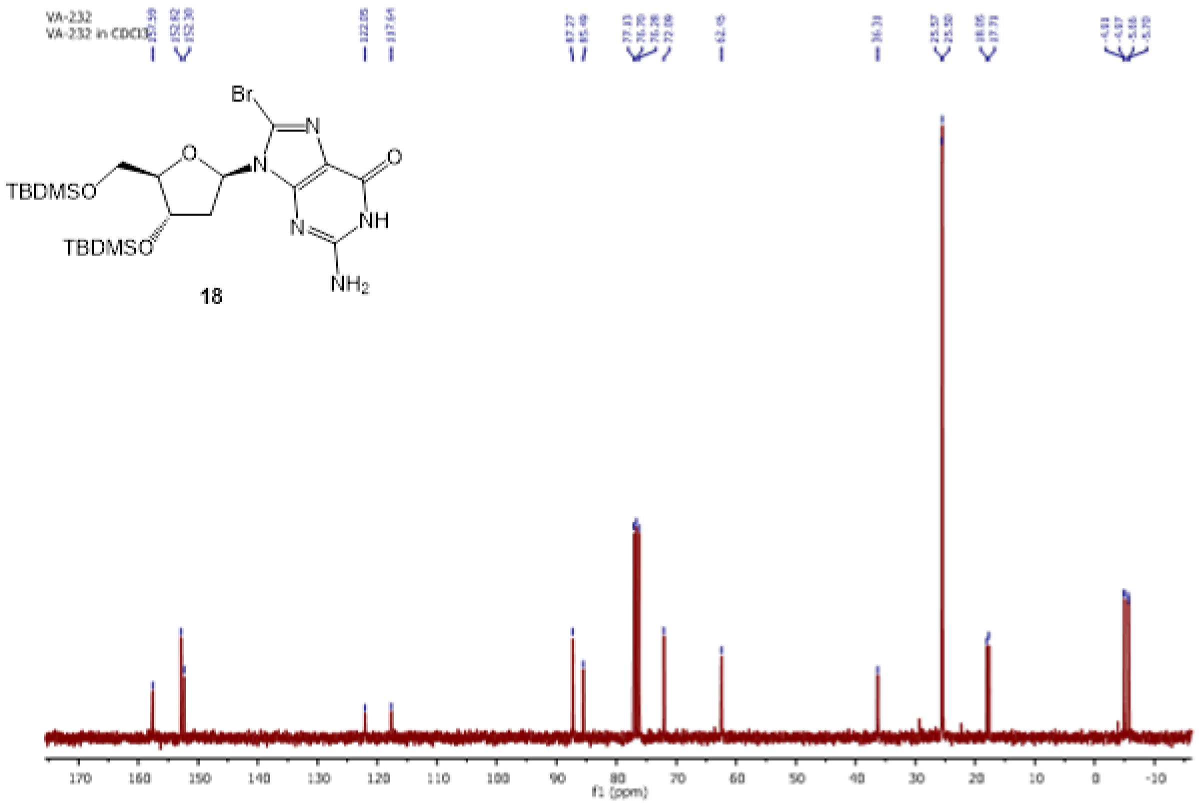
VA-232
VA-232 in CDCl3
Br
N
O
TBDMSO
N
NH
TBDMSO
N
NH2
18
f1 (ppm)

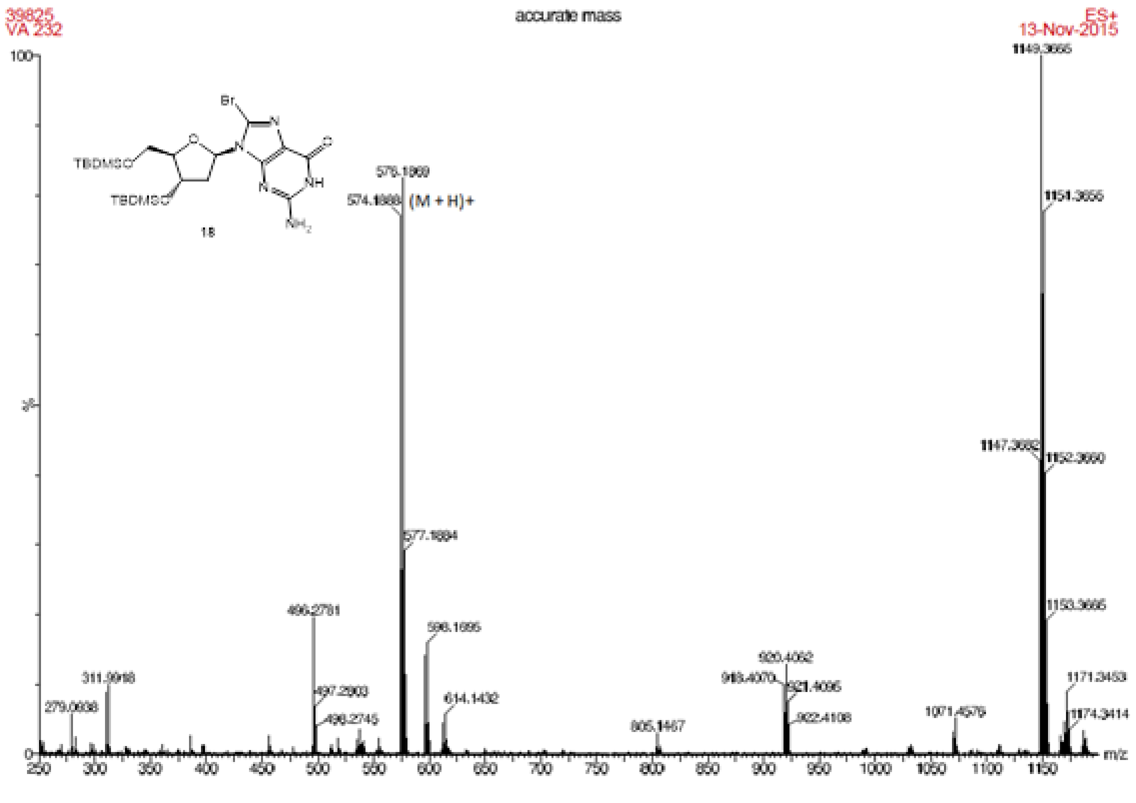
39825
VA 232
accurate mass
ES+
13-Nov-2015
1149.3665
1151.3656
576.1869
574.1888 (M + H)+
1147.3682
1152.3650
577.1884
496.2781
598.1695
1153.3665
311.9918
920.4062
279.0938
497.2903
614.1432
1171.3453
1174.3414
805.1467
922.4108
1071.4579
m/z
18

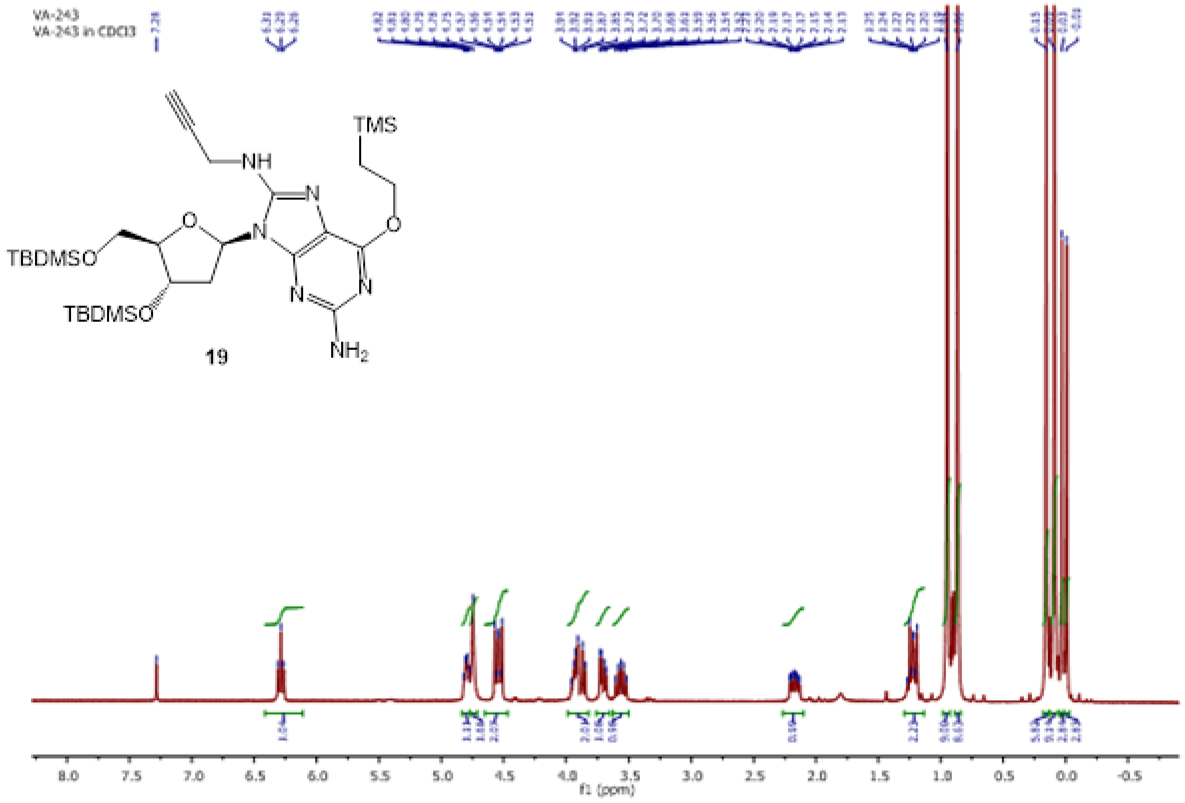
VA-243
VA-243 in CDCl3
TMS
NH
N
O
TBDMSO
TBDMSO
NH_2
19
f1 (ppm)

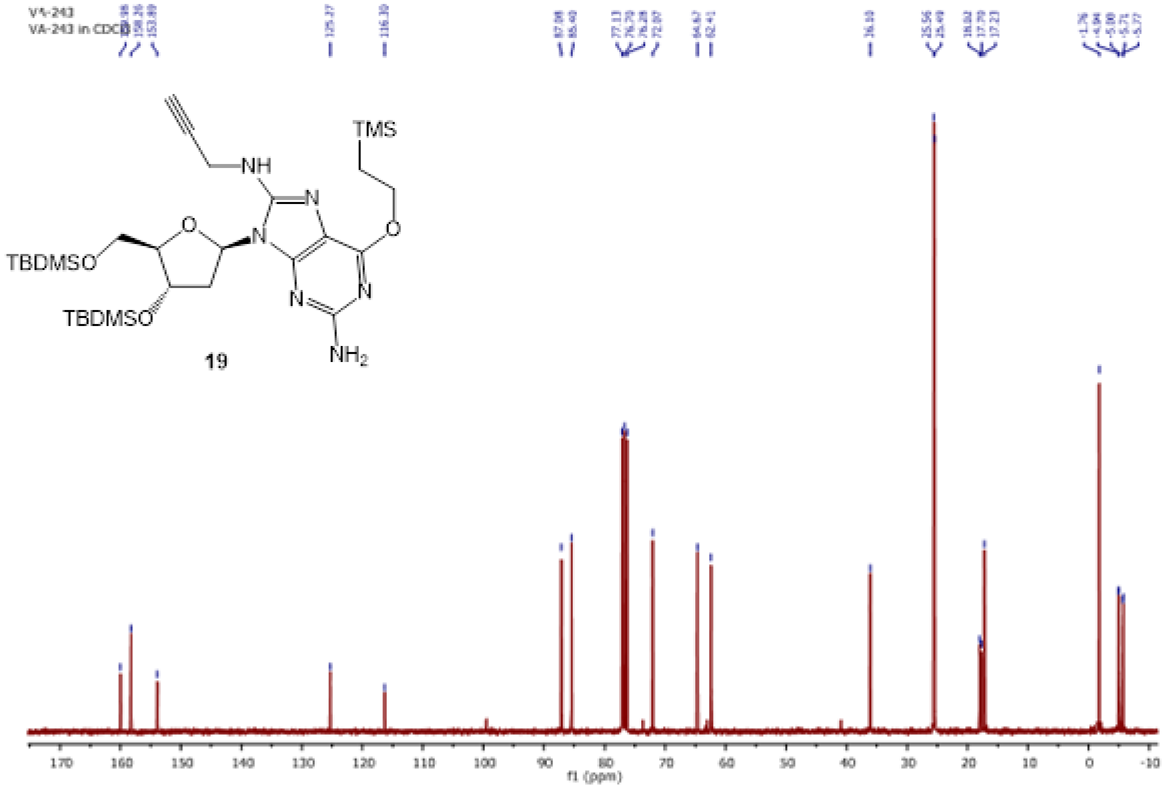
VA-243
VA-243 in CDCl3
TMS
NH
N
O
TBDMSO
TBDMSO
NH_2
19
f1 (ppm)

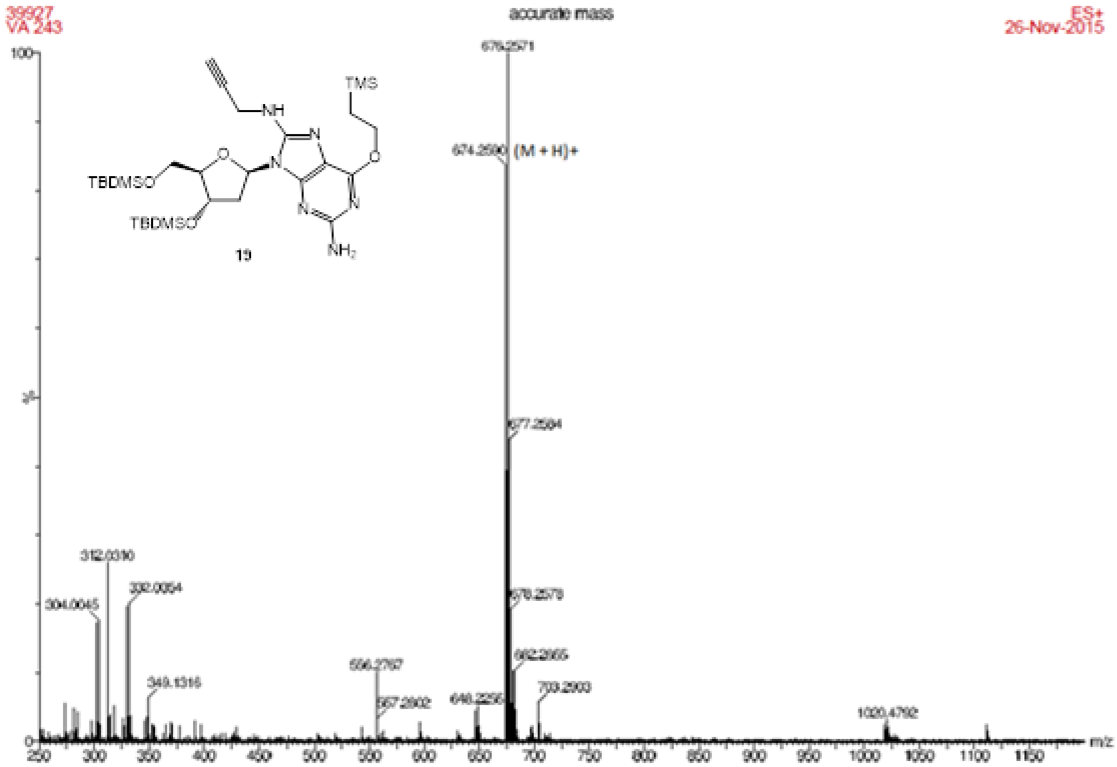
39927
VA 243
accurate mass
ES+
26-Nov-2015
676.2571
674.2590 (M + H)+
677.2584
678.2579
682.2865
703.2903
312.0310
332.0054
304.0045
349.1316
556.2767
648.2256
1020.4792
m/z
TMS
NH
TBDMSO
TBDMSO
NH2
19

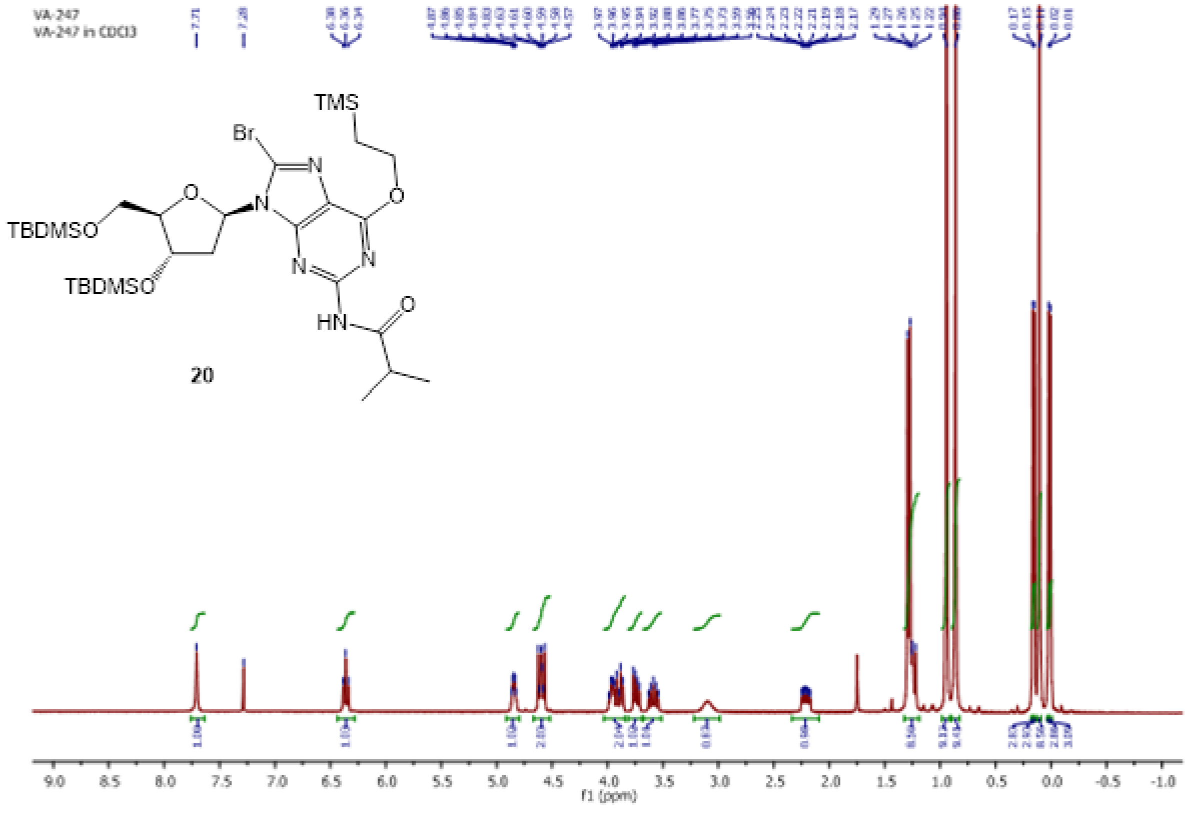
VA-247
VA-247 in CDCl3
TMS
Br
TBDMSO
TBDMSO
HN
20
f1 (ppm)

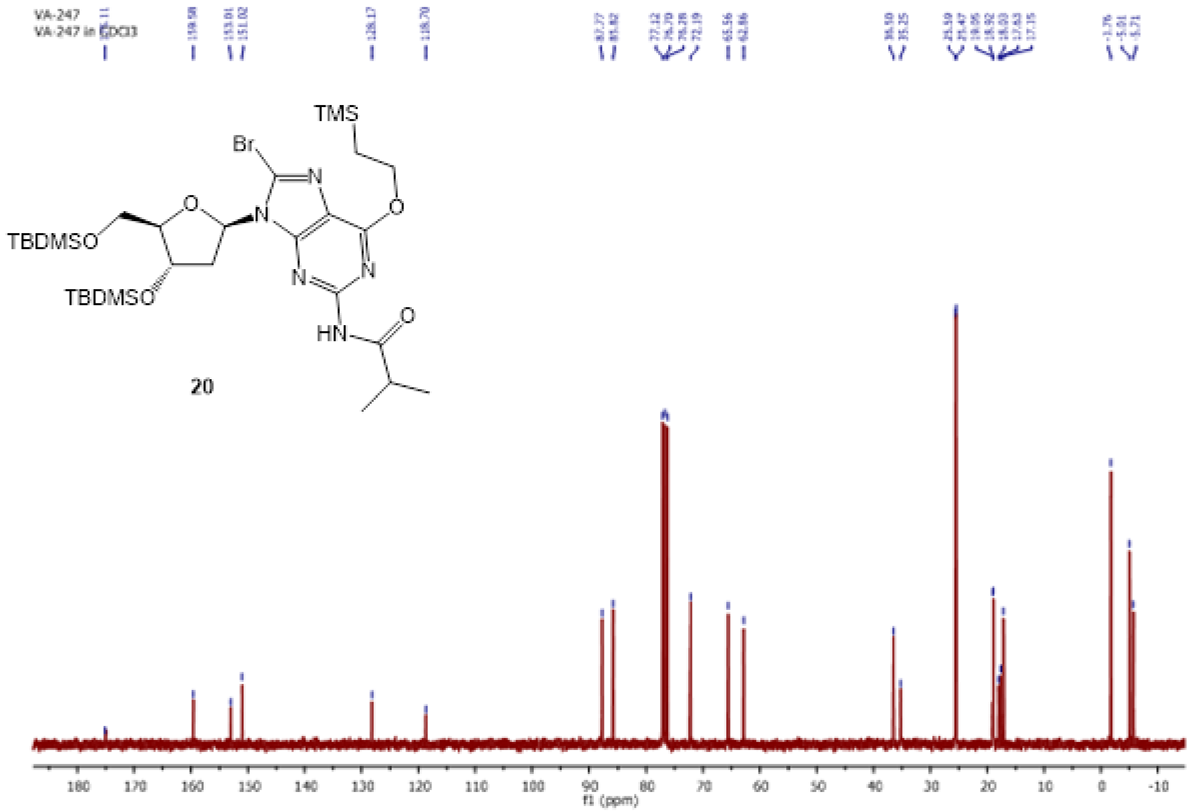

VA-247
VA-247 in CDCl3
TMS
Br
N
O
TBDMSO
TBDMSO
N
N
HN
O
20
180 170 160 150 140 130 120 110 100 90 80 70 60 50 40 30 20 10 0 -10
f1 (ppm)

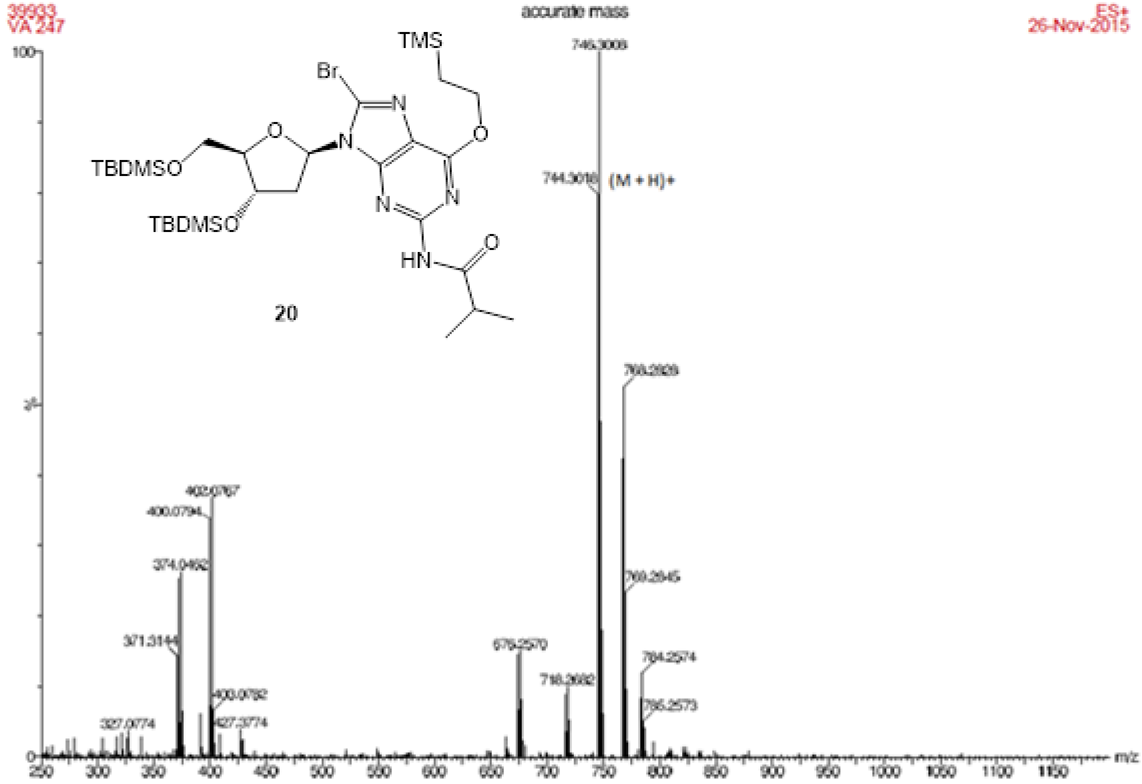

39933
VA 247
accurate mass
ES+
26-Nov-2015
TMS
Br
N
O
TBDMSO
TBDMSO
N
N
HN
O
20
745.3008
744.3018 (M + H)+
768.2929
769.2945
402.0767
400.0794
374.0492
371.3144
676.2570
718.2692
784.2574
785.2573
400.0792
427.3774
327.0774
100
%
0
250 300 350 400 450 500 550 600 650 700 750 800 850 900 950 1000 1050 1100 1150
m/z

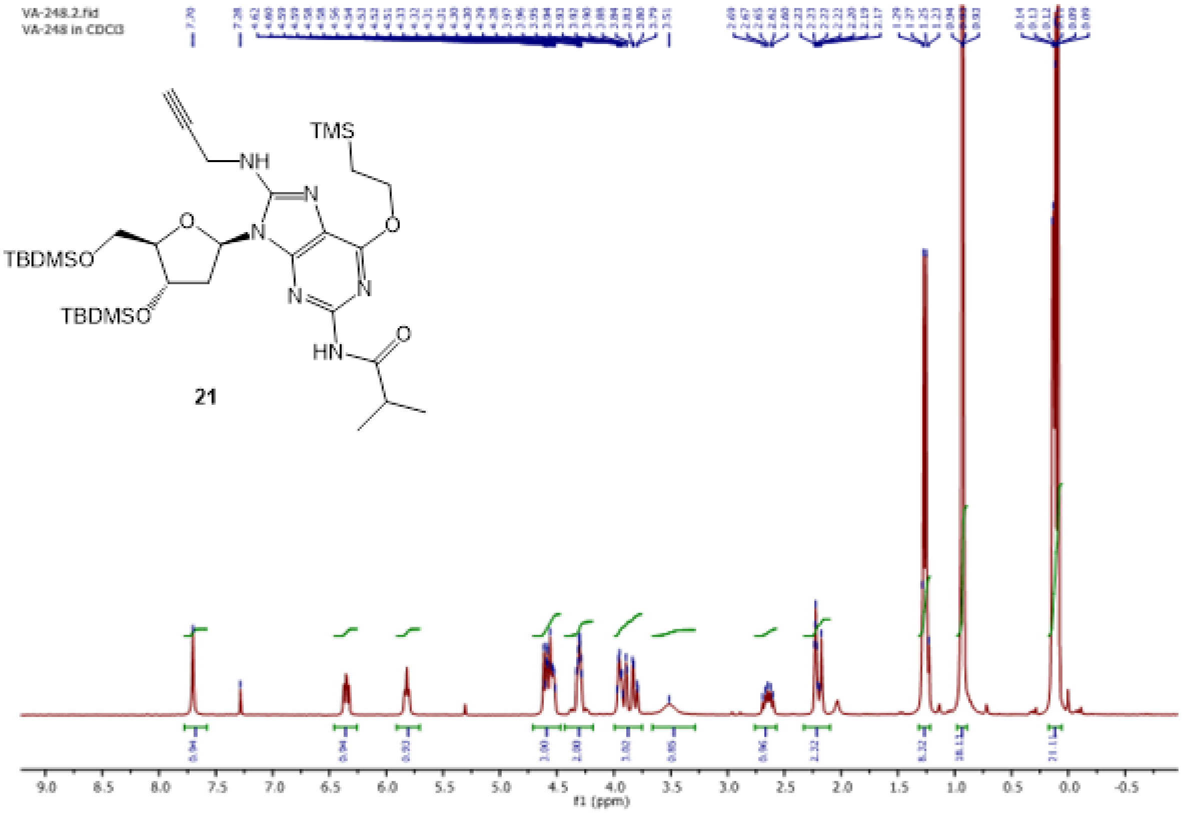
VA-248.2.fid
VA-248 in CDCl3
TMS
NH
TBDMSO
TBDMSO
HN
21
f1 (ppm)

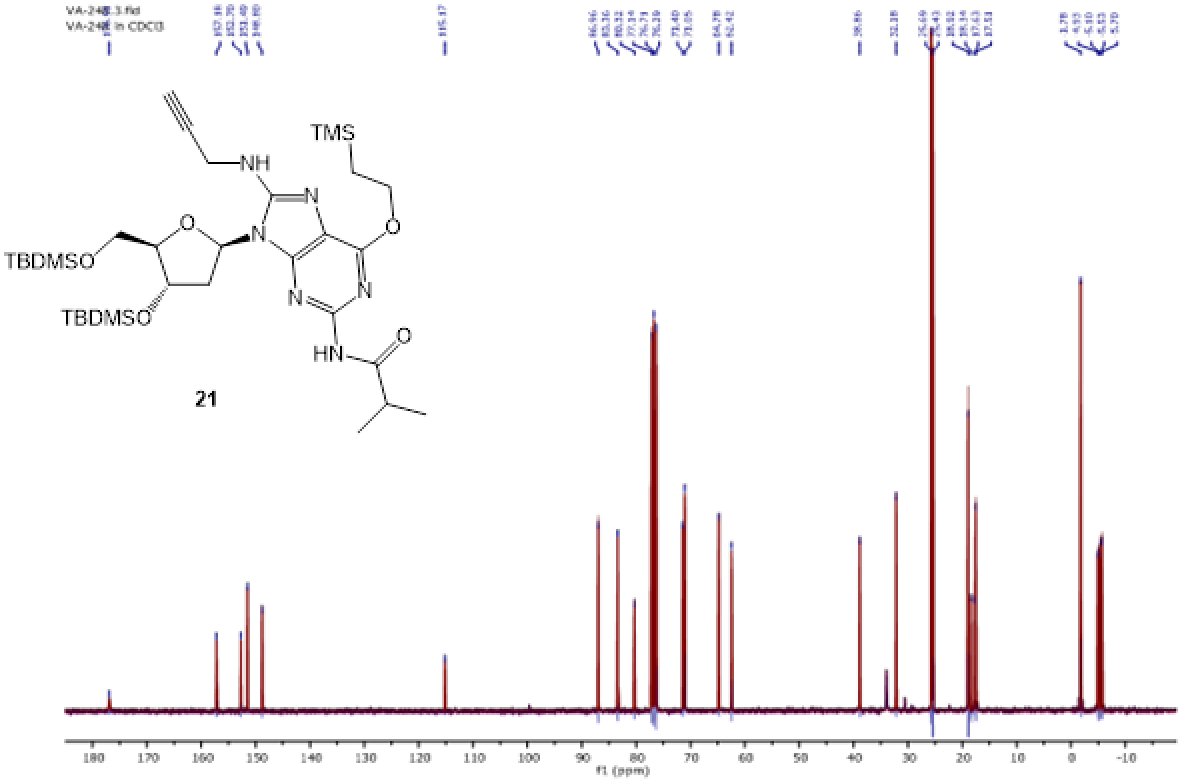
TMS
NH
TBDMSO
TBDMSO
HN
21
f1 (ppm)

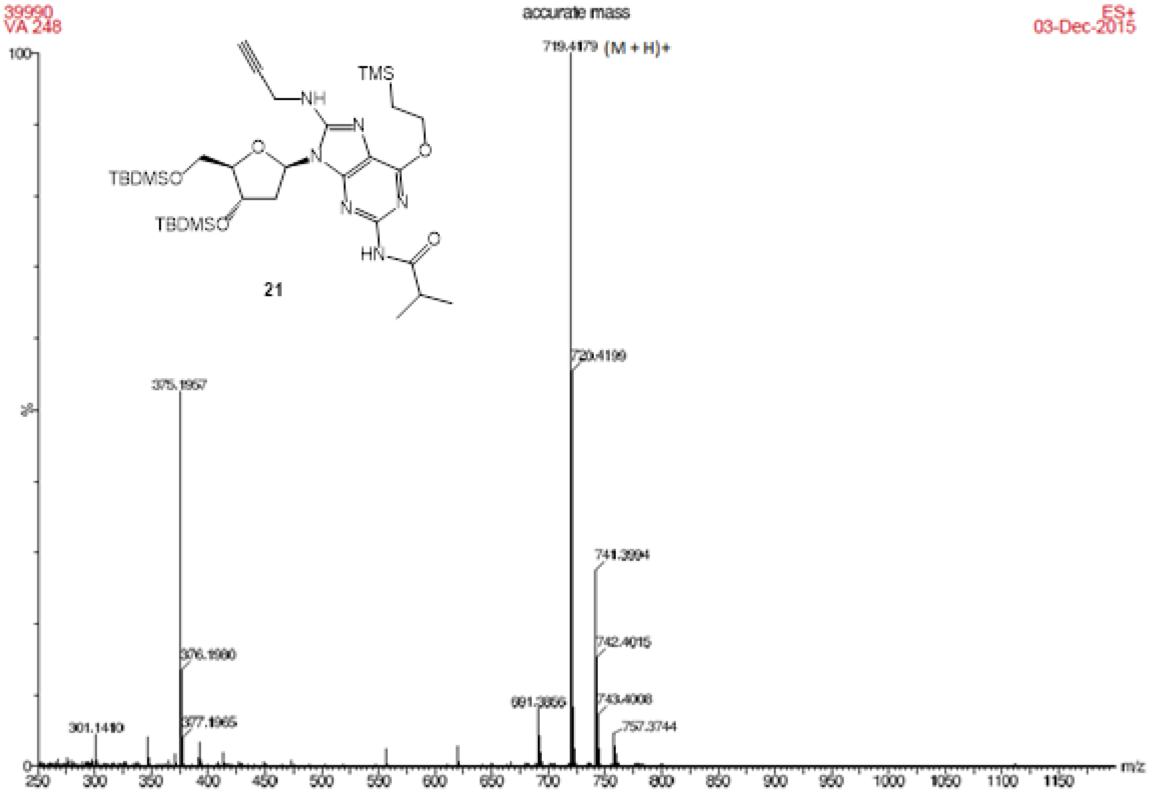
39990
VA 248
accurate mass
ES+
03-Dec-2015
719.4179 (M + H)+
720.4199
375.1957
741.3994
742.4015
376.1980
691.3856
743.4008
301.1410
377.1965
757.3744
TMS
NH
TBDMSO
TBDMSO
HN
21
m/z

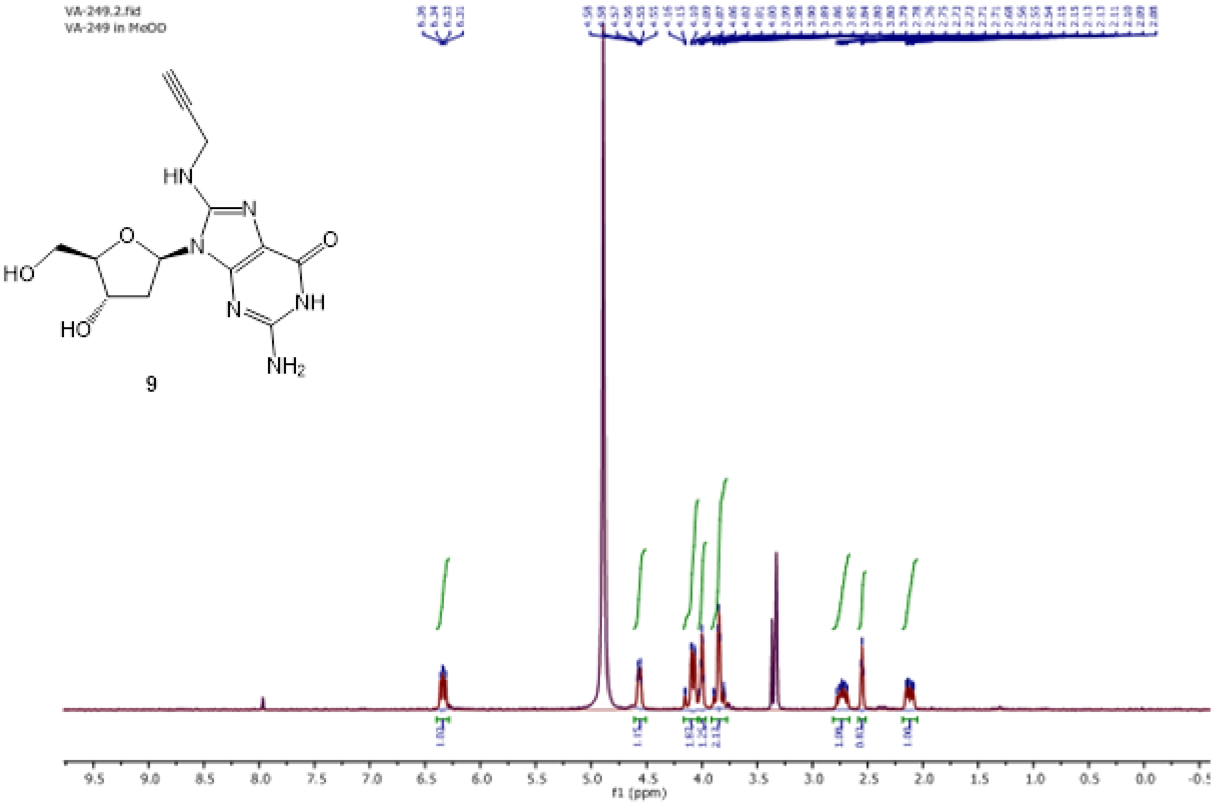
VA-249.2.fid
VA-249 in MeOD
HN
HO
HO
NH
NH2
9
f1 (ppm)

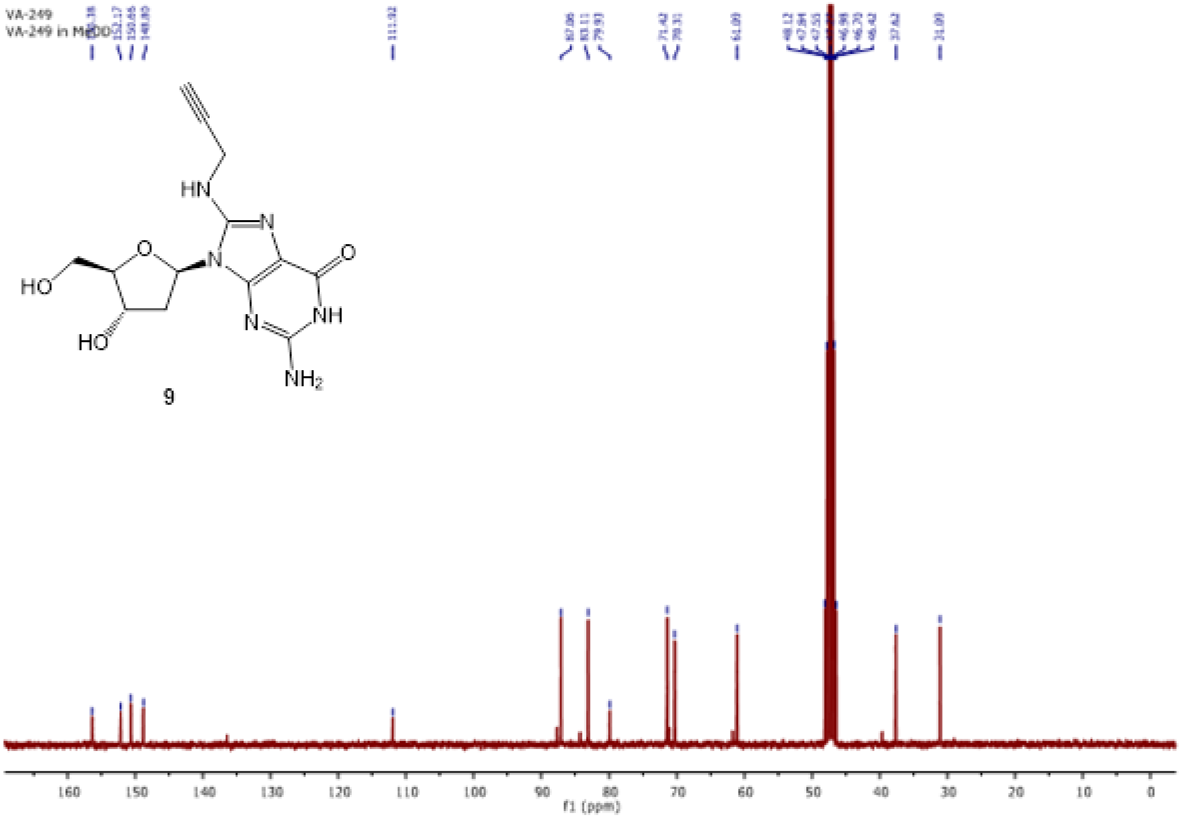
VA-249
VA-249 in MeOD
152.17
150.66
148.80
111.92
87.06
83.11
79.93
71.42
70.31
61.09
48.12
47.84
47.55
46.98
46.70
46.42
37.62
31.09
HN
N
O
N
HO
HO
NH
N
NH2
9
f1 (ppm)

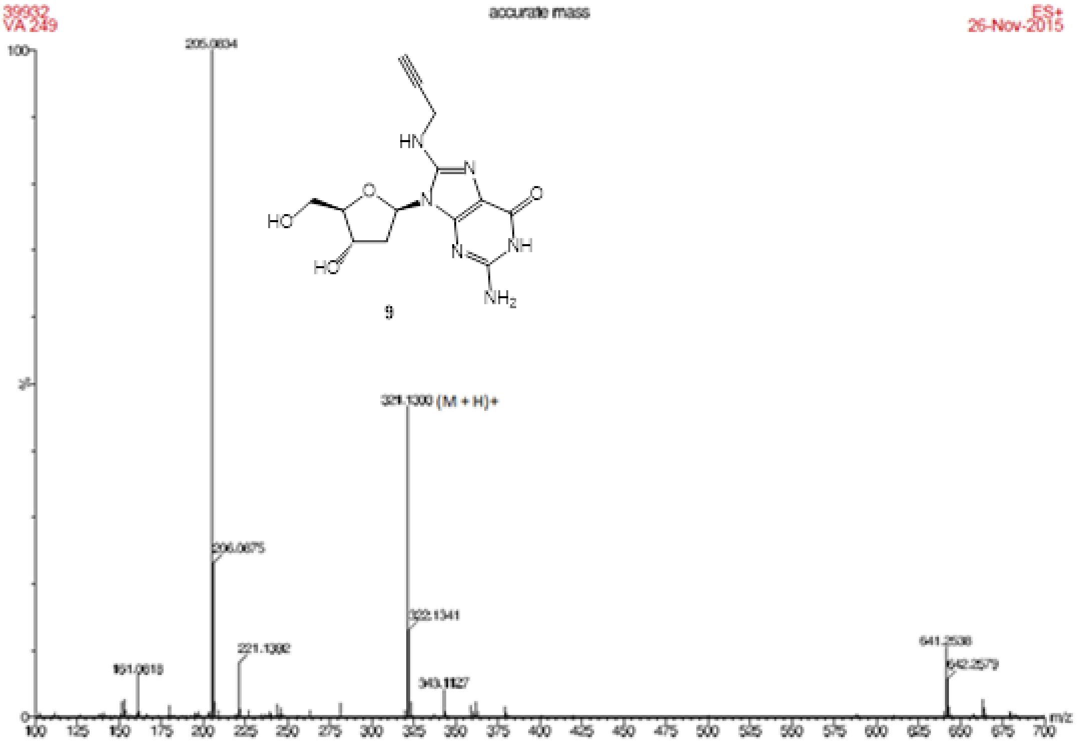
39932
VA 249
accurate mass
ES+
26-Nov-2015
205.0834
321.1300 (M + H)+
206.0875
322.1341
221.1392
161.0610
343.1127
641.2538
642.2579
HN
N
O
N
HO
HO
NH
N
NH2
9
m/z

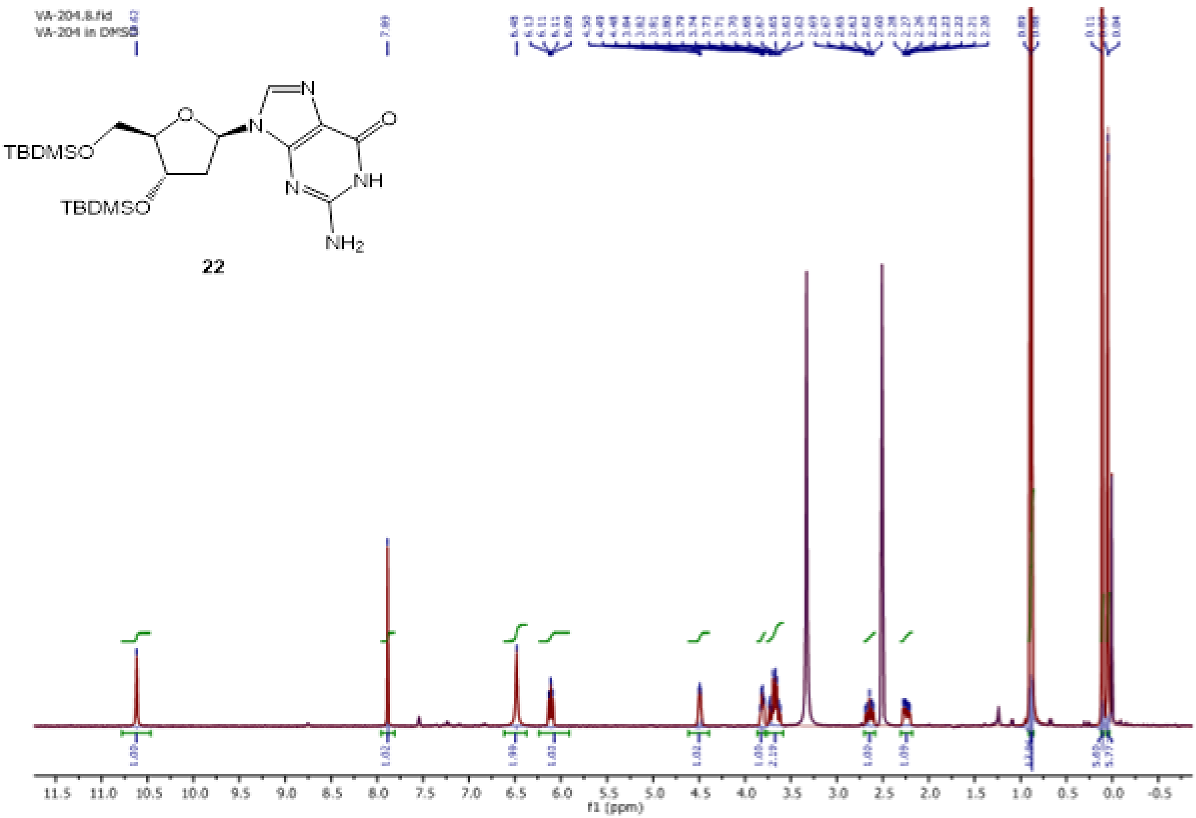
VA-204.8.fid
VA-204 in DMSO
TBDMSO
TBDMSO
NH
NH2
22
f1 (ppm)

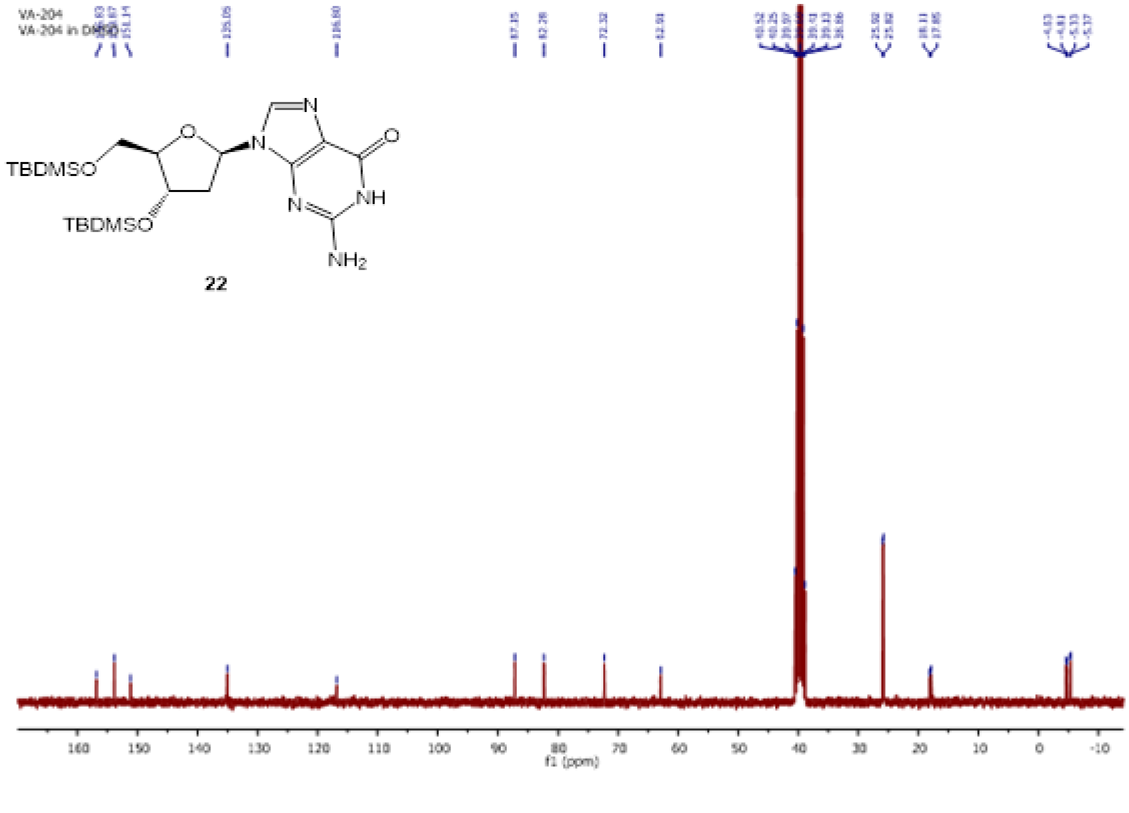
VA-204
VA-204 in DMSO
TBDMSO
TBDMSO
NH
NH2
22
f1 (ppm)

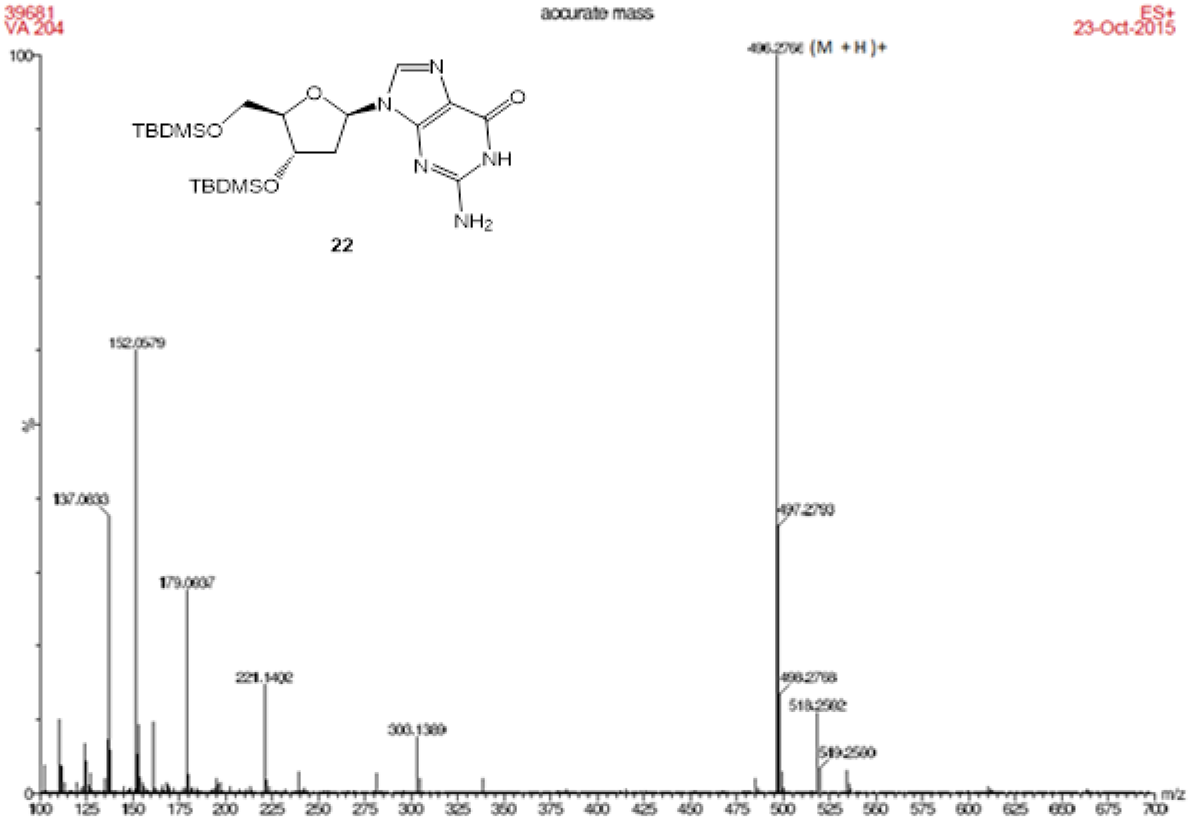

39681
VA 204
accurate mass
ES+
23-Oct-2015
496.2766 (M + H)+
TBDMSO
TBDMSO
22
152.0579
137.0833
497.2793
179.0837
221.1402
498.2768
518.2582
303.1389
519.2560
m/z

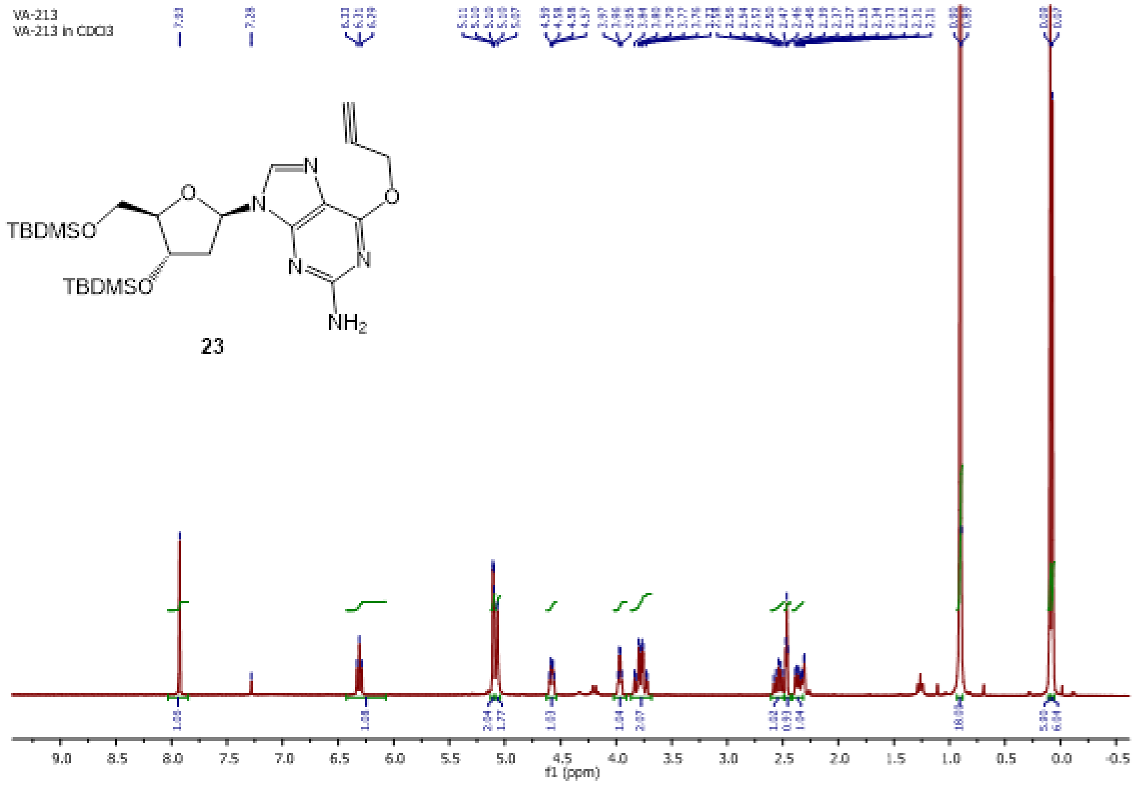

VA-213
VA-213 in CDCl3
TBDMSO
TBDMSO
23
f1 (ppm)

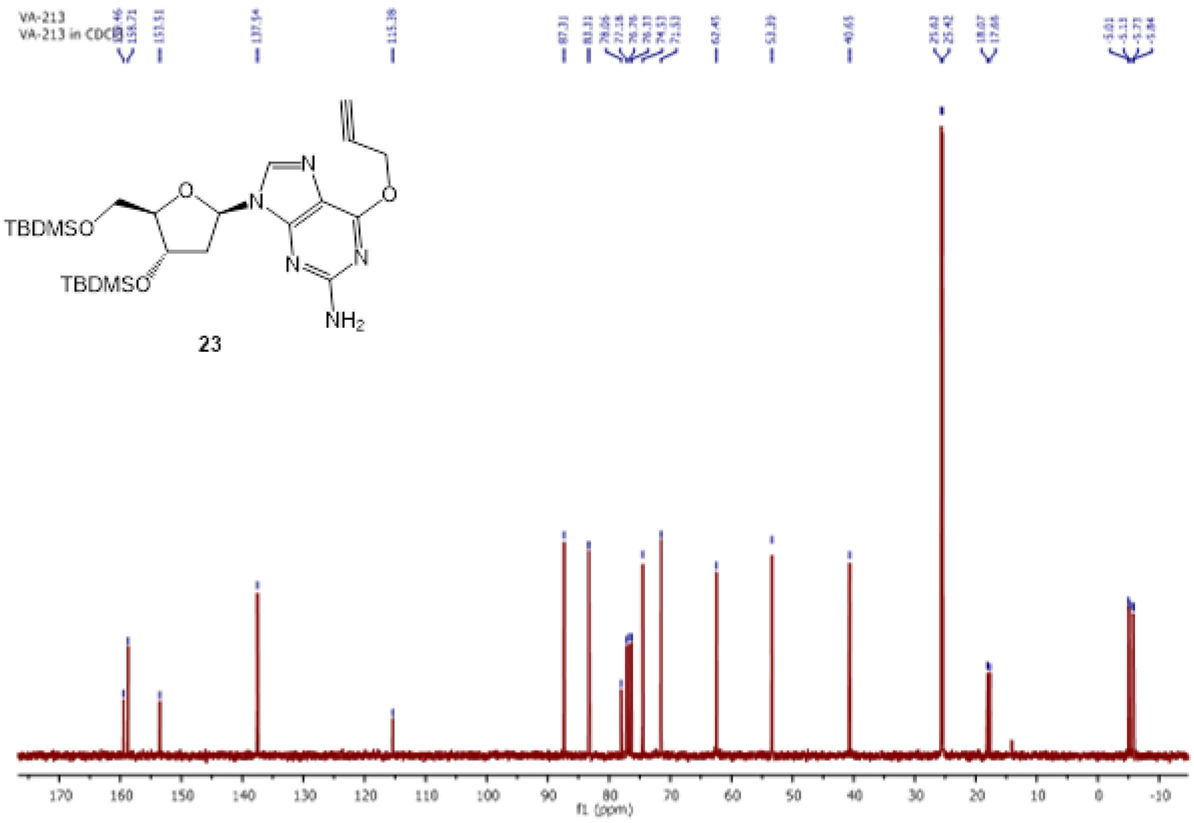
VA-213
VA-213 in CDCl3
159.46
158.71
153.51
137.54
115.38
87.31
83.31
78.06
77.18
76.76
76.33
74.53
71.53
62.45
53.39
40.65
25.62
25.42
18.07
17.66
-5.01
-5.13
-5.73
-5.84
TBDMSO
TBDMSO
N
N
N
N
O
O
NH2
23
170 160 150 140 130 120 110 100 90 80 70 60 50 40 30 20 10 0 -10
f1 (ppm)

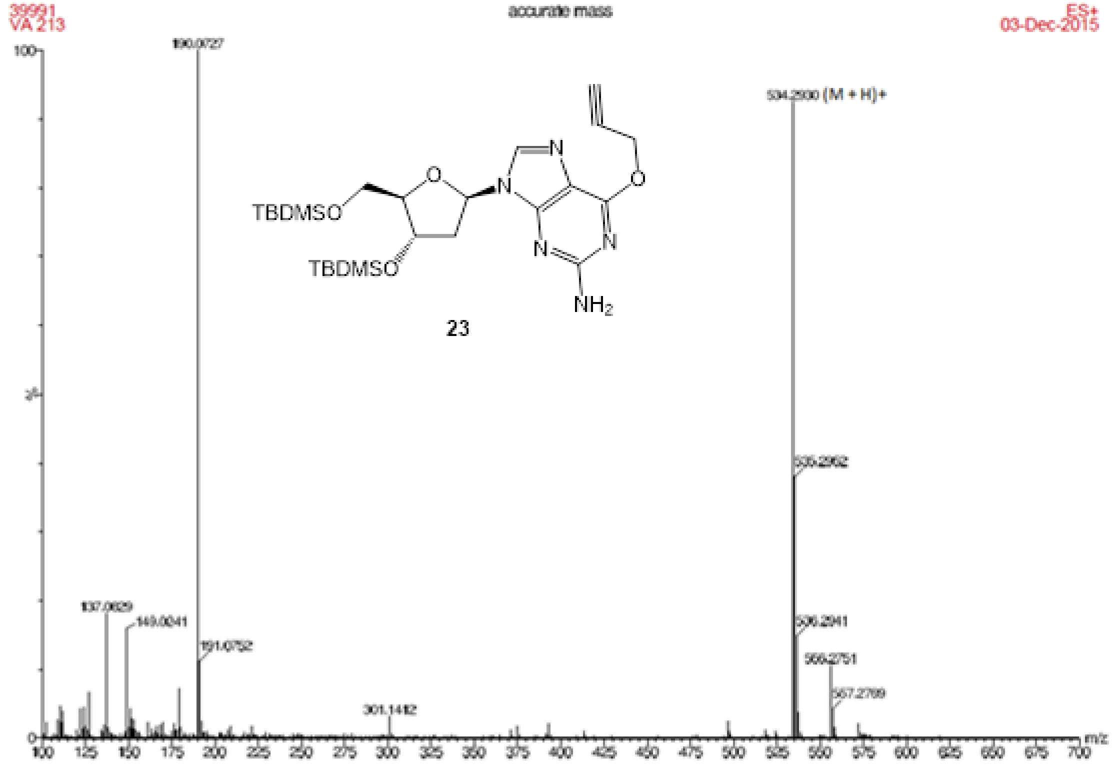
39991
VA 213
accurate mass
ES+
03-Dec-2015
100
%
0
190.0727
534.2930 (M + H)+
535.2962
536.2941
556.2751
557.2769
137.0829
149.0241
191.0752
301.1412
TBDMSO
TBDMSO
N
N
N
N
O
O
NH2
23
100 125 150 175 200 225 250 275 300 325 350 375 400 425 450 475 500 525 550 575 600 625 650 675 700
m/z

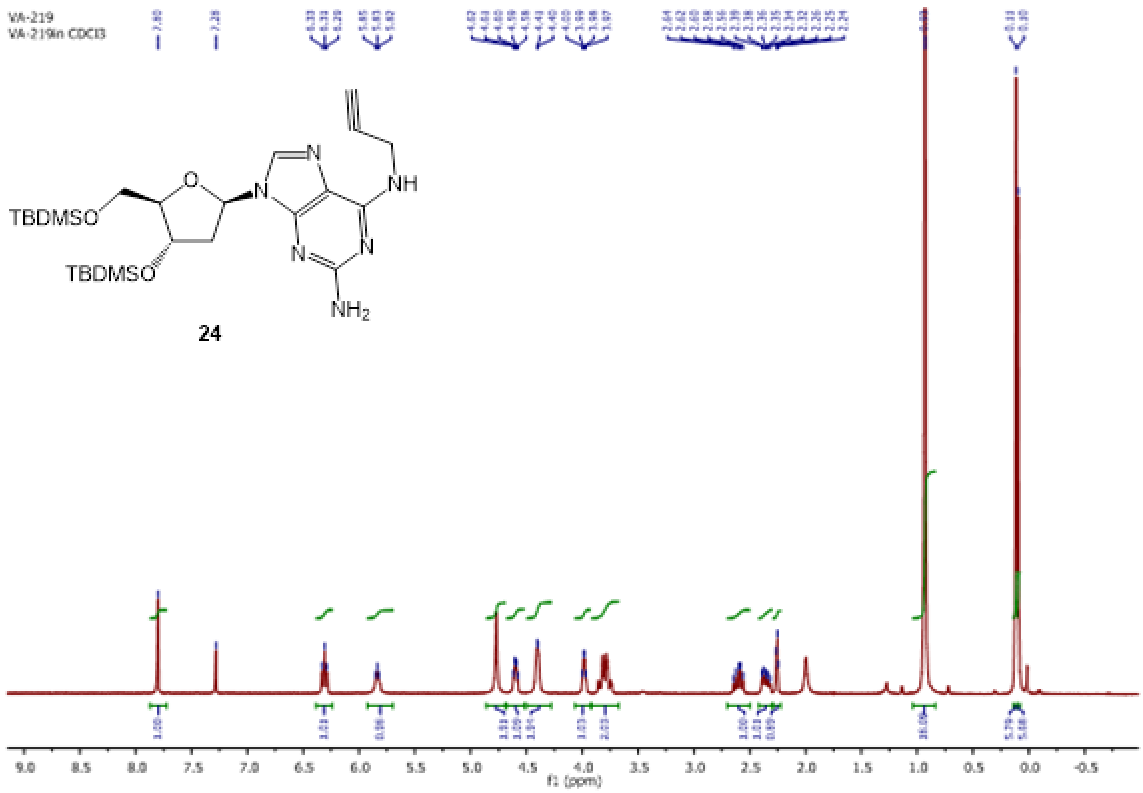

VA-219
VA-219in CDCl3
TBDMSO
TBDMSO
NH
NH2
24
f1 (ppm)

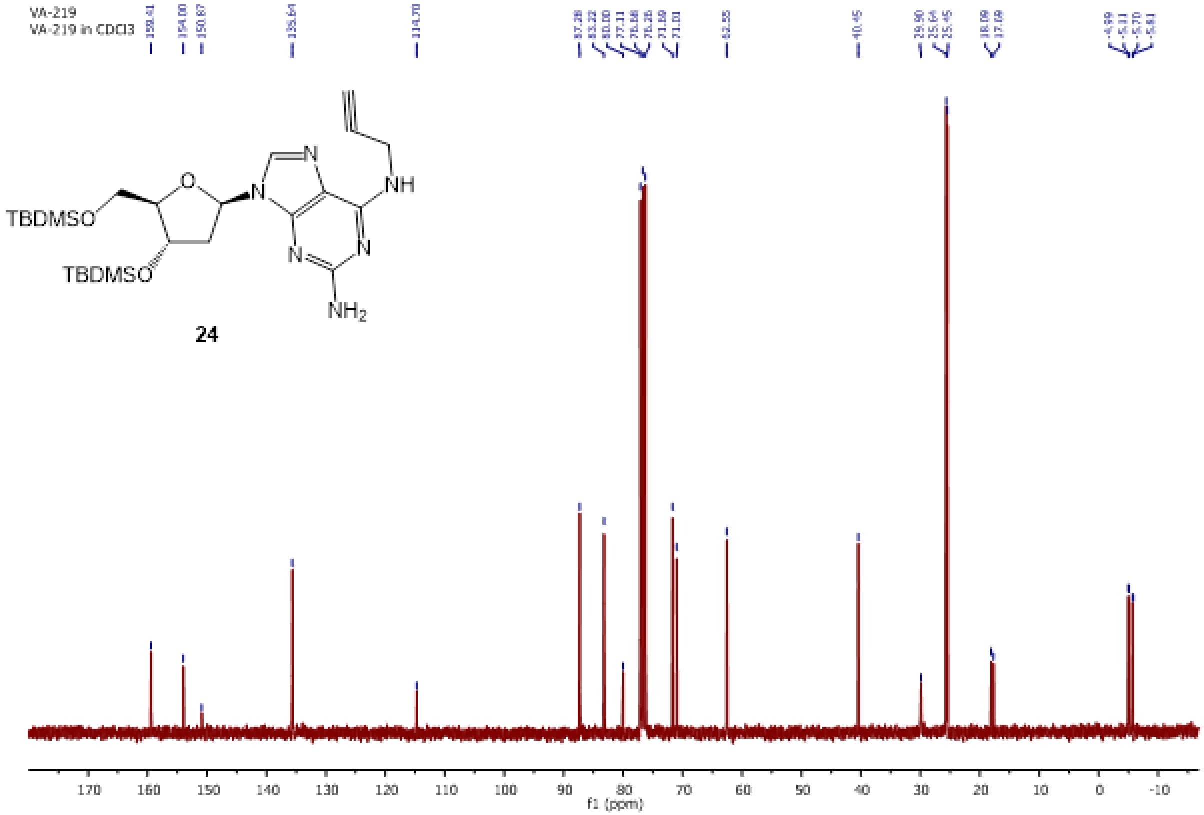

VA-219
VA-219 in CDCl3
TBDMSO
TBDMSO
NH
NH2
24
f1 (ppm)

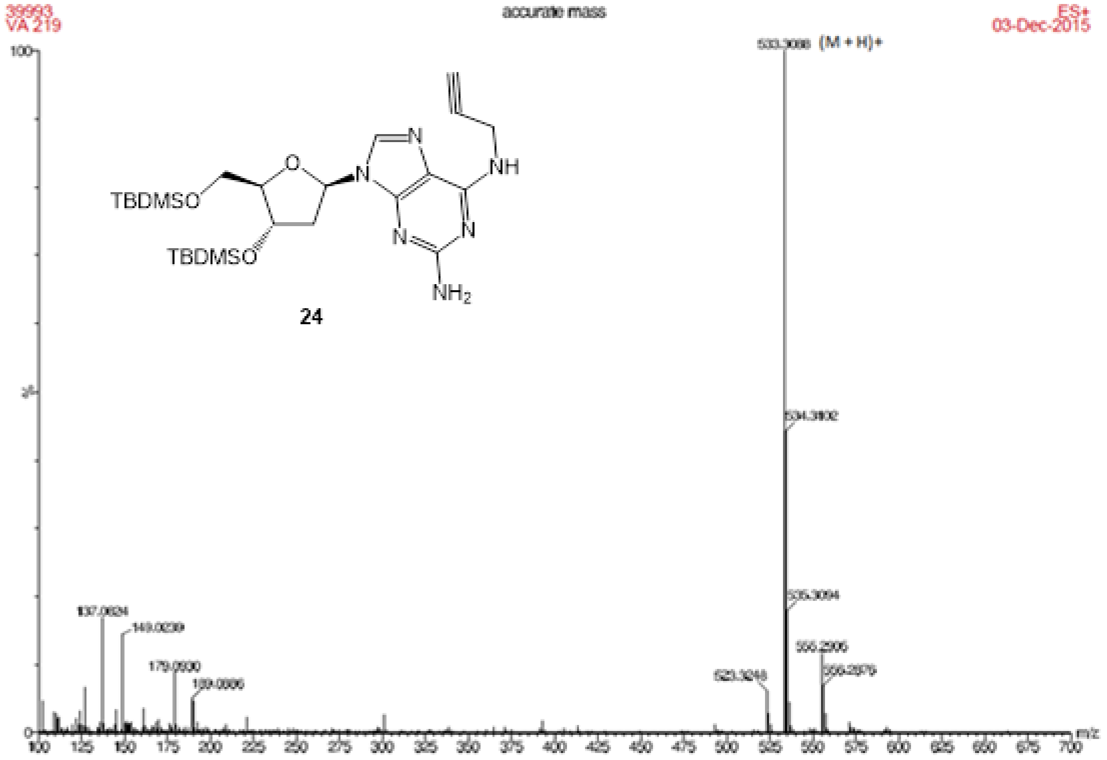

39993
VA 219
accurate mass
ES+
03-Dec-2015
533.3088 (M + H)+
534.3102
535.3094
556.2906
523.3248
137.0624
149.0239
TBDMSO
TBDMSO
NH
N
NH2
24
m/z

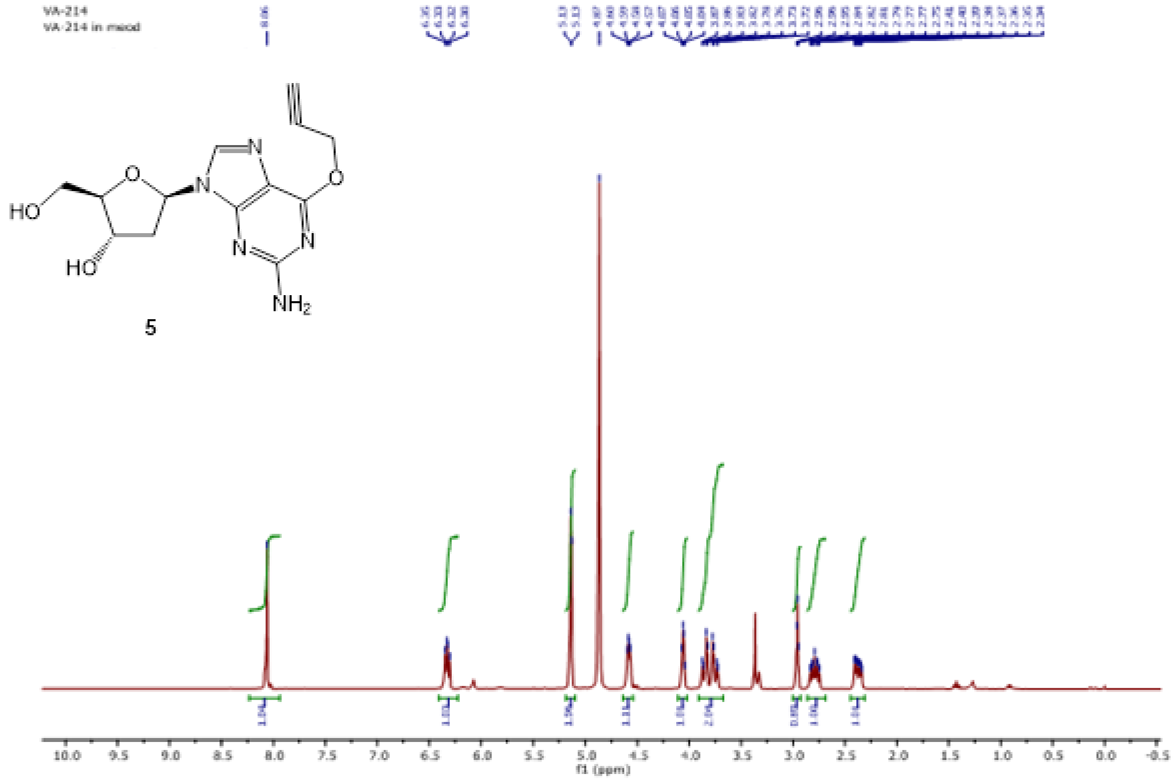

VA-214
VA-214 in meod
HO
HO
O
N
NH2
5
f1 (ppm)

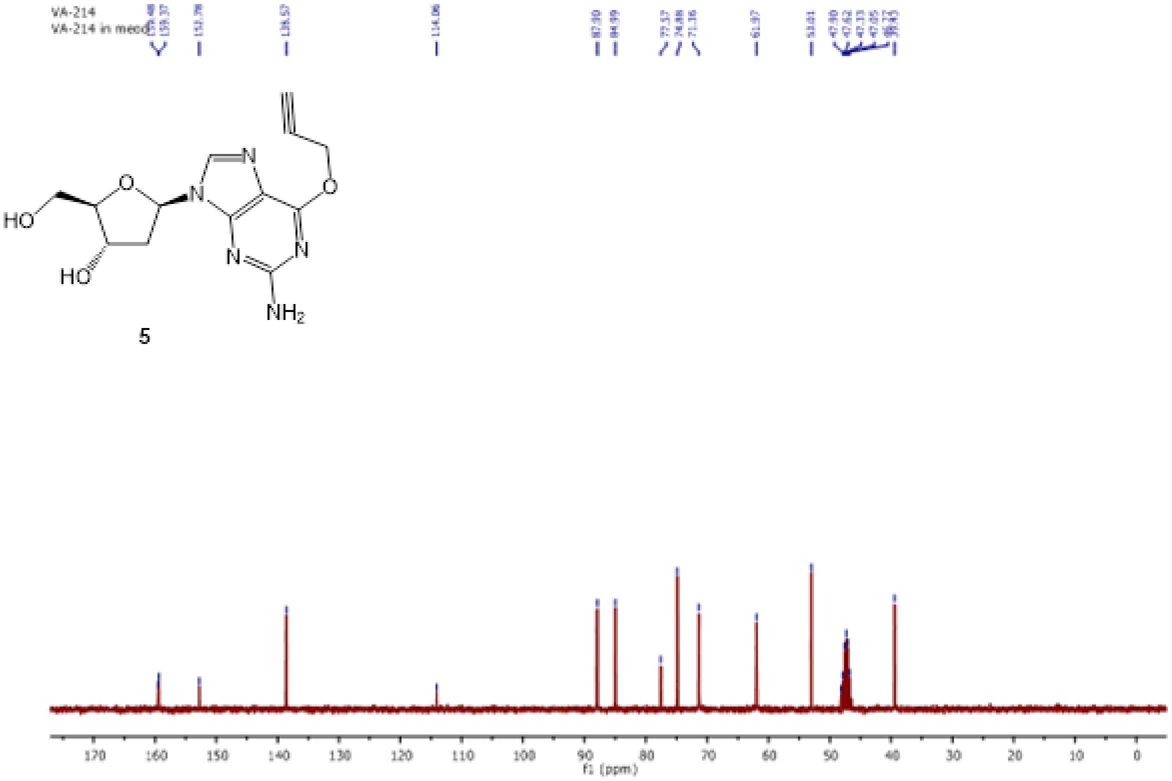
VA-214
VA-214 in meod
159.48
159.37
152.78
138.57
114.06
87.90
84.99
77.57
74.88
71.16
61.97
53.01
47.90
47.62
47.33
47.05
HO
HO
O
N
N
N
N
O
NH2
5
170 160 150 140 130 120 110 100 90 80 70 60 50 40 30 20 10 0
f1 (ppm)

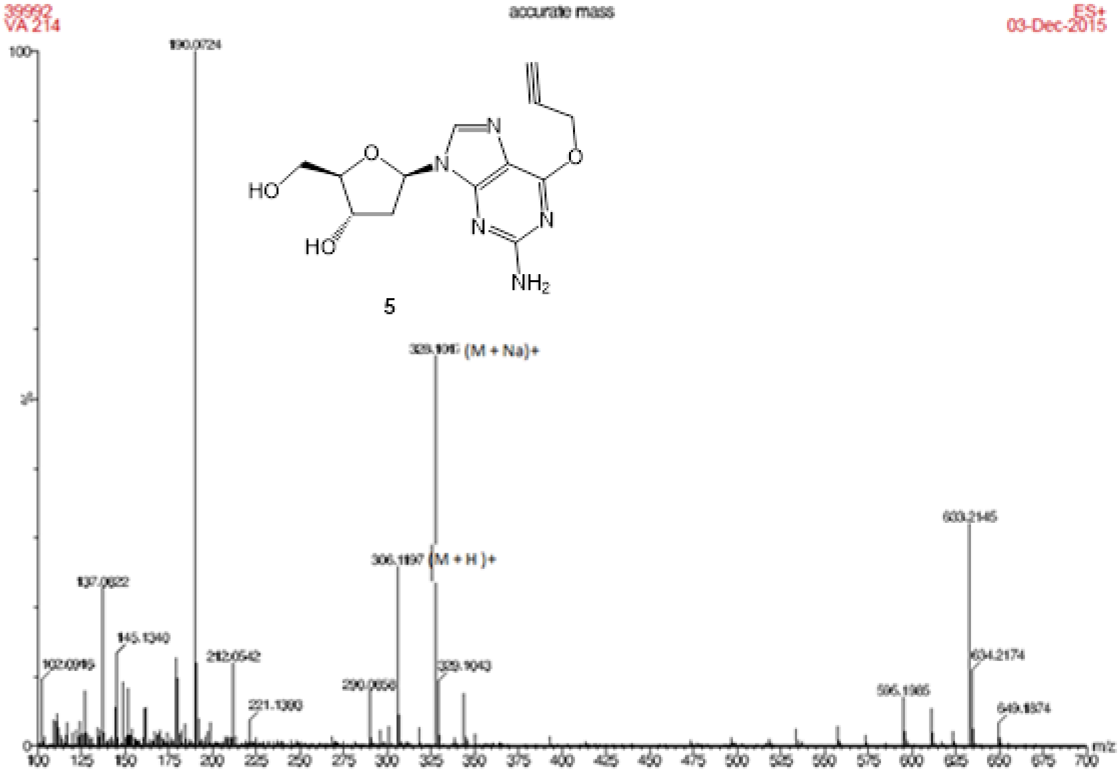
39992
VA 214
accurate mass
ES+
03-Dec-2015
100
%
0
190.0724
HO
HO
O
N
N
N
N
O
NH2
5
329.1016 (M + Na)+
633.2145
306.1197 (M + H)+
137.0922
145.1340
212.0542
102.0919
329.1043
634.2174
290.0658
596.1985
221.1390
649.1874
100 125 150 175 200 225 250 275 300 325 350 375 400 425 450 475 500 525 550 575 600 625 650 675 700
m/z

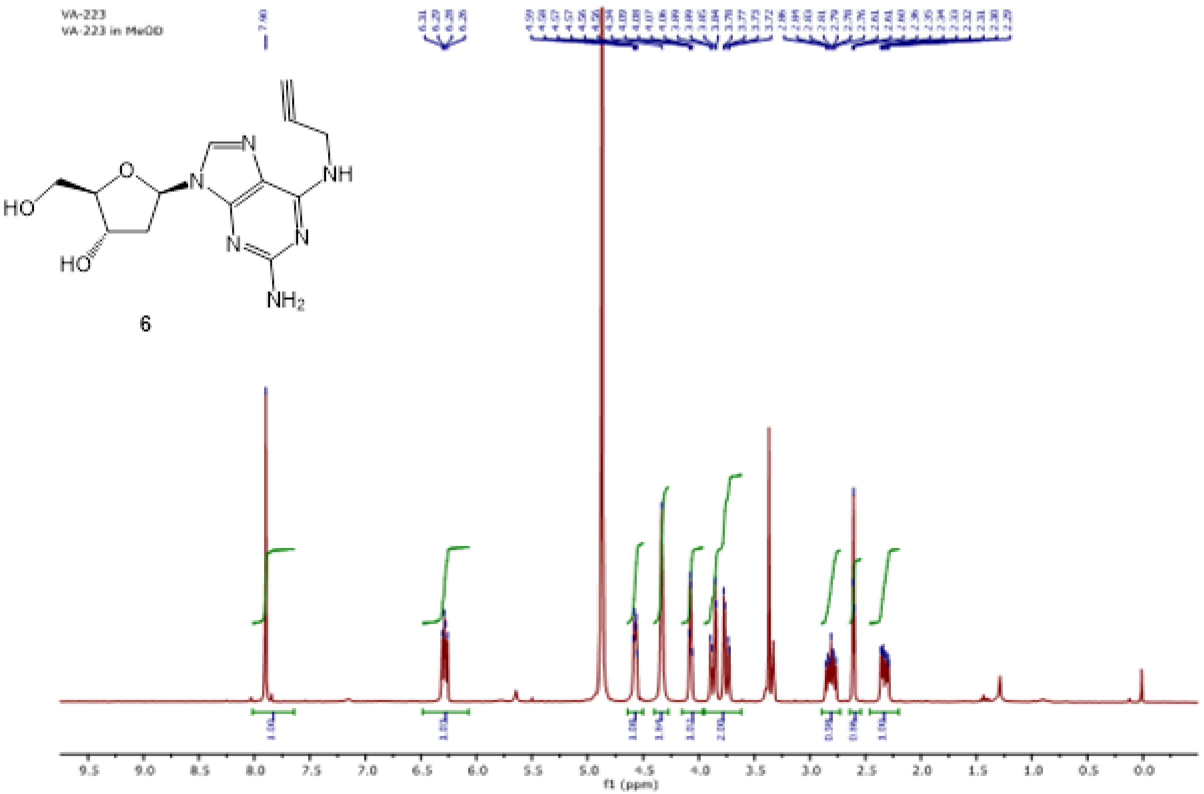
VA-223
VA-223 in MeOD
NH
HO
HO
N
NH2
6
9.5 9.0 8.5 8.0 7.5 7.0 6.5 6.0 5.5 5.0 4.5 4.0 3.5 3.0 2.5 2.0 1.5 1.0 0.5 0.0
f1 (ppm)

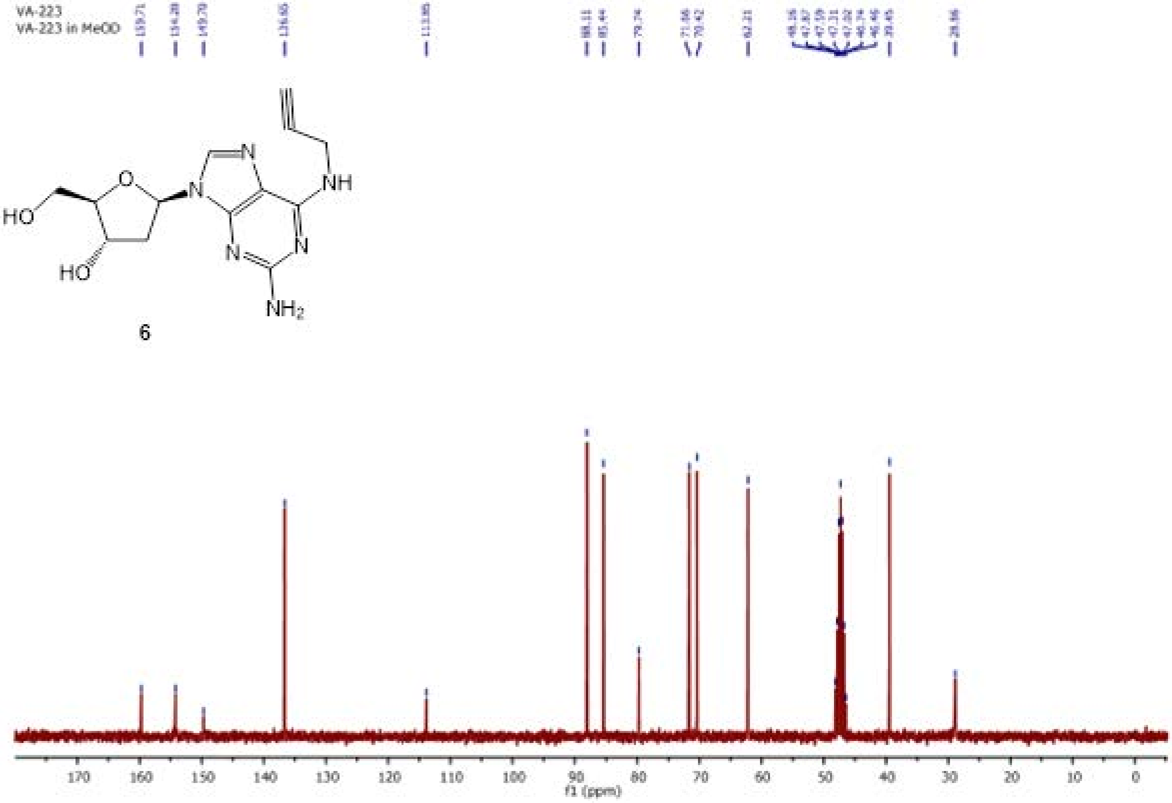
VA-223
VA-223 in MeOD
NH
HO
HO
N
NH2
6
170 160 150 140 130 120 110 100 90 80 70 60 50 40 30 20 10 0
f1 (ppm)

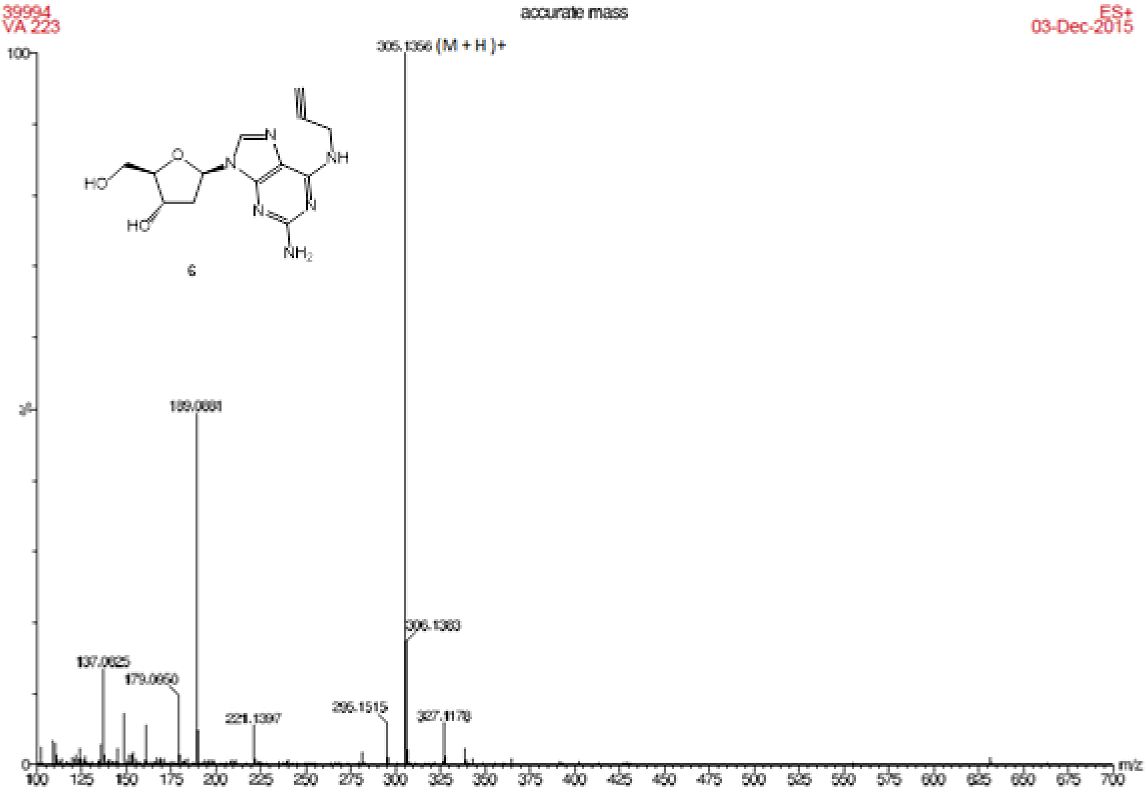
39994
VA 223
accurate mass
ES+
03-Dec-2015
305.1356 (M + H)+
189.0881
306.1363
137.0625
179.0950
221.1397
295.1515
327.1178
6
m/z

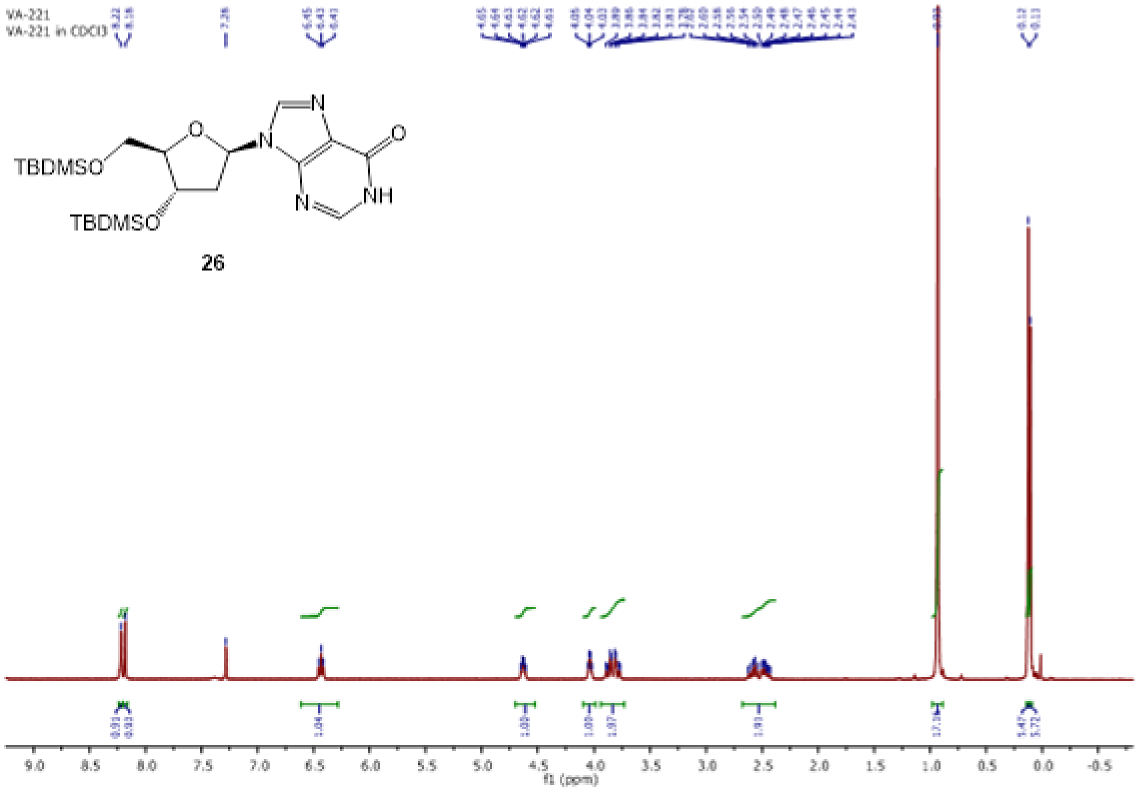
VA-221
VA-221 in CDCl3
TBDMSO
TBDMSO
26
f1 (ppm)

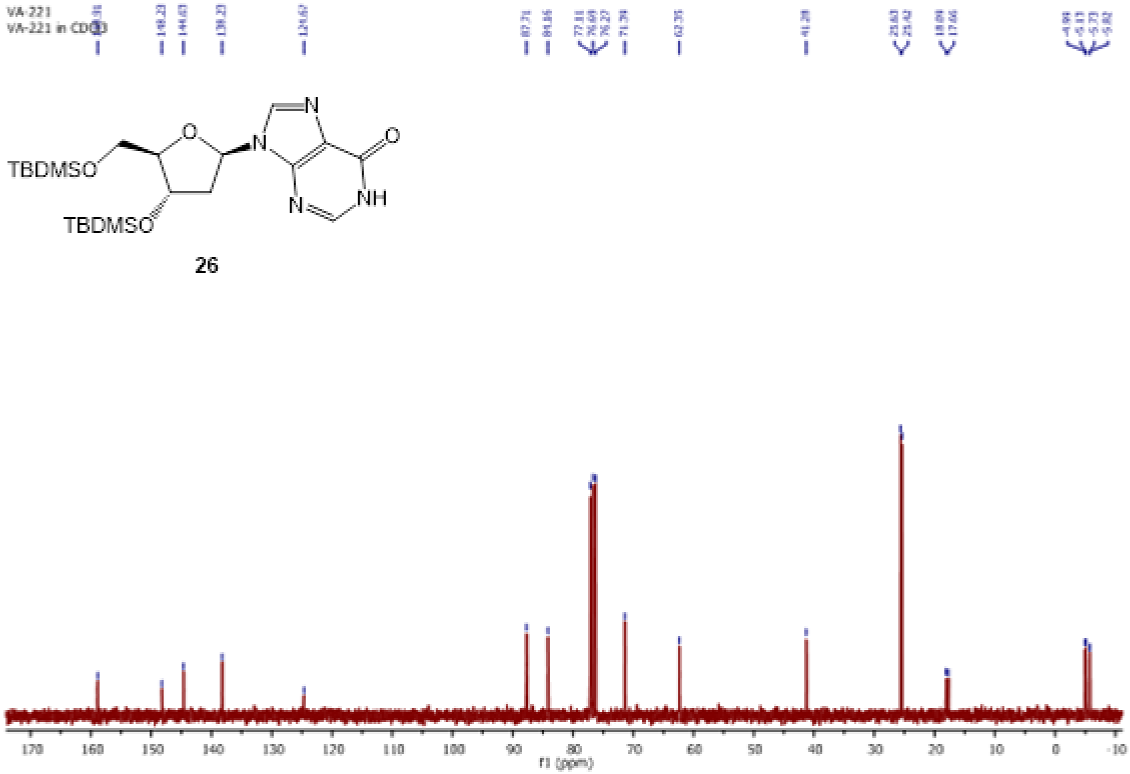
VA-221
VA-221 in CDCl3
TBDMSO
TBDMSO
N
N
NH
O
26
f1 (ppm)
170
160
150
140
130
120
110
100
90
80
70
60
50
40
30
20
10
0
-10

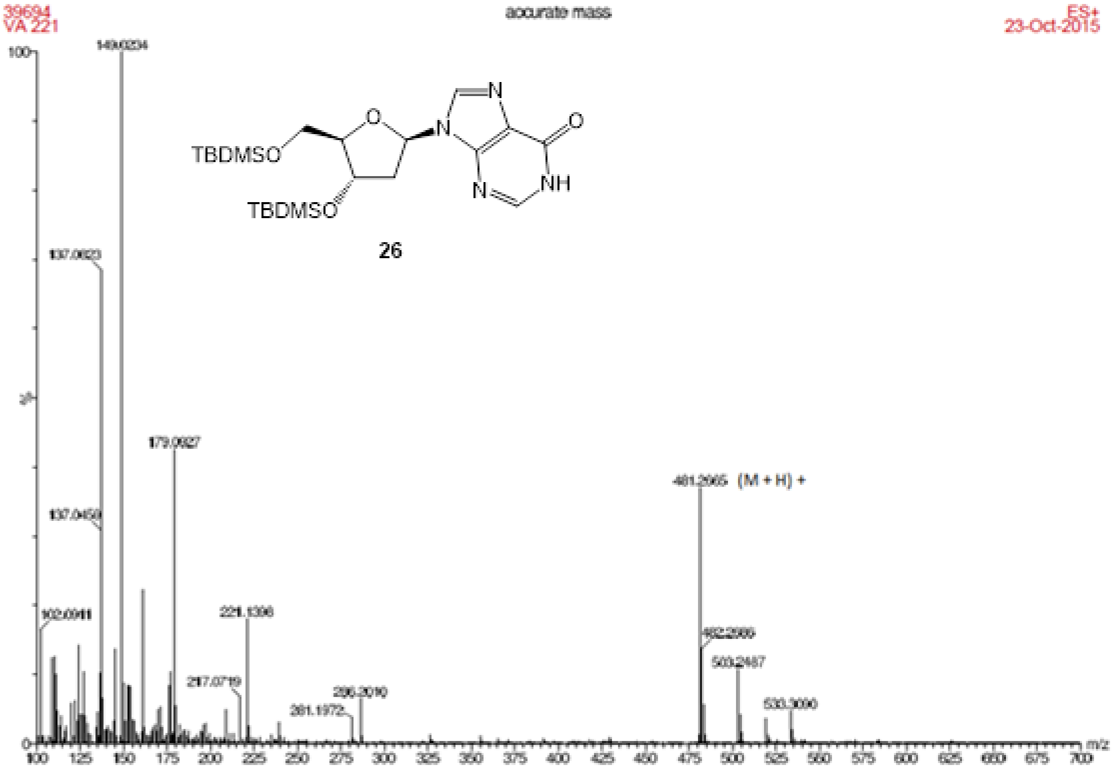
39694
VA 221
accurate mass
ES+
23-Oct-2015
TBDMSO
TBDMSO
N
N
NH
O
26
149.0234
137.0823
179.0927
137.0459
481.2965 (M + H) +
102.0911
221.1396
482.2986
503.2487
217.0719
286.2010
281.1972
533.3090
m/z
%
100
0

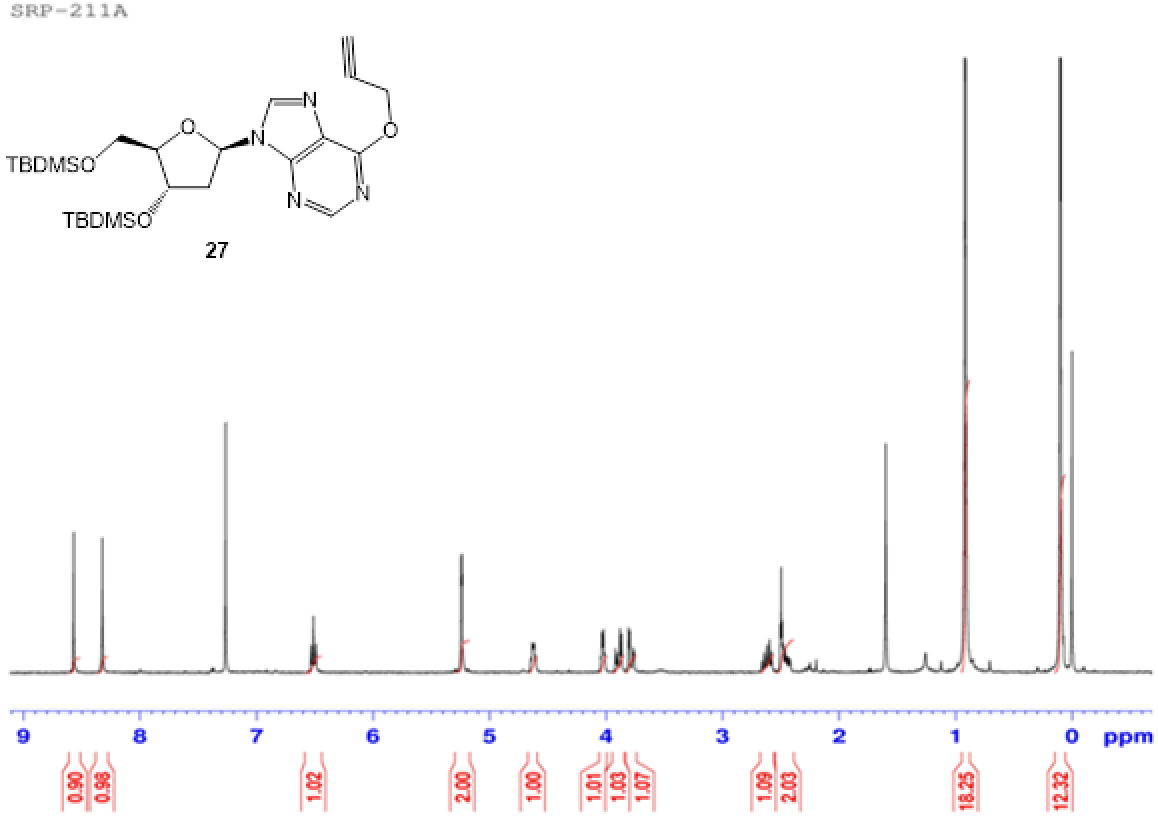
SRP-211A
TBDMSO
TBDMSO
O
N
N
N
N
O
27
9 8 7 6 5 4 3 2 1 0 ppm
0.90 0.98 1.02 2.00 1.00 1.01 1.03 1.07 1.09 2.03 18.25 12.32

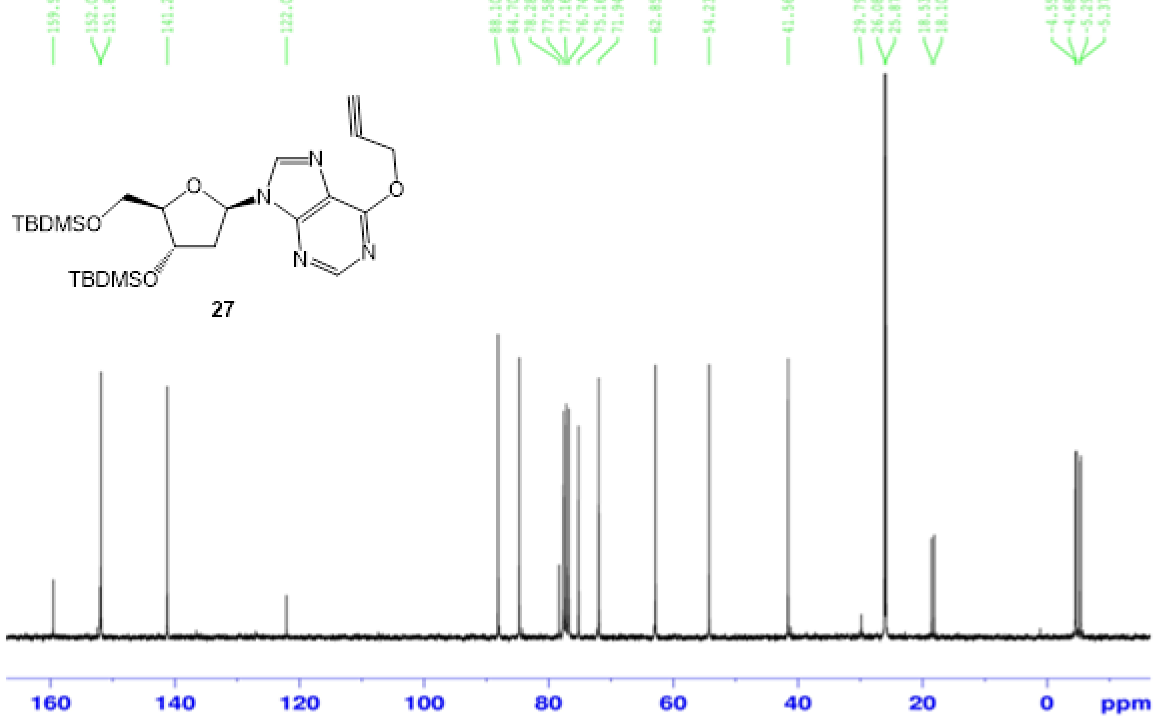
159.53 152.01 151.84 141.21 122.08 88.10 84.70 78.28 77.58 77.16 76.74 75.16 71.94 62.85 54.23 41.56 29.79 26.08 25.87 18.53 18.10 -4.55 -4.68 -5.29 -5.37
TBDMSO
TBDMSO
O
N
N
N
N
O
27
160 140 120 100 80 60 40 20 0 ppm

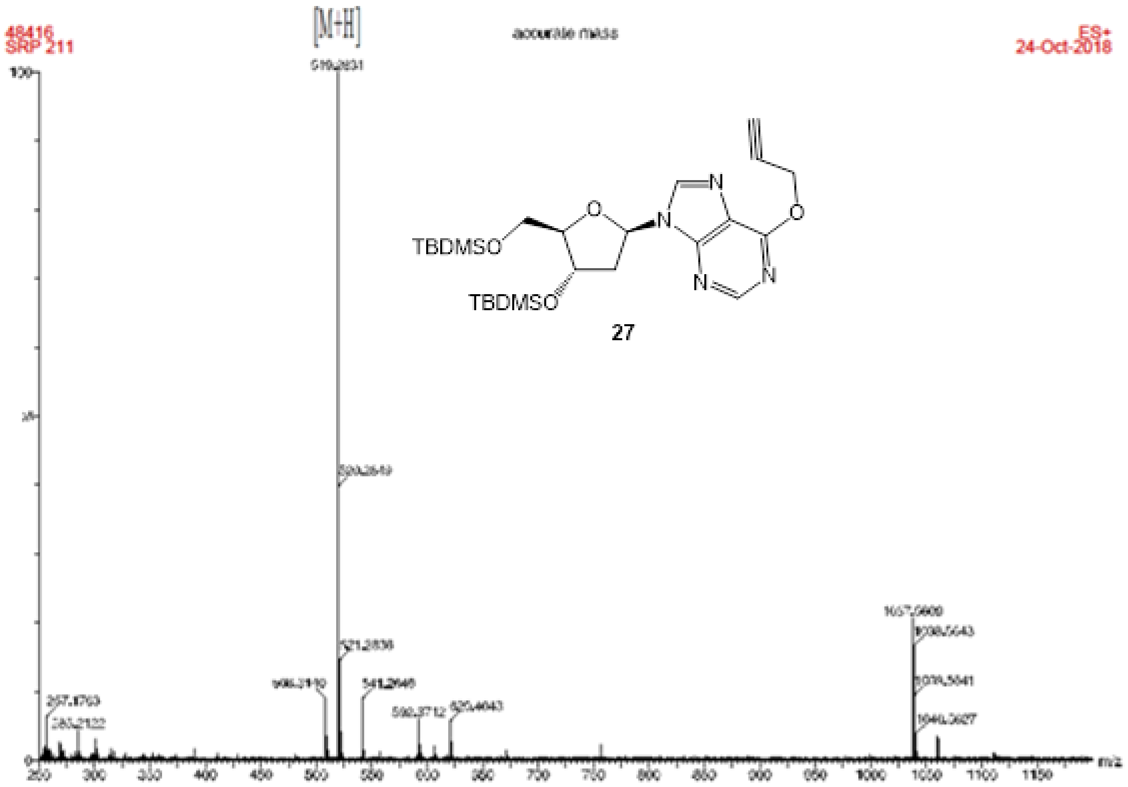
48416
SRP 211
[M+H]
accurate mass
ES+
24-Oct-2018
TBDMSO
TBDMSO
N
N
N
N
O
O
27
m/z

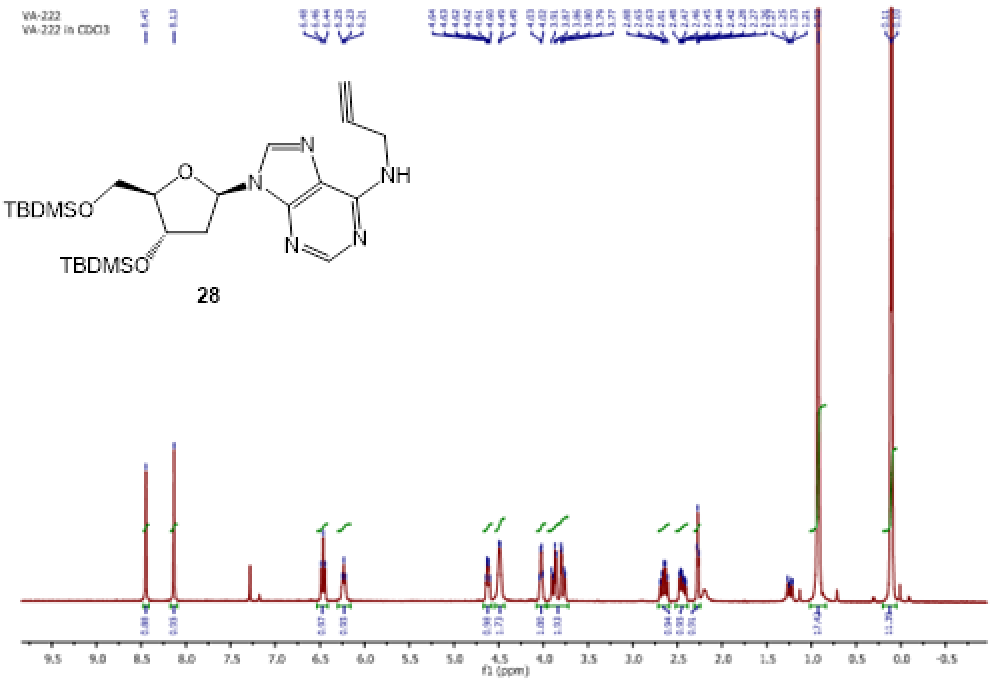
VA-222
VA-222 in CDCl3
TBDMSO
TBDMSO
NH
N
N
N
N
O
28
f1 (ppm)

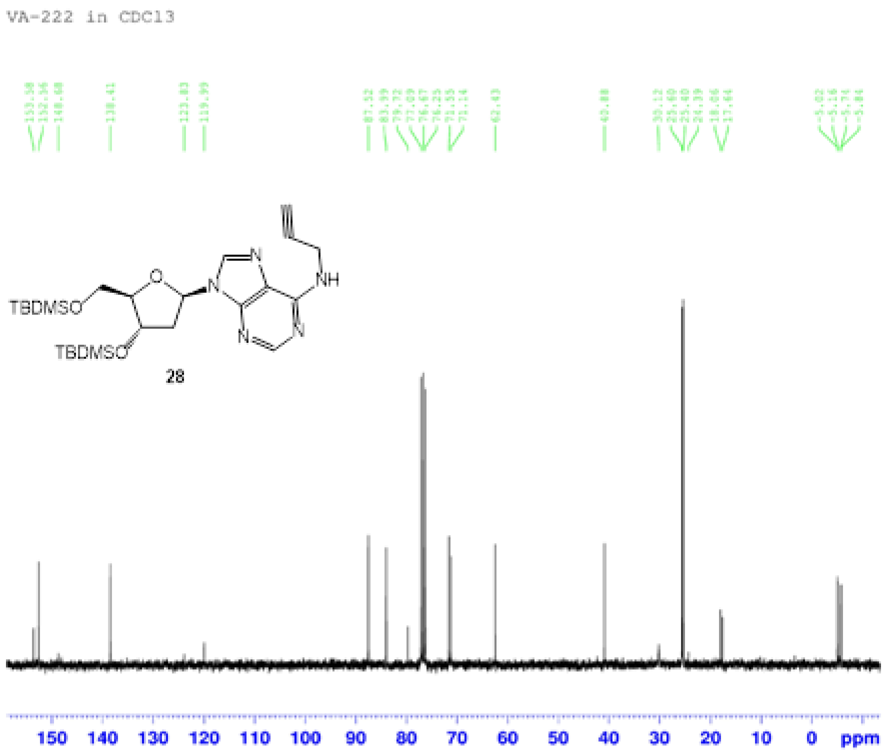
VA-222 in CDCl3
153.38
152.36
148.48
138.41
123.83
119.99
87.52
83.99
79.72
77.09
76.67
76.25
71.55
71.14
62.43
40.88
30.12
25.60
25.40
24.39
18.06
17.64
-5.02
-5.16
-5.71
-5.84
NH
N
O
TBDMSO
TBDMSO
28
150
140
130
120
110
100
90
80
70
60
50
40
30
20
10
0
ppm

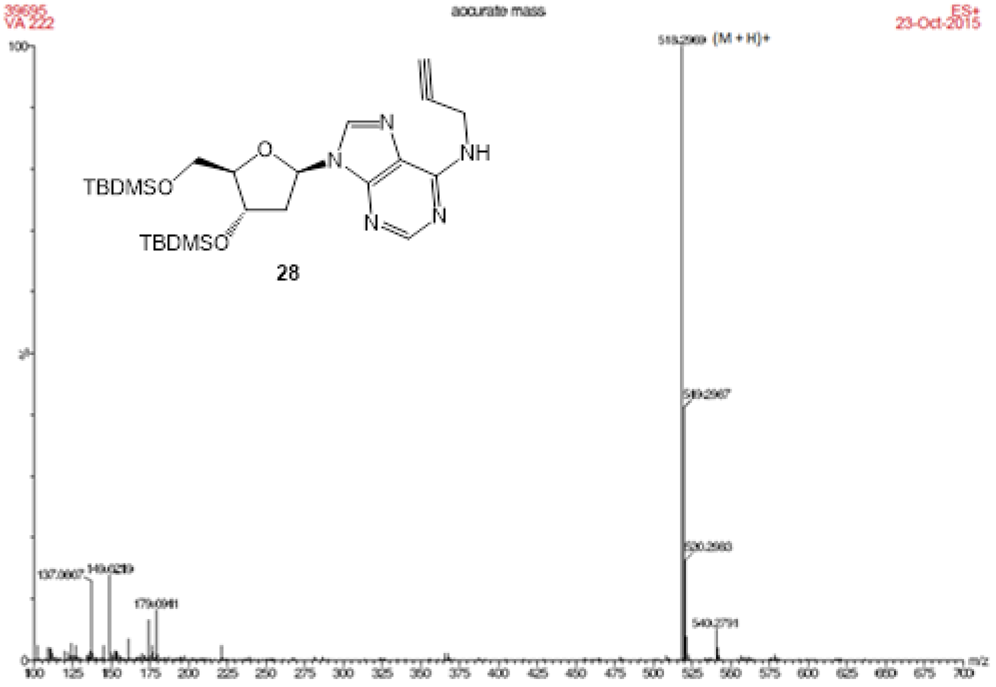
39695
VA 222
accurate mass
ES+
23-Oct-2015
(M + H)+
NH
N
O
TBDMSO
TBDMSO
28
m/z

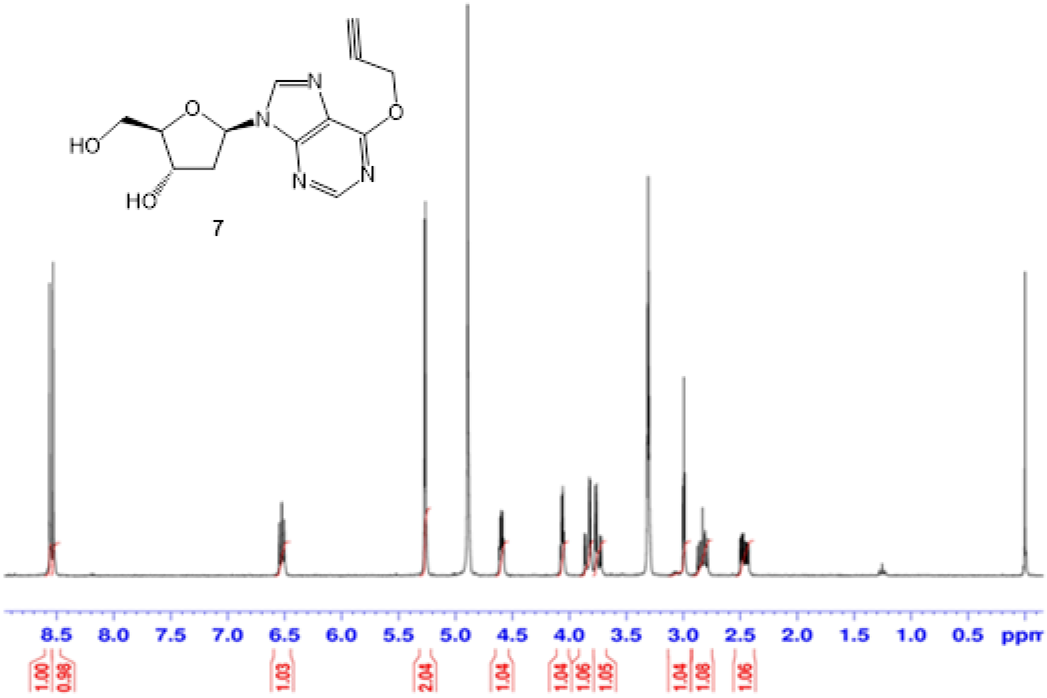

HO
HO
O
N
N
N
N
O
7
8.5 8.0 7.5 7.0 6.5 6.0 5.5 5.0 4.5 4.0 3.5 3.0 2.5 2.0 1.5 1.0 0.5 ppm
1.00 0.98 1.03 2.04 1.04 1.04 1.06 1.05 1.04 1.08 1.06

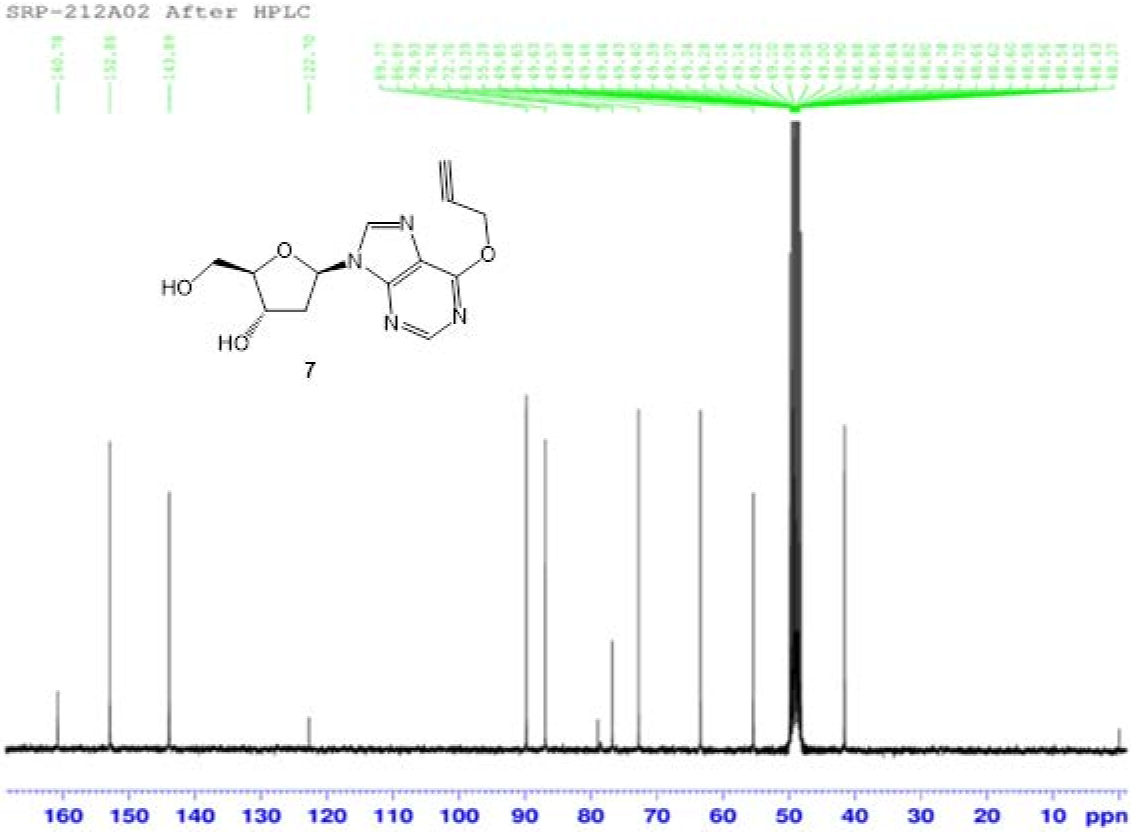

SRP-212A02 After HPLC
HO
HO
O
N
N
N
N
O
7
160 150 140 130 120 110 100 90 80 70 60 50 40 30 20 10 ppm

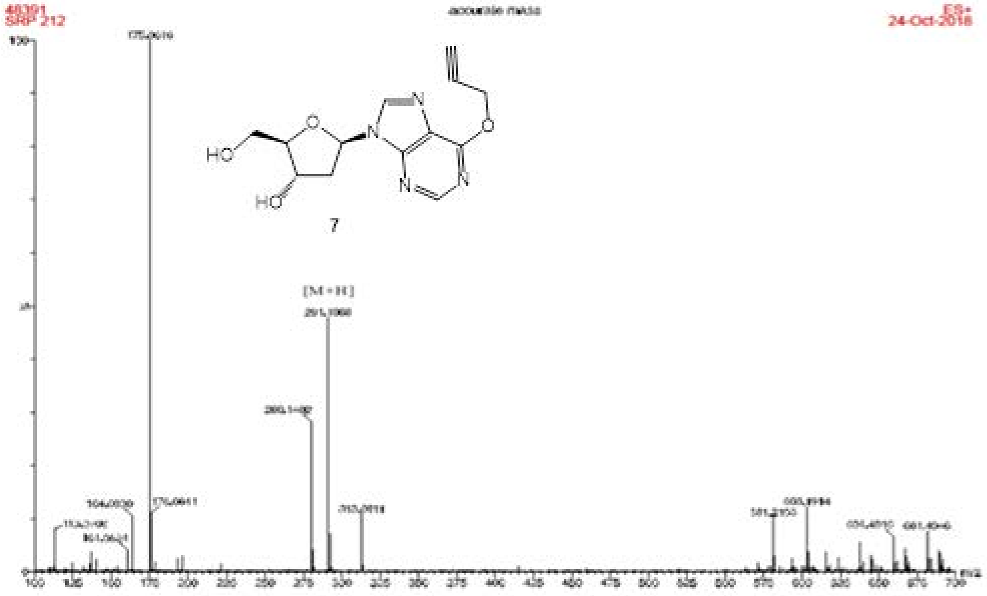

48391
SRP 212
accurate mass
ES+
24-Oct-2018
[M+H]
7

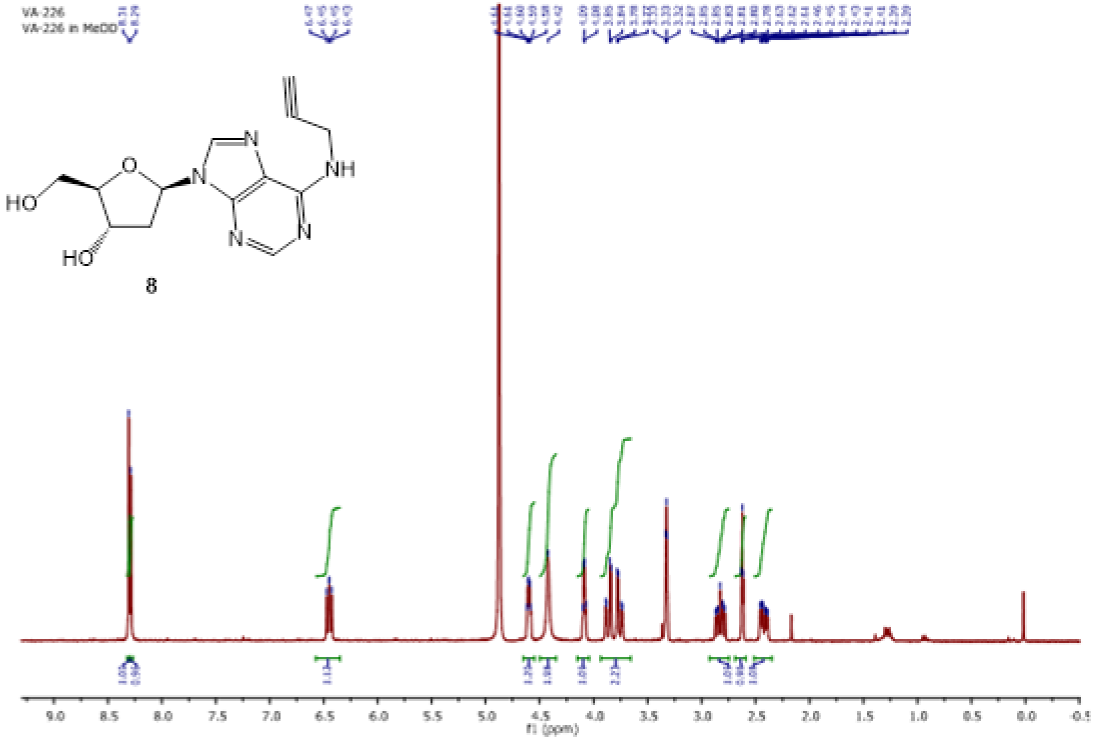

VA-226
VA-226 in MeOD
NH
8
f1 (ppm)

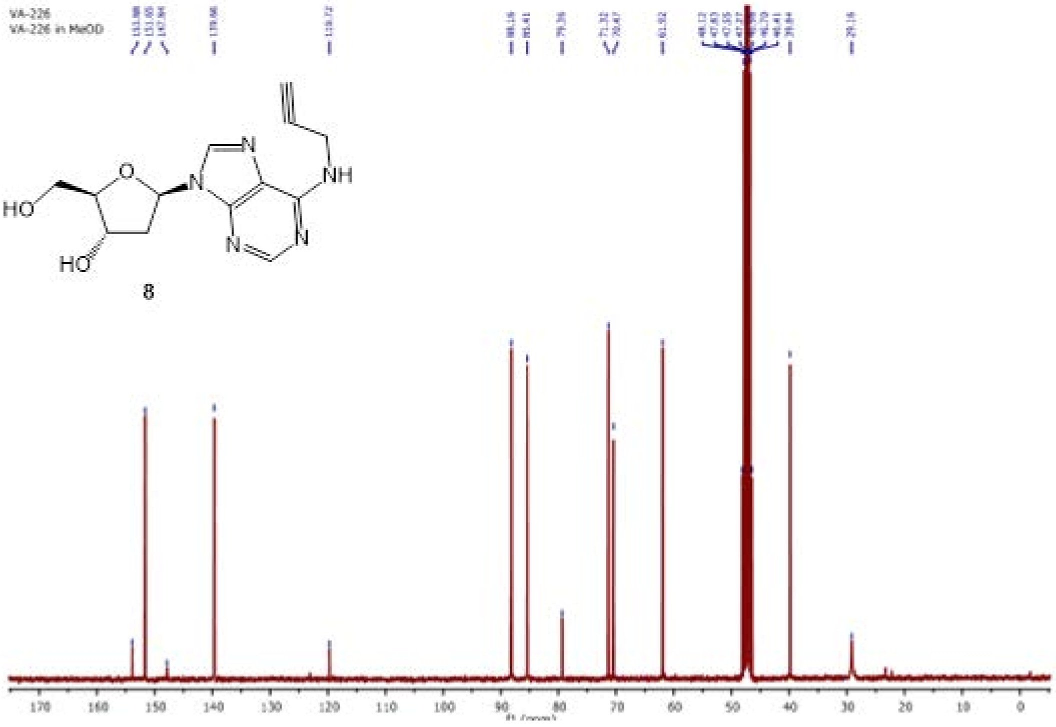
VA-226
VA-226 in MeOD
8
f1 (ppm)

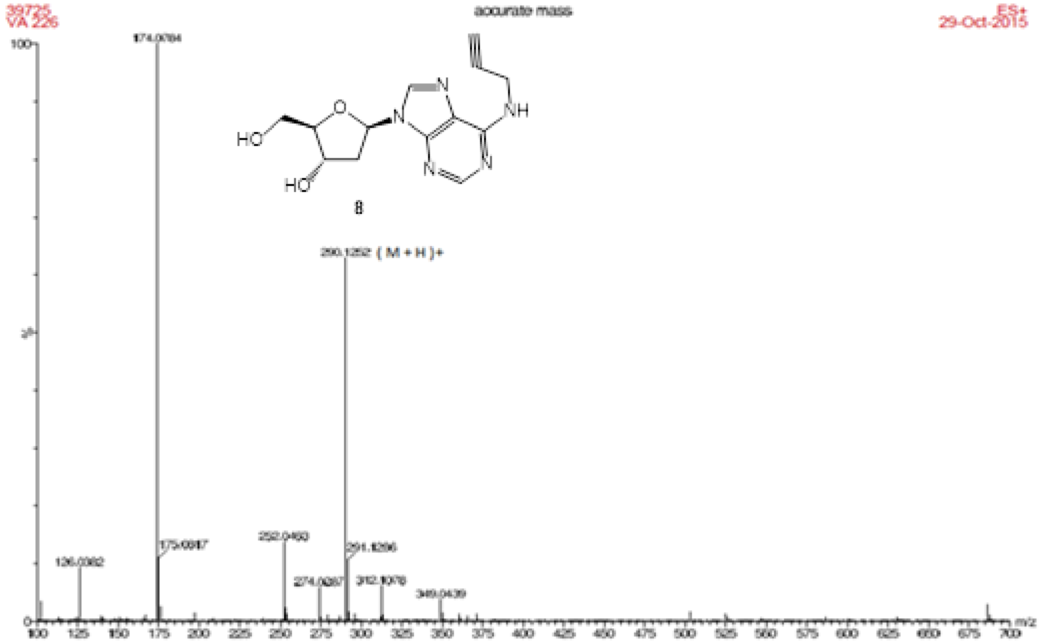
39725
VA 226
accurate mass
ES+
29-Oct-2015
8
174.0784
290.1252 (M + H)+
252.0460
126.0382
274.0267
312.1076
349.0439
m/z

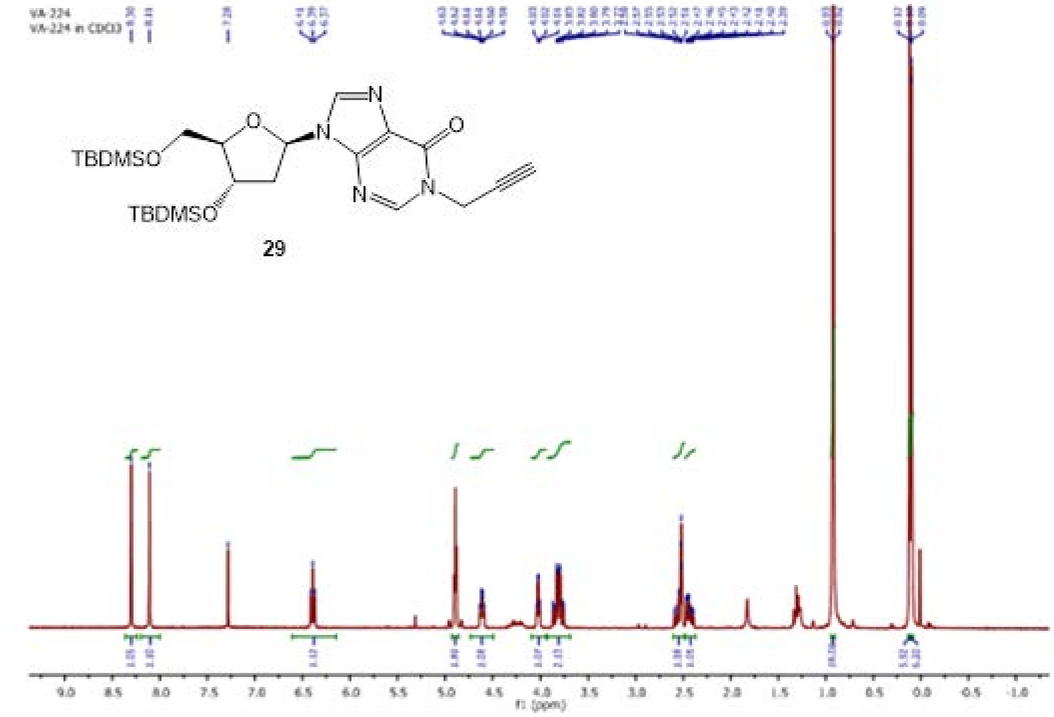
VA-224
VA-224 in CDCl3
TBDMSO
TBDMSO
N
N
N
N
O
O
29
f1 (ppm)

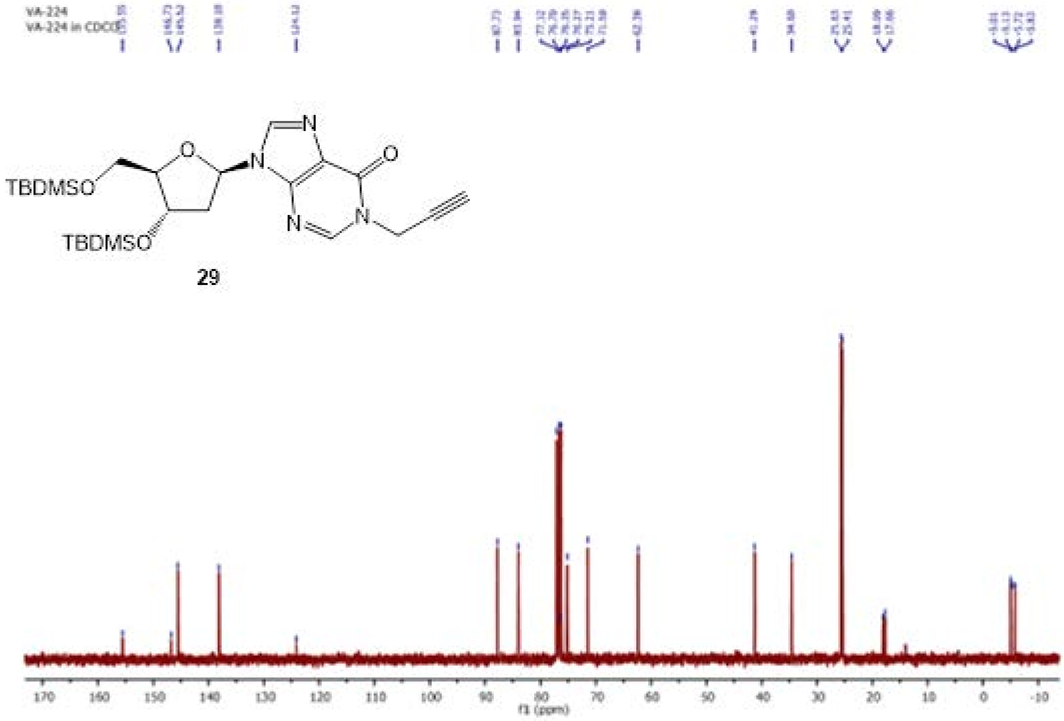
VA-224
VA-224 in CDCl3
TBDMSO
TBDMSO
N
N
N
N
O
O
29
f1 (ppm)

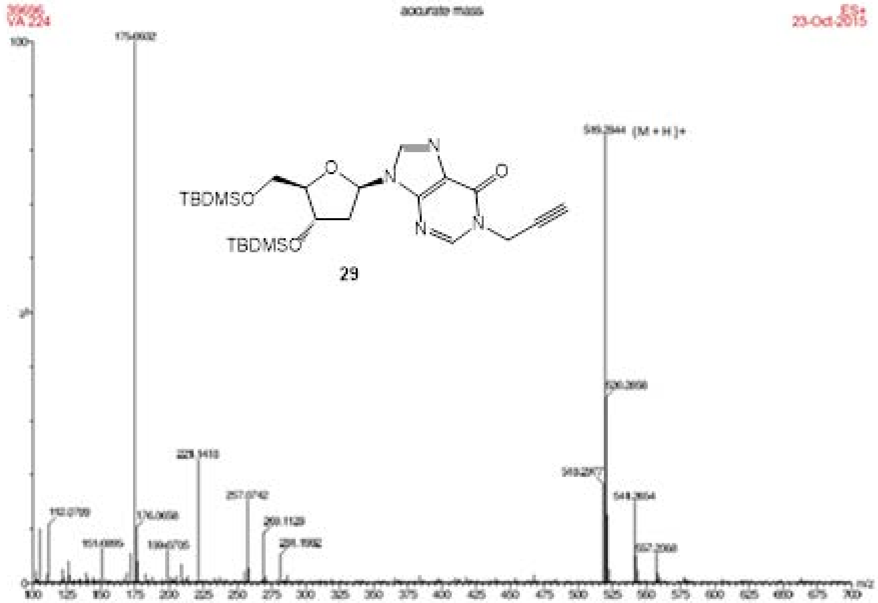

VA-225 in MeOD

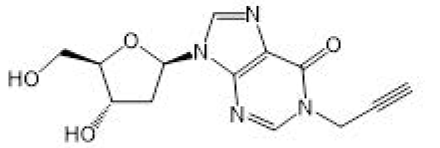

10

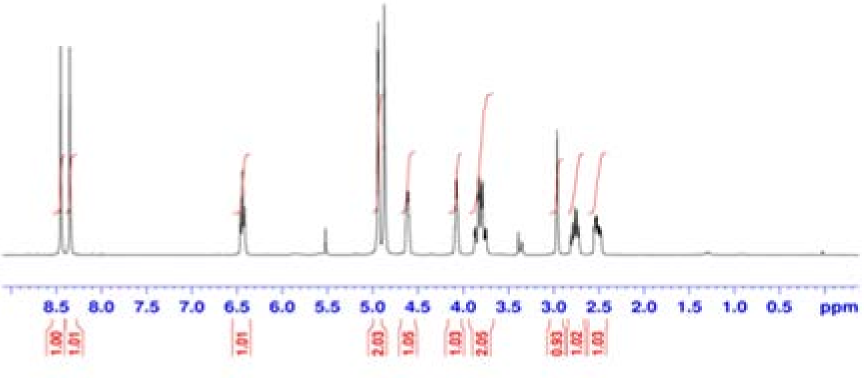

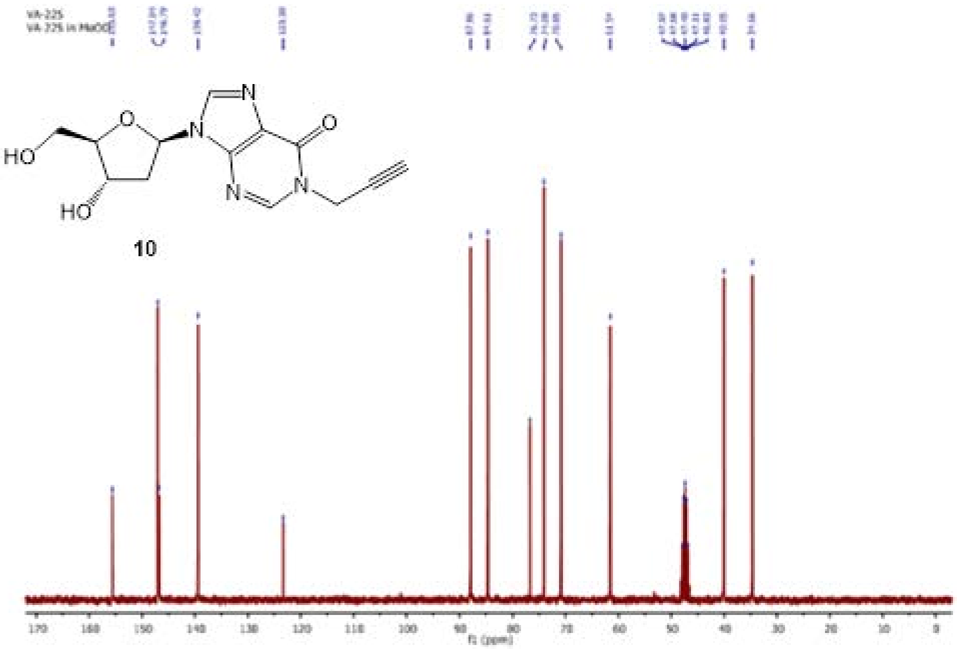
VA-225
VA-225 in MeOD
HO
HO
O
N
N
N
O
N
10
f1 (ppm)

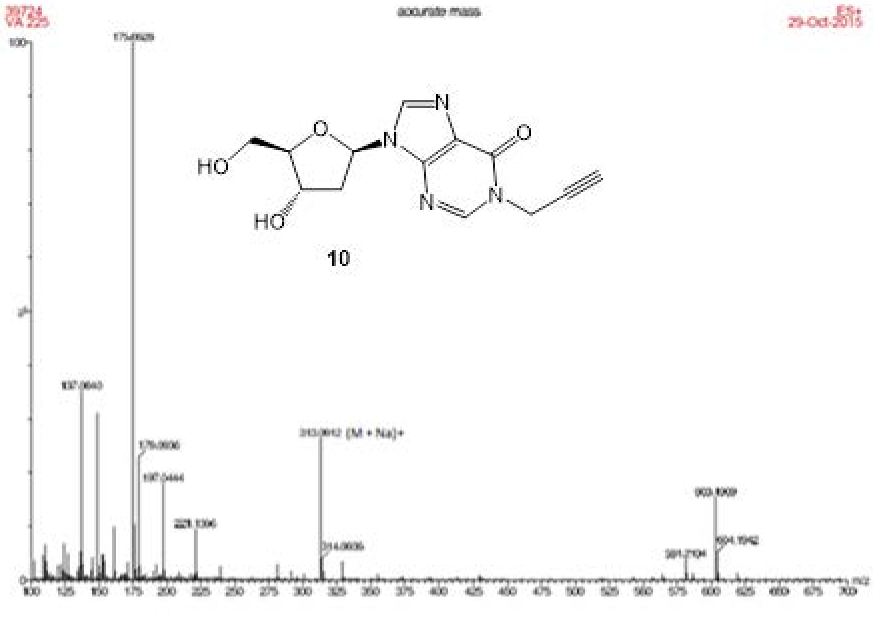
VA 225
accurate mass
ES+
29-Oct-2015
HO
HO
O
N
N
N
O
N
10
(M + Na)+

2D NMR – HMBC

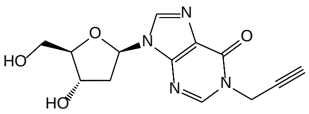

10

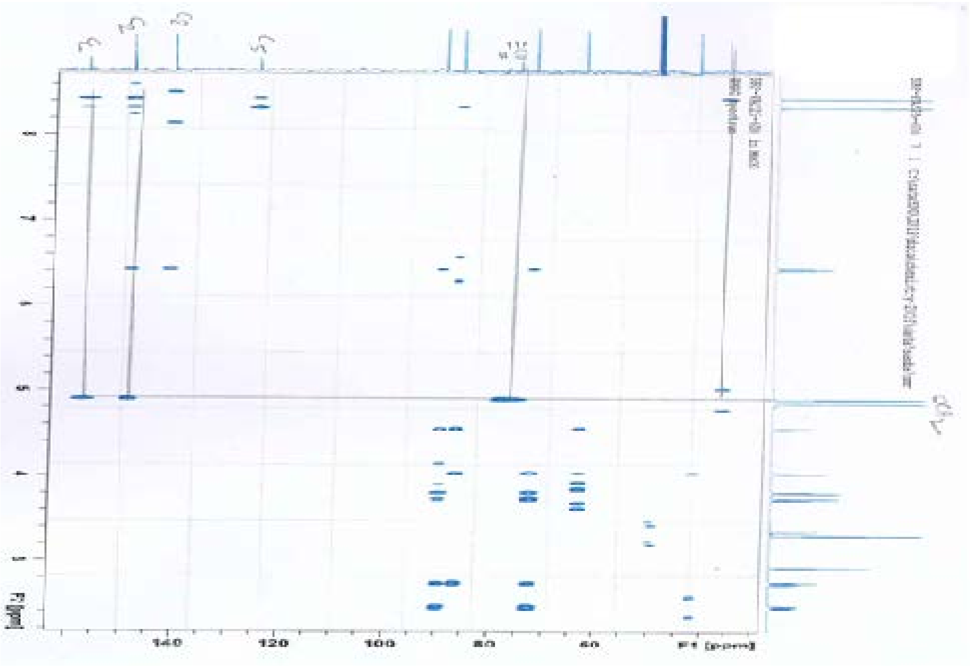

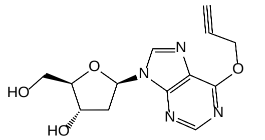

7

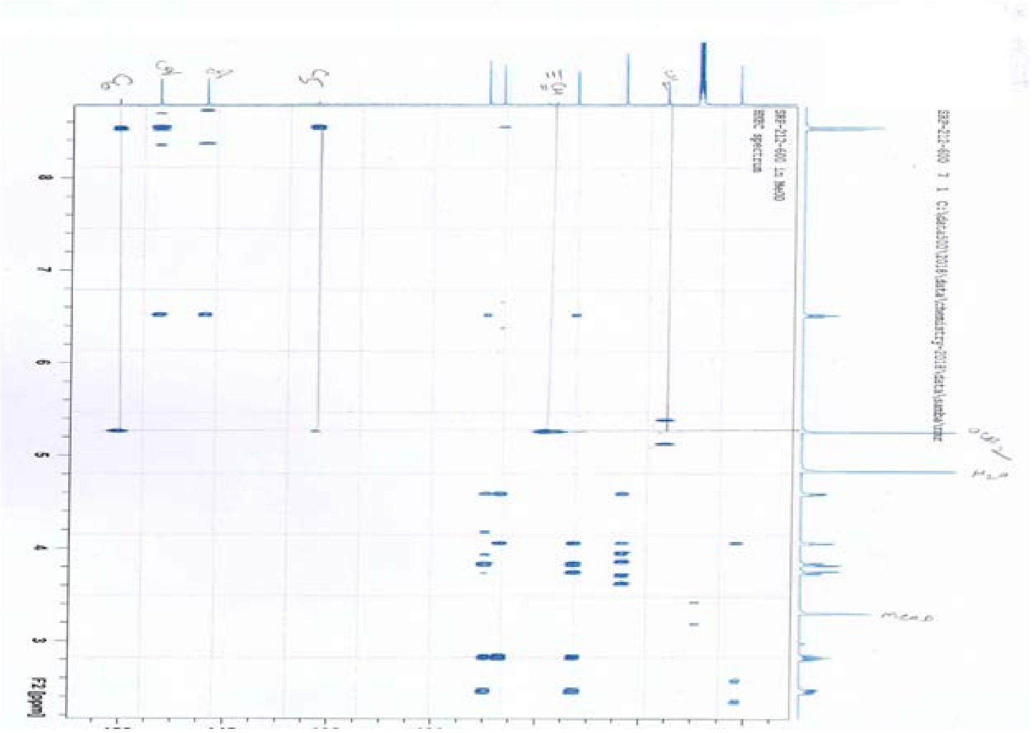

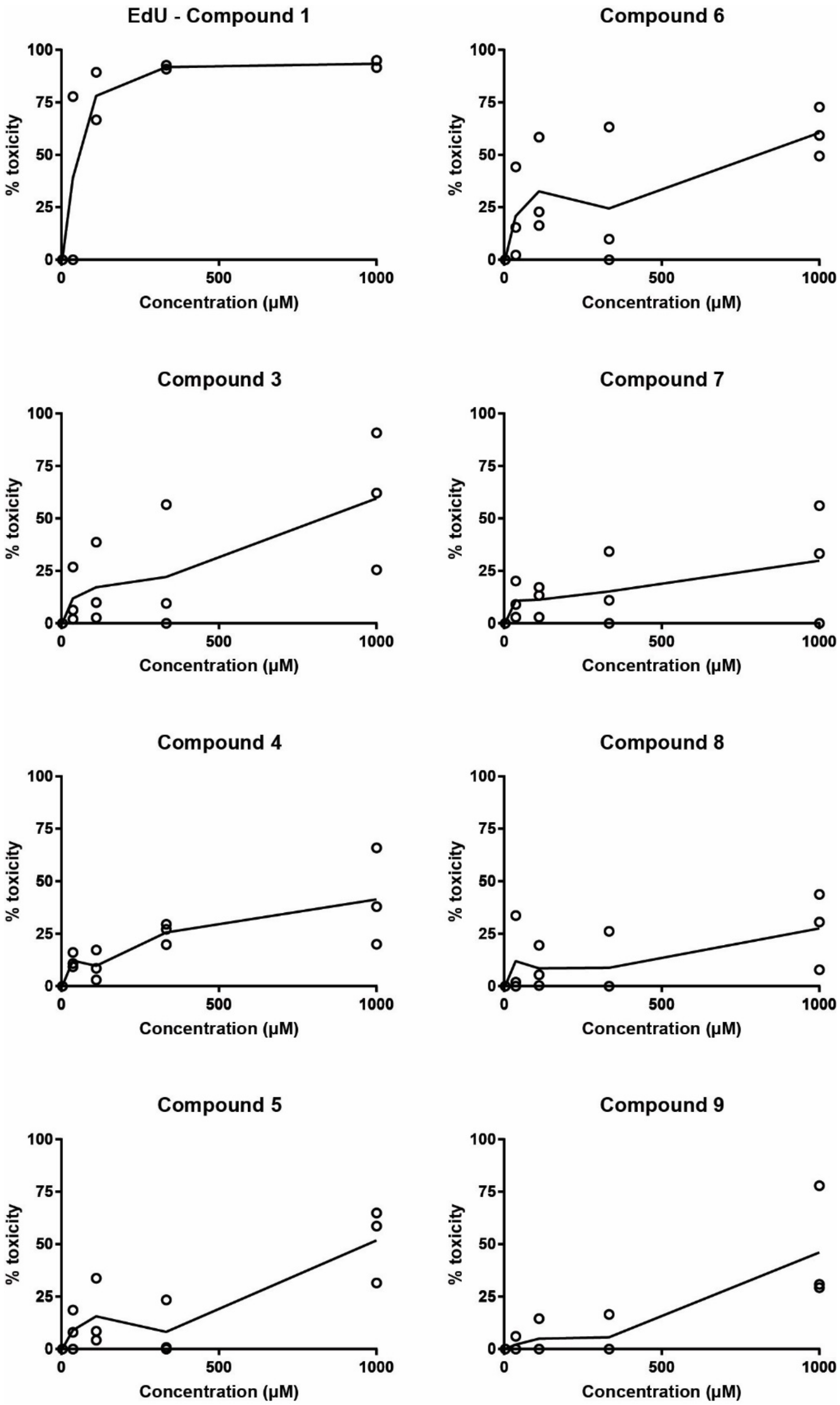

Supplementary figure: cell viability dose-response curves for the various new analogues and for EdU as a control.
